# The nasal and gut microbiome in Parkinson's disease and idiopathic rapid eye movement sleep behavior disorder

**DOI:** 10.1002/mds.27105

**Published:** 2017-08-26

**Authors:** Anna Heintz‐Buschart, Urvashi Pandey, Tamara Wicke, Friederike Sixel‐Döring, Annette Janzen, Elisabeth Sittig‐Wiegand, Claudia Trenkwalder, Wolfgang H. Oertel, Brit Mollenhauer, Paul Wilmes

**Affiliations:** ^1^ Eco‐Systems Biology Research Group, Luxembourg Centre for Systems Biomedicine (LCSB) University of Luxembourg Esch‐sur‐Alzette Luxembourg; ^2^ Paracelsus‐Elena‐Klinik Kassel Germany; ^3^ Department of Neurology Philipps University Marburg Germany; ^4^ University Medical Center Goettingen, Department of Neurosurgery Goettingen Germany; ^5^ University Medical Center Goettingen, Department of Neurology Goettingen Germany

**Keywords:** PD, RBD, nonmotor phenotype, 16S rRNA gene amplicon sequencing, genome reconstructions

## Abstract

**Background:**

Increasing evidence connects the gut microbiota and the onset and/or phenotype of Parkinson's disease (PD). Differences in the abundances of specific bacterial taxa have been reported in PD patients. It is, however, unknown whether these differences can be observed in individuals at high risk, for example, with idiopathic rapid eye movement sleep behavior disorder, a prodromal condition of α‐synuclein aggregation disorders including PD.

**Objectives:**

To compare microbiota in carefully preserved nasal wash and stool samples of subjects with idiopathic rapid eye movement sleep behavior disorder, manifest PD, and healthy individuals.

**Methods:**

Microbiota of flash‐frozen stool and nasal wash samples from 76 PD patients, 21 idiopathic rapid eye movement sleep behavior disorder patients, and 78 healthy controls were assessed by 16S and 18S ribosomal RNA amplicon sequencing. Seventy variables, related to demographics, clinical parameters including nonmotor symptoms, and sample processing, were analyzed in relation to microbiome variability and controlled differential analyses were performed.

**Results:**

Differentially abundant gut microbes, such as *Akkermansia*, were observed in PD, but no strong differences in nasal microbiota. Eighty percent of the differential gut microbes in PD versus healthy controls showed similar trends in idiopathic rapid eye movement sleep behavior disorder, for example, *Anaerotruncus* and several *Bacteroides* spp., and correlated with nonmotor symptoms. Metagenomic sequencing of select samples enabled the reconstruction of genomes of so far uncharacterized differentially abundant organisms.

**Conclusion:**

Our study reveals differential abundances of gut microbial taxa in PD and its prodrome idiopathic rapid eye movement sleep behavior disorder in comparison to the healthy controls, and highlights the potential of metagenomics to identify and characterize microbial taxa, which are enriched or depleted in PD and/or idiopathic rapid eye movement sleep behavior disorder. © 2017 The Authors. Movement Disorders published by Wiley Periodicals, Inc. on behalf of International Parkinson and Movement Disorder Society.

The classical pathological hallmarks of PD are Lewy bodies and Lewy neurites comprised of aggregations of the protein α‐synuclein (aSyn). The latter are considered to lead to neuronal loss in the dopaminergic SN of the central nervous system (CNS). Relatively recently, aSyn aggregation has also been observed in the peripheral nervous system (PNS).^1^, ^2^, ^3^, ^4^, ^5^ aSyn aggregation in the autonomic plexus of the intestine is even hypothesized to represent a main source of the disease.^1^, ^2^, ^3^, ^4^, ^5^, ^6^ In an epidemiological study, a decreased risk for subsequent Parkinson's disease (PD) was shown after complete (but not selective) truncal vagotomy.^7^ This suggests that the vagal nerve may be critically involved in the aSyn pathology of PD apparently ascending from the PNS to the CNS.^8^ Furthermore, inflammation and increased permeability of the mucosal lining of the colon have been shown in PD.^1^, ^9^ Both conditions are associated with the function of the gut microbiota, whose members might therefore play the roles of “drivers, perpetuators and mediators” of PD.^10^ The olfactory bulb is also affected by aSyn pathology in very early stages, reflected by hyposmia.^6^, ^8^ Based on these observations, it has been suggested that a currently unknown infectious or otherwise transmissible agent may enter the brain through the nasal cavity and/or gastrointestinal tract and initiate the ascending pathological process to the CNS as postulated in the “dual‐hit hypothesis.”^11^ In animal models, toxins can reach the CNS from the olfactory bulb^12^ and aSyn aggregation triggered by toxins or bacterial amyloid proteins in the enteric nervous system can spread to the CNS.^13^, ^14^

Several recent studies have reported PD‐related changes in bacterial populations in the gut,^15^, ^16^, ^17^, ^18^, ^19^, ^20^, ^21^ but different sample handling and preservation procedures as well as confounding factors^19^ may account for inconsistency^22^ between the reports. Also, less is known about the microbiome of the nasal cavity.^23^ Individuals likely to develop PD, who may already display differences in the periphery, have not been studied so far. Increasing evidence points toward a long prodromal phase of PD, that is marked by nonmotor symptoms^1^, ^9^, ^24^ and may start 20 to 30 years before the diagnosis of motor PD. Longitudinal follow‐up of subjects with idiopathic rapid eye movement sleep behavior disorder (iRBD) has shown a high specificity for later development of an aSyn aggregation disorder, including PD, with a high conversion rate.^24^, ^25^, ^26^ Comparisons of PD patients to individuals with iRBD may therefore be informative on the timing of changes, which may influence future research on the role of these microbiota in PD development and progression.

Here, we comprehensively analyze the microbiota of the nasal cavity and the gut represented by carefully preserved samples of deeply phenotyped PD patients and healthy controls (HCs), as well as iRBD patients. We identify taxa that differ (1) between PD patient populations and HCs and (2) in comparison to the prodrome iRBD and (3) within different nonmotor symptoms in PD. Based on these analyses, we use metagenomic shotgun sequencing of selected samples to elucidate the functional potential of novel microbial genomes that were observed to be differentially abundant in PD versus HCs.

## Materials and Methods

### Ethics

The collection and analysis of PD samples was part of the DeNoPa cohort that was conducted according to the Declaration of Helsinki and with informed written consent provided by all subjects. The study was approved by the ethics committee of the Physician's Board Hesse, Germany (Approval No. FF89/2008) and the ethics committee of the Faculty of Medicine, University Marburg, and has been registered at the German Register for Clinical trials (DRKS00000540) according to the World Health Organization Trial Registration Data Set. The sample analysis was approved by the *Comité National d'Ethique de Recherche* of Luxembourg (reference no.: 140174_ND).

### Cohort and Clinical Assessments

Samples were collected from the already published longitudinal DeNoPa cohort, comprising HCs and patients with PD with in‐depth clinical phenotyping.^27^, ^28^ In addition, 21 subjects with iRBD diagnosed according to the consensus criteria of the International RBD Study group^29^ and no signs for neurodegenerative disorder (by clinical examination and neuropsychological testing) were enrolled at the same study center. Comorbidities and comedication were documented. All subjects (PD, HC, and RBD) underwent deep clinical characterization, including the International Parkinson and Movement Disorder Society (MDS) Unified Parkinson's Disease rating Scale (MDS‐UPDRS I–III),^30^ assessment of autonomic dysfunction in Parkinson's disease (Scopa‐AUT)^31^ gastrointestinal part (Questions 1‐7), depression by Geriatric Depression Scale^32^ and Montgomery‐Åsberg Depression Scale (MADRS),^33^ cognition by Mini–Mental State Examination (MMSE)^34^ as well as Montréal Cognitive Assessment (MoCA),^35^ and sleepiness by Epworth Sleepiness Scale (ESS).^36^

### Sampling, Sample Processing, and DNA Extraction

All samples were collected between June 2012 and December 2015 at one study center. Collection of stool and nasal fluid was initiated at 48‐month follow‐up investigations in PD patients of the DeNoPa cohort. Only PD patients with verified clinical diagnosis after 24‐month follow‐up and after exclusion of other neurological disorders (as described)^27^ were included in this analysis. Only iRBD patients with a 2‐night video‐assisted polysomnography (PSG)^29^ were included in the study. All subjects were hospitalized for at least 24 hours for all investigations of DeNoPa (including PSG) at the same study center. Stool samples were collected into a stool specimen collector (MedAuxil) and collection tubes (Sarstedt), which were immediately flash‐frozen on dry ice. Nasal wash samples were collected as published^37^ by the application of 2 mL of a 0.9% NaCl solution. Samples were stored at –80 °C and shipped on dry ice.

Thawed nasal wash fluid was passed through a polyethersulfone (PES) filter (25‐mm diameter, 0.2‐μm pore size; Millipore, Billerica, MA) supported by a membrane filter (25‐mm diameter, 0.45‐μm pore size; Millipore) on a decontaminated Millipore vacuum manifold. The PES filter was inserted into a Power Soil Lysis tube (Mo Bio Laboratories, Inc., Carlsbad, CA), and DNA was extracted following the manufacturer's recommendations.

Stool samples were aliquoted to 150 mg while still frozen and stabilized as described previously.^38^ Microbial pellets were separated by differential centrifugation and extracted using the Qiagen AllPrep kit (Qiagen, Hilden, Germany), as described previously,^39^ using a robotic system (Tecan Group Ltd., Männedorf. Switzerland). Quality and quantity were assessed using a Labchip GX/GXII touch (PerkinElmer, Inc., Waltham, MA) and a NanoDrop spectrophotometer. Bacterial and human DNA content of extractions from nasal wash fluid and corresponding mock‐extraction controls was quantified by quantitative polymerase chain reaction as described^40^, ^41^ (see Supplementary Information).

### 16S and 18S Ribosomal RNA Gene Amplicon Sequencing and Metagenome Analysis

The V4 regions of the 16S and 18S ribosomal RNA (rRNA) genes were amplified and sequenced as described,^41^ with modifications (see Supplementary Information). Fifty‐seven extraction controls, internal standards, and replicate samples (Supplementary Tables) were used to assess technical variability and remove reagent‐derived contaminant sequences from low‐biomass nasal samples (see Supplementary Information). Amplification and sequencing were performed by the Groupe Interdisciplinaire de Génoprotéomique Appliquée (GIGA; Liège, Belgium). Raw demultiplexed sequencing reads from 16S rRNA gene amplicon sequencing were processed using LotuS (v1.47),^42^ to determine sequence counts per operational taxonomic unit (OTU). For the 18S sequencing data, OTUs were selected and classified as previously described.^43^ Further filtering and aggregation was performed in R (R Foundation for Statistical Computing, Vienna, Austria),^44^ as described,^41^ with modifications (see Supplementary Information).

Whole metagenome shotgun sequencing was performed on libraries prepared from 500 ng of DNA on a HiSeq2500 (Illumina, San Diego, CA) using HiSeq V3 reagents. Metagenomic sequencing was conducted at GATC Biotech AG (Konstanz, Germany). Metagenomic reads were processed and assembled using the metaomic pipeline, IMP,^45^ and genome reconstructions were performed as described.^38^ Functional annotations of genes were assigned as described.^38^, ^46^ For details, see Supplementary Information.

All sequencing data are accessible under NCBI Bioproject PRJNA381395, and genome reconstructions can be browsed through RAST^47^ accessions 6666666.250140 and 6666666.250141.

### Numerical Ecology and Differential Analysis

All numerical analyses of OTU abundances were performed in R (R Foundation for Statistical Computing).^44^ Rarefaction, calculation of rarefied richness, estimation of de facto richness (ACE model), calculation of dispersion indices, and Procrustes analyses were performed using vegan.^48^ Jensen‐Shannon divergences were calculated using phyloseq^49^ and principal coordinate analyses performed using ape.^50^ UniFrac distances were calculated using phyloseq^49^ from trees built^42^ postfiltering using Clustal Omega^51^ and FastTree.^52^ Covariates were identified using permutational multivariate analysis of variance (9,999 permutations), as described,^53^ and potential confounders were assessed using Fisher's exact test and Kruskal‐Wallis' test, as described.^19^ Differential and regression analyses were performed using DESeq2,^54^ with the covariates found to be significant for each microbiome (nasal: abundance ∼ PD/RBD status * gender; gut: abundance ∼ PD/RBD status * diabetes status). Taxa with multiple‐testing adjusted *P* values below 0.05 (and an absolute log_2_ fold change > 1 for differential analyses) were defined as significantly differentially abundant or related to a continuous variable, respectively. Analysis of composition of microbiomes (ANCOM) analysis was performed using the R (R Foundation for Statistical Computing) implementation.^55^ Functional community profiles were predicted based on classified OTU abundances postfiltering using PanFP,^56^ and differential analysis was carried out on sum‐normalized data sets using the Mann‐Whitney U test. All *P* values, except those used for the detection of potential confounders, were adjusted for multiple testing using the Benjamini‐Hochberg (false discovery rate; FDR) method.

## Results

### Study Cohort and Samples

16S rRNA gene amplicon sequencing (to analyze the relative abundances of bacteria and archaea) was successfully performed on 84 flash‐frozen stool samples and 147 nasal wash samples from 76 PD patients (66% male; mean age: 68.0 ± 9.7) and 78 HCs (59% male; mean age: 68.4 ± 6.7) as well as from 21 iRBD subjects (57% male; mean age: 66.1 ± 7.9; *P* value between the groups: 0.05) of the DeNoPa cohort (Table 1 and Supplementary Tables). 18S rRNA gene amplicon sequences (for profiling microeukaryotes) were obtained for 61 of the stool samples (Supplementary Tables).

**Table 1:** Overview of the groups in the study cohort

Data	HCs	PD	iRBD	Significant Differences (*P* Values)
N	78	76	21	—
Age (years)^a^	68.4 ± 6.7	68.0 ± 9.7	66.1 ± 7.9	
Male^a^	59%	66%	57%	
BMI^a^	26.6 ± 4.1	28.5 ± 4.5	26.2 ± 3.4	PD vs. HC 0.006 PD vs. iRBD 0.03
Currently smoking^b^	2	4	1	
PD duration since diagnosis in months^a^	—	72 ± 31	—	—
MDS‐UPDRS total score^a^	8.5 ± 7.8	56 ± 25	18.8 ± 9.4	PD vs. HC < 2.2 × 10^−16^ iRBD vs. HC 1 × 10^−5^ PD vs. iRBD 1 × 10^−9^
MDS‐UPDRS part I score^a^	5.5 ± 4.3	11.7 ± 7.2	11.9 ± 6.0	PD vs. HC 8 × 10^−9^ iRBD vs. HC 8 × 10^−6^
MDS‐UPDRS part III score^a^	1.5 ± 2.8	30 ± 14	3.8 ± 3.2	PD vs. HC < 2.2 × 10^−16^ iRBD vs. HC 9 × 10^−12^ PD vs. iRBD 0.0007
MDS H & Y^a^	0 ± 0	2.14 ± 0.75	0.05 ± 0.21	PD vs. HC < 2.2 × 10^−16^ PD vs. iRBD 2 × 10^−12^
RBD by PSG, N	0	40	21	—
Constipation by Scopa‐AUT Q 5	7	31	11	PD vs. HC 4 × 10^−6^ iRBD vs. HC 4 × 10^−5^
l‐dopa intake^b^	0	66	0	—
Dopamine agonists intake^b^	0	52	0	—
COMT inhibitor intake^b^	0	4	0	—
MAO‐B inhibitors intake^b^	0	52	0	—
Metformin intake^b^	6	7	3	

a Mean ± standard deviation.

b Number of study participants who reported the respective item.

**Figure 1:**
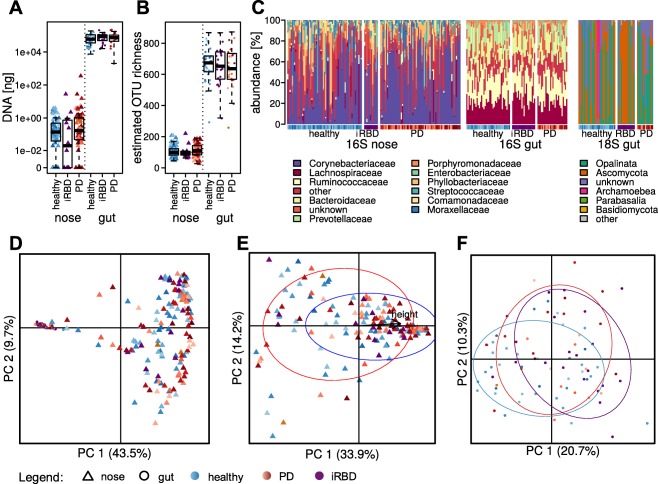
Overview of the observed community structures. (A) DNA yield per mL nasal wash or g of stool. (B) Estimates of OTU richness in nasal and gut samples. (C) The most common bacterial families and eukaryotic divisions or subdivisions in nasal and gut samples. Principal coordinate analyses of Jensen‐Shannon diversities between (D) OTU profiles of nasal and gut samples, (E) prokaryotic family profiles of nasal samples, and (F) OTU profiles of gut samples with circles around 70% confidence intervals for (E) sex (red, female; blue, male) and (F) study group (blue, healthy; red, PD; purple, iRBD). Colors and symbols used for the study participants throughout the articles are represented below. PC, principal coordinate.

The two analyzed body sites exhibited obvious differences in the density of microbial colonization (Fig. 1A), the number of different taxa in the community (Fig. 1B), and community structures (Fig. 1C). At all analyzed taxonomic levels (phylum, class, order, family, genus, and OTU level), variation within the community structures was greater in the nasal samples than in the gastrointestinal samples (multivariate homogeneity of group dispersions *P* value = 0.0001; Fig. 1D). In addition, the nasal data set was sparser, that is, it contained less OTUs common to samples of different individuals, with only a single OTU (of the genus *Corynebacterium*) present in all samples. Paired nasal and gastrointestinal microbiota structures of individuals (n = 56) showed no significant dependence or correlation of both microbiota, indicating that the factors shaping both communities differ.

### Covariates for the Nasal Microbiota in PD and HCs

To find factors with a strong influence on the prokaryotic community structures, we analyzed grouping power by a set of technical factors, such as sampling dates, extraction, and sequencing batches, as well as 70 anthropometric and clinical data points recorded for the DeNoPa cohort (see Supplementary Tables). For the sparse nasal microbiome data set, OTUs were aggregated at the family level. Sex and the related body height (unadjusted *P* values = 0.001 and 0.0012, respectively) of the study participants (n = 147) were found to have the strongest effects (Fig. 1E).

When correcting for sex, eight differentially abundant families were found between PD patients and HCs, mostly with weak significance (*P* values > 0.01; Supplementary Information). To evaluate a possible influence of medication, comparisons were performed between PD patients receiving levodopa, dopamine agonists, catechol‐*O*‐methyl transferase (COMT) inhibitor, and/or monoamine oxidase type B (MAO‐B) inhibitors versus patients who were treatment‐naïve in relation to the respective drugs. One family (Bacillaceae) showed significant differences in patients receiving l‐dopa treatment (FDR‐adjusted *P* value = 0.0004), indicating that its differential abundance may be attributed to treatment with this drug rather than the disease.

### Differentially Abundant Taxa of the Gut Microbiome in PD and Potential Confounders

In the gastrointestinal samples, the study group (PD, HC, and iRBD) represented the best grouping factor for the prokaryotic (unadjusted *P* value = 0.004, explaining 5% of the variation; n = 84; Fig. 1F), but not the eukaryotic, community. Frequency of constipation was not found to be a significant covariate for the prokaryotic gastrointestinal microbiome, although constipation was more common in PD patients and iRBD versus HCs (PD, 62%; iRBD, 75%; HCs, 24%; Fisher's exact test *P* values = 0.0038 and 0.0002), and few taxa with different abundances in constipated versus nonconstipated individuals were found (data not shown). Comorbidity of diabetes and coronary artery disease (each in 5 subjects) were found to affect the prokaryotic taxonomic profiles (unadjusted *P* values = 0.02) and oral intake of diabetes medication was considered as a potential confounder, because the subcohorts contained unequal proportions of diabetes‐affected individuals.

**Figure 2:**
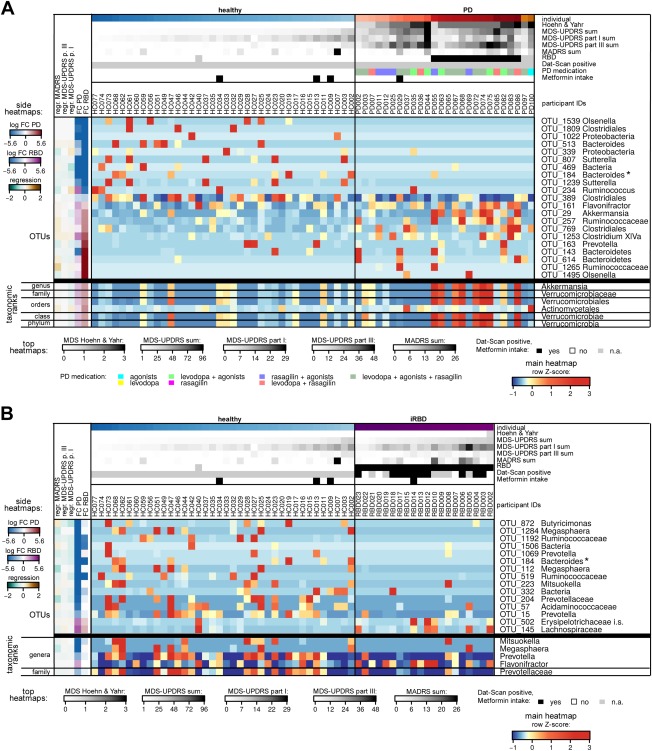
Heatmaps of most differentially abundant taxa in PD patients and individuals with RBD. Relative abundances of prokaryotic OTUs and higher‐level taxa of the gut microbiome that were found to be differentially abundant in (A) PD patients, or (B) iRBD patients or both compared to the HCs (FDR‐adjusted DESeq2 *P* values < 0.001 and/or confirmation by ANCOM). Legends for the cohort‐related indications at the top of the heatmaps, for the summarizing heatmaps to the left, and for the central heatmaps are given to the left and below the heatmaps (for the complete set of differentially abundant taxa, detailed histograms, summary data, and further explanations, see Supplementary Information). For the OTUs, the lowest confident classifications are displayed; i.s.: *incertae sedis*; ^*^the *Bacteroides* OTU_184 was found in both displayed sets. FC, fold change; n.a., not applicable.

Controlling for the intake of oral diabetes medication, 48 OTUs of the gastrointestinal microbiota were found to be differentially abundant in PD patients versus HCs (FDR‐adjusted *P* values < 0.05 and |log_2_ fold change| > 1; n_HC_ = 38, n_PD_ = 26; Fig. 2A; Supplementary Information). Although 14 prokaryotic OTUs in the gastrointestinal microbiota were differentially associated to at least one type of medication, the differentially abundant OTUs between PD patients and HCs were not affected by the medication.

To compare our results with those of earlier studies reporting differentially abundant bacterial families^15^ and phyla^17^ in stool samples from PD patients, differential analyses were repeated with microbial abundances summed up at the taxonomic ranks of genera, families, and phyla. We found differential relative abundances of the genus *Akkermansia*, and along with the enrichment in *Akkermansia*, its parent‐taxa the Verrucomicrobiaceae, the Verrucomicrobiales, and the Verrucomicrobia were differentially abundant.

### Differentially Abundant Taxa in RBD versus HC and Comparison to PD

Differential analysis (with subjects having comorbid diabetes as covariate, like above) identified 41 differentially abundant OTUs in iRBD subjects compared to HCs (FDR‐adjusted *P* values < 0.05 and |log_2_ fold change| > 1; n_HC_ = 38, n_RBD_ = 20; Fig. 2B). Of these 41 OTUs, nine were also among the 48 differentially abundant OTUs in PD patients' gut microbiota compared to HCs and changed in the same direction compared to HCs (Supplementary Information). Thirty other differentially abundant OTUs in RBD versus HC or in PD versus HCs showed the same directions of change in the comparisons to HCs. Therefore, more than 75% of the differentially abundant OTUs in the comparisons of PD or iRBD versus HCs showed qualitatively similar changes compared to HCs. For example, common patterns in PD and iRBD were observed for *Anaerotruncus* spp., *Clostridium* XIVb, several Bacteroidetes, and an unclassified bacterium OTU_469.

Two of the OTUs that were significantly higher abundant in PD compared to HCs (*Akkermansia* sp. and *Prevotella* sp.) were also significantly more highly abundant in PD patients with RBD by PSG compared to PD patients without RBD, despite the fact that these OTUs were not significantly differential in subjects with iRBD compared to PD.

### The Gut Microbiome in Relation to Motor and Nonmotor Phenotypes of PD

The relative abundances of three of the differentially abundant gastrointestinal OTUs in PD were significantly related to motor symptoms (by MDS‐UPDRS part III) within the PD cohort, namely OTUs of *Anaerotruncus* spp., *Clostridium* XIVa, and Lachnospiraceae (Fig. 2B). Six OTUs, including from the genera *Anaerotruncus, Akkermansia*, and several unclassified Bacteria, were significantly related to nonmotor symptoms according to MDS‐UPDRS part I in the gut microbiota of PD patients (Fig. 2B). More specifically, the presence of depression (as evaluated by the MADRS) and anxiety (by MDS‐UPDRS I.4) were significant grouping factors independently of the study group for the prokaryotic (both unadjusted *P* values = 0.02), but not the eukaryotic, microbiota and differed significantly in the PD and iRBD patients versus HCs. OTUs that were significantly differentially abundant and related to depression in the PD cohort included *Anaerotruncus* spp. No effect of cognition (by MMSE and/or MoCa), sleepiness (ESS), or pain (MDS‐UPDRS I.9) on the gastrointestinal microbial community structures was observed.

### Whole Metagenome Sequencing of Selected Samples to Describe Novel Genomes of Differentially Abundant Taxa

**Figure 3:**
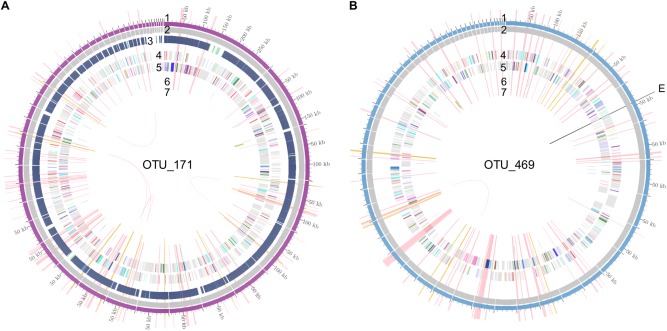
Overviews of genomic reconstructions of two novel microbial populations depleted in patients with PD. (A) OTU_171, classified as Melainabacterium, (B) OTU_469, classified as alpha‐proteobacterium. 1) Contiguous sequences with lengths, 2) metagenomic depth of coverage, 3) % similarity to MelB1,^57^ 4 and 5) predicted proteins colored according to functional categories, 6 and 7) tRNA and rRNA loci, and E) endoglucanase with synuclein‐like domain; pink spokes highlight unique essential genes, golden spokes highlight phylogenetic marker genes. kb = kilobase; tRNA, transfer RNA.

Because we observed 10 OTUs without taxonomic classification with significantly different abundances in the patient cohorts versus HCs, we aimed to characterize their genomes and their functional potential. Only three of these differentially abundant OTUs were found at relative abundances above 1% (OTU_171, OTU_469, and OTU_332), potentially allowing recovery of the genomes by whole metagenome shotgun sequencing. Metagenomic sequencing data (20 Gb) were obtained from two samples (of an HC and an iRBD subject) with high levels of two of these OTUs. One reconstructed genome each was linked to OTU_469 and OTU_171 using the 16S genes predicted in the metagenomic data (Fig. 3; see Supplementary Information for the method). OTU_469 was classified as an α‐proteobacterium. Assessment of the functions encoded in the genome reconstruction pointed to a fermentatitve lifestyle, auxotrophy for most vitamins, and motility. Interestingly, the genome encoded an endoglucanase with a synuclein‐like domain. OTU_171 was found to have 98.9% identity to a genome reconstruction of the cyanobacterium Melainabacterium MelB1,^57^ and these two shared traits such as a fermentative metabolism, flagellation, and B vitamin synthesis. No cyanobacterial toxin genes^58^, ^59^, ^60^ were found (see Supplementary Tables).

## Discussion

In this study, the community structures of nasal and gut microbiomes of 76 PD patients and 78 matched healthy individuals were analyzed and compared with 21 subjects with iRBD, the most specific prodrome of PD.^25^ Specific effort was taken to preserve the microbiota by flash‐freezing of nasal fluid and stool, to avoid any technical confounders.^39^, ^61^ This approach was chosen because the nasal cavity and gastrointestinal tract have been suggested to constitute the two ports of entry for a possible pathogenic agent and from where PD‐related aggregated aSyn spreads to the CNS. So far, microbiome‐based analyses in relation to PD have focused either on the gastrointestinal tract^15^, ^16^, ^17^, ^19^ or not systematically compared both sites.^15^, ^23^

OTUs common to the nasal and gastrointestinal microbiota were found, likely attributed to nasal discharge being swallowed.^62^ However, none of these OTUs were found to differ significantly between PD patients and HCs, indicating that distinct signatures of PD are potentially present in the gut and nasal microbiota. The nasal microbiota displayed higher variation over the different individuals and most OTUs were only sparsely detected, in accord with earlier observations.^23^, ^63^, ^64^, ^65^ The strongest grouping factor observed for the nasal microbial community structure was sex. Controlling for this, only few differentially abundant bacterial families were observed in PD patients and most of them were rare. More bacterial families were found to be potentially affected by PD medication than to be different in relation to PD. Therefore, our results, in accord with other recent findings,^23^ indicate that the nasal microbiome may be relatively uninformative as a source of biomarkers for PD.

In contrast to the results obtained for the nasal cavity, we found significant grouping of the gastrointestinal microbial community structures according to the three study groups (PD patients, iRBD subjects, and HCs). The same analysis revealed influences of medication of oral antidiabetics, which has previously been observed.^66^ Controlling for this factor in the differential analysis, we confirmed one previous observation^16^, ^17^, ^19^, ^20^ of differences in microbial abundances in PD, namely the enrichment of *Akkermansia* sp. in individuals with PD. Enrichment in *Akkermansia muciniphila* is known to be related to longer gastrointestinal passage times.^67^, ^68^ However, we did not find *Akkermansia* abundances to be related to self‐reported constipation. *Akkermansia* spp. is usually considered to be beneficial for human physiology,^69^ attributed to strengthening of gastrointestinal barrier function and anti‐inflammatory immune stimulation,^70^, ^71^, ^72^ but depletion of the mucus layer by enriched *Akkermansia* sp. has been linked to higher susceptibility to a pathogen in an animal model.^73^ Butyrate‐producing bacteria have also been associated with opposing effects in PD, including a protective role attributed to the observed decrease in PD patients^16^ and a causative role attributed to the induction of microglia activation and motor symptoms through short‐chain fatty acids in a mouse model.^74^ In the present study, no depletion of butyrate producers was observed, and predictions of the coding potential of the microbiota based on the classified OTUs indicated no significant differences in any butyrate‐producing enzymes (see Supplementary Information). These differences to earlier studies^16^, ^17^, ^19^ indicate the necessity of further studies with direct measurements of short‐chain fatty acid levels in stool^17^ or in situ as well as in larger cohorts. Such studies would also enable the identification of microbiota associated with nonmotor phenotypes.^75^ Our results, in line with observations in human cohorts^76^, ^77^ and animal models,^78^, ^79^ point to a relationship between the microbiome and depression, independently of antidepressive medication.

An overlap of differential taxa was observed in iRBD and PD compared to HCs. However, the gastrointestinal microbial signatures of RBD were distinct from those in PD, showing, among others, a decrease in Prevotellaceae, similar to observations in a recent study with newly diagnosed PD patients.^20^ Longitudinal observations of RBD patients who are likely to progress to an aSyn aggregation disorder will be important to characterize the changes afflicted before and close to the onset of motor disease and to validate the predictive potential of these changes for the risk of developing PD.

Several recent studies have relied on predictions of functional consequences from microbial abundances,^16^, ^19^ but elucidation of the actual coding potential of the microbial communities^20^ is indispensable, because the predictions do not account for microbes without sequenced genomes, which make up approximately 40% of the gut microbiome.^80^ One of the novel OTUs (OTU_171) that we observed to be depleted in PD patients was from the family of Melainabacteria^57^ of the phylum Cyanobacteria. Free‐living cyanobacteria are known to produce a range of neurotoxins, such as Anatoxin and Saxitoxin and the neurotoxin β‐N‐methylamino‐l‐alanine (BMAA),^81^ which has been linked to neurodegenerative diseases.^82^, ^83^, ^84^ Although the enzymes required for the biosynthesis of BMAA are not yet known,^85^ no known enzymes for the production of neurotoxins are encoded in the presented genome reconstruction. This observation, together with the depletion in individuals with PD, argues against a role of the OTU as a producer of neurotoxins. This particular strain may, in fact, have a protective role, potentially outcompeting related toxin‐producing strains from the same species as widely observed for enteric pathogens.^86^ Another noteworthy group of proteins are those with similarity to aSyn, of which we found a gene in the metagenomic reconstruction of the so far undescribed OTU_469 that was depleted in PD and iRBD patients and may therefore also exert a protective effect.

In summary, our analysis revealed and confirmed differential abundances of gut, but not nasal, microbial taxa in PD patients and also revealed overlaps between PD and iRBD microbiota. We point out that standardized sample collection and preservation, whole metagenomic analyses in combination with functional omics, and metabolite detection are necessary to pinpoint microbial functions that may interact with the human body during the development of PD and to derive concrete hypotheses about how the microbiome may be involved in the different dimensions of this heterogeneous disease.

## Acknowledgments

The authors thank Latifa Karim and Wouter Coppieters (Groupe Interdisciplinaire de Génoprotéomique Appliquée, Liège, Belgium) for excellent technical assistance and advice. In silico analysis results presented in this article were obtained using the high‐performance computing facilities of the University of Luxembourg and the administrators are thanked for their excellent support. Janine Habier and Daniel Kay (LCSB) are thanked for provision of *Salmonella* DNA and yeast cells, respectively. Olivia Steuer (Paracelsus‐Elena‐Klinik, Kassel) and Matthias Giese (Philipps‐University Marburg) are acknowledged for help with collection of samples and Elisabeth Lang (Paracelsus‐Elena‐Klinik, Kassel) with clinical data management. Laura Lebrun (LCSB) is thanked for assistance with the extraction platform. Anne Kaysen and Shaman Narayanasamy (LCSB) are acknowledged for discussion and advice on the metagenomic analysis. Rudi Balling and Regina Becker (LCSB) are thanked for support and discussion of the project.

## Author Roles

(1) Research Project: A. Conception and Design; B. Acquisition of Data; C. Analysis and Interpretation of Data; (2) Manuscript: A. Writing of the First Draft, B. Review and Critique; (3) Other: A. Statistical Analysis; B. Obtaining Funding; C. Technical Support; D. Supervision of Data Collection.

A.H.‐B.: 1A, 1C, 2A, 2B

U.B.: 1C, 2B

T.W.: 1B, 2B

F.S.‐D.: 1B, 2B

A.J.: 1B, 2B

E.S.‐W.: 1B, 2B

C.T.: 1B, 2B

W.H.O.: 1A, 2A, 2B, 3A

B.M.: 1A, 1B, 2A, 2B, 3A, 3D

P.W.: 1A, 2A, 2B, 3A

## Financial Disclosures

F.S.‐D. has received honoraria for speaking engagements by AbbVie, Desitin, Licher MT, Medtronic, Orion Pharma, UCB, and Zambon. Congress participation was sponsored by Licher MT, TEVA, AbbVie and MundiPharm. C.T. has received personal fees for advisory boards from: Gruenenthal, UCB, Vifor Pharma, Britannia Pharmaceuticals, and Benevolent; payments for lectures: UCB, Britannia, Mundipharma, Abbvie, and Servier; and royalties from Schattauer Verlag, licensing fees PDSS‐2. W.H.O. has received independent research grants from Novartis Pharma Germany and honoraria for consultancy from AbbVie, Bristol‐Myers‐Squibb, Mundipharma, Novartis Pharma, Roche and UCB‐Pharma, and for presentations from AbbVie, Desitin, Novartis Pharma, and Prothena, and is consultant to Adamas and Novartis. W.H.O. is a member of advisory boards of Adamas, BristolMyerSquibb, Eisai, GEHealth, Mundipharma, Novartis, Roche, UCB, and the Executive Board of the European Brain Council, and has been treasurer of the European RLS Study group and speaker of the German Parkinson Study group. W.H.O. is Hertie‐Senior‐Research Professor supported by the Charitable Hertie‐Foundation, Frankfurt/Main, Germany. W.H.O. has received grants from the German Ministry of Education and Research, the German Research Foundation, European Union, Parkinson Fonds Deutschland, and the Michael J. Fox Foundation for Parkinson's Research. W.H.O. holds shares from BiogenIdec, Medigene, Merck, and Roche. B.M. has received independent research grants from TEVA‐Pharma, Desitin, Boehringer Ingelheim, and GE Healthcare and honoraria for consultancy from Bayer Schering Pharma AG, Roche, AbbVie, TEVA‐Pharma, and Biogen and for presentations from GlaxoSmithKline, Orion Pharma, and TEVA‐Pharma and travel costs from TEVA‐Pharma. B.M. is a member of the executive steering committee of the Parkinson Progression Marker Initiative and the Systemic Synuclein Sampling Study of the Michael J. Fox Foundation for Parkinson's Research and has received grants from the German Ministry of Education and Research, European Union, Parkinson Fonds Deutschland, Deutsche Parkinson Vereinigung, the Michael J. Fox Foundation for Parkinson's Research, and Stifterverband für die deutsche Wissenschaft, and has scientific collaborations with Roche, Bristol‐Myers Squibb, Ely Lilly, Covance, and Biogen. P.W. has received grants from the Luxembourg National Research Fund, the Luxembourg Ministry of Higher Education and Research, and the European Union, and has scientific collaborations with Qiagen, and has received reviewer's allowance from the Novo Nordisk Foundation Challenge Programme, Sweden.

## Supporting information

Additional Supporting Information may be found in the online version of this article at the publisher's web‐site.

Supplementary Information

**Supplementary TABLE 1**. Cohort characteristics; technical details relating to the sequenced patient samples and the control samples.
**Supplementary TABLE 2**. Numbers of reads per sample and OTU, genus, family, order, class, or phylum for 16S rRNA gene amplicon sequencing data; numbers of reads per sample and OTU or division for 18S rRNA gene amplicon sequencing data; estimated relative abundances of KEGG (Kyoto Encyclopedia of Genes and Genomes) orthologous groups per sample; results of differential analysis of predicted KEGG orthologous groups in PD versus HC and iRBD versus HC; gene functions in the genome reconstructions of OTU_171 and OTU_469; and results of search for cyanobacterial toxin genes in the genome of OTU_171.
**Supplementary Information**: Supplementary methods; explanation for Figure 2, detailed plots, numerical summaries, and statistics of the differential analyses; supplementary references.
