# Supplementary material for: The nasal and gut microbiome in Parkinson's disease and idiopathic rapid eye movement sleep behavior disorder
Source: Mov Disord. 2017 Aug 26;33(1):88–98. doi: 10.1002/mds.27105 (PMC5811909; doi:10.1002/mds.27105)
Supplement: Supplementary file 1 — Supplementary Information [file MDS-33-88-s001.pdf]

Supplementary Information:

The nasal and gut microbiome in Parkinson's disease and idiopathic REM sleep behavior disorder

Anna Heintz-Buschart, PhD,[1] Urvashi Pandey, MS,[1] Tamara Wicke,[2,3] Friederike Sixel-Döring, MD,[2,3] Annette Janzen, MD,[3] Elisabeth Sittig-Wiegand SN,[3] Claudia Trenkwalder, MD,[2,4] Wolfgang H Oertel, MD,[3] Brit Mollenhauer, MD, [2,5,$] Paul Wilmes, PhD[1$]*

1 Eco-Systems Biology Research Group, Luxembourg Centre for Systems Biomedicine (LCSB), University of Luxembourg, Luxembourg.

2 Paracelsus-Elena-Klinik, Kassel, Germany.

3 Department of Neurology, Philipps University Marburg, Germany.

4 University Medical Center Goettingen, Department of Neurosurgery, Goettingen, Germany.

5 University Medical Center Goettingen, Department of Neurology, Goettingen, Germany.

$: equal contributions

*corresponding authors:

Dr Paul Wilmes, Eco-Systems Biology Research Group, Luxembourg Centre for Systems Biomedicine, 6, avenue des Hauts-Fourneaux, 4362 Esch-sur-Alzette, Luxembourg; +352-466644-6188; paul.wilmes@uni.lu

Dr Brit Mollenhauer, Paracelsus-Elena-Klinik, Klinikstraße 16, 34128 Kassel, Germany; +49-561-6009-200; brit.mollenhauer@paracelsus-kliniken.de

Supplementary methods

qPCR quantification of bacterial and human DNA

DNA extracted from nasal wash fluid and corresponding mock-extraction controls was amplified in duplicates, using primers targeting prokaryotes (F_Bact1369 and R_Prok1492)[1,2] and human phenylalanine-tRNA-synthase (PHA_Syn5-FWD_AAGCCCAACGATGATCAAAT; PHA_Syn5-REV_CAGGGGACTGTCATACACCA) using iQ SYBR Green Supermix (Bio-Rad Laboratories) on a LightCycler 480 (Roche Diagnostics, Germany). Absolute DNA copy numbers were calculated using standards of genomic DNA from *Salmonella typhimurium* LT2 and human cells (Caco-2) as described.[1,2]

16S and 18S rRNA gene amplicon sequencing

The V4 region of the 16S rRNA gene was amplified by using primers 515F[3] and 805R.[4] For the nasal wash-derived DNA and control samples, 30 amplification cycles were employed instead of 25 (applied for the stool-derived DNA) and the amplicon copy number was determined by qPCR and isomolecular mixtures of the libraries were prepared for the sequencing runs. The V4 region of the 18S rRNA gene was amplified from the DNA extracted from the stool samples only, by using primers 574*F and 1132R targeting microeukaryotes.[5] Sequencing with 2 x 300 nt was performed using the V3 MiSeq kit on a MiSeq platform (Illumina). Amplification and sequencing was performed by the Groupe Interdisciplinaire de Génoprotéomique Appliquée (GIGA; Liège, Belgium). All sequencing data is accessible under NCBI Bioproject PRJNA381395.

Amplicon sequence data processing

Raw de-multiplexed sequencing reads from 16S rRNA gene amplicon sequencing were processed using LotuS v1.47,[6] including taxonomic assignment using the Ribosomal Database Project (RDP) classifier. Further analysis and filtering was performed in R.[7] Operational taxonomic units (OTUs) with reference sequences which could not be assigned at the domain level with a confidence > 0.8 were discarded. In addition to the stool and nasal wash samples, 57 extraction controls, internal standards and replicate samples were analyzed. OTUs that were uncommon (represented by less than 10 reads in all samples) or attributable (by the RDP classifier or blastn) to genomes of non-target organisms (such as human, mitochondria, plants and chloroplast) or reagent contaminants were removed. To detect sequences likely originating from reagent contaminants, sequencing controls without input ("no-template") were first analyzed. A single OTU (genus *Alcaligenes*) present in all no-template-controls was removed from the dataset. In a second step, OTUs over-represented in the no-template controls (defined by being represented by more than 10 times the relative number of reads in the no-template datasets compared to the average sample-derived datasets from the same sequencing batch) were removed from the whole dataset. In a third step, OTUs over-represented in any of the mock-extraction controls were removed from the whole dataset. Replicate datasets from the same nasal wash samples were added up for further analysis, while for replicate datasets of the stool samples, only the replicate with the highest number of reads was retained. Good's coverage estimates were used to determine the numbers of reads required to represent 99 % of the communities in all nasal wash and stool samples (1,711 and 3,527 respectively). Reads were aggregated at higher taxonomic levels for confidently assigned OTUs.

Raw de-multiplexed sequencing reads from 18S rRNA gene amplicon sequencing were processed as described earlier,[8] including taxonomic assignment using the PR2 database.[9] OTUs classified as Metazoa or Streptophyta were removed. OTUs that could not be classified confidently were blasted against the NCBI-NR database. OTUs with significant alignments to mammalian, plant or bacteria genomes were manually removed. Rare OTUs and those likely stemming from a sequencing contaminant were removed analogous to the description above. 15 samples that did not meet the number of reads required to represent 99 % of the community based on Good's coverage estimates (4,414) were likewise removed from the final dataset. Samples of 9 individuals were withheld from the statistical analysis because they did not meet inclusion criteria for PD or RBD.

Metagenomic sequencing and analysis

Whole metagenome shotgun sequencing was performed on libraries prepared from 500 ng DNA on a HiSeq2500 (Illumina) using HiSeq V3 reagents. Metagenomic sequencing was conducted at GATC Biotech AG (Germany).

Metagenomic reads were trimmed, human reads were removed and preprocessed reads were assembled using the integrated metaomic pipeline IMP.[10] The sequencing data is accessible under NCBI Bioproject PRJNA381395. Functional annotations of the genes were achieved using HMMer3.0[11] and hidden Markov models from an in-house collections[12] and for Curli genes.[13] rRNA genes were predicted using Barrnap. Depths of coverage of contigs were calculated within IMP. Contigs were binned according to pentamer frequencies and depth of coverage as described previously.[12] One bin was manually refined based on depth of coverage.

Linking of metagenomic genome reconstructions to 16S rRNA amplicon-based OTUs, published taxonomies and metagenomic species

Metagenomic 16S sequences were clustered with OTU-representative amplicon sequences using CD-HIT-EST-2D.[14] In addition, metagenomic reads were mapped against OTU-representative amplicon sequences using Bowtie2[15] and contigs mapped by the other partner of a read mapping to an OTU sequence were extracted using picard. Further taxonomic information of reconstructed genomes were obtained by calling phylogenetic marker genes using fetchMG[16,17] and the mOTU taxonomy[18] as described previously.[12] For bins associated with metagenomic linkage groups without a classification at the phylum level, published metagenomic species [19] with close similarity to the bins of unknown taxonomy were searched using the NCBI's blastp web application. Further functional analyses were performed in RAST,[20] as described[21] including comparing one reconstructed genome to the published MelB1 genome[22] and searching genes with similarity to Anatoxin[23,24] or Saxitoxin[25] biosynthesis genes (genome accessions 6666666.250140 and 6666666.250141).

Results of the differential analysis of the nasal microbiota

Summarized results of the differential analyses of the nasal microbiota at the family level.

taxon	HC mean % abundance	PD vs HC log$_2$ fold change	PD vs HC adj. P value	iRBD vs HC log$_2$ fold change	iRBD vs HC adj. P value
Listeriaceae	0.03	-5.72	0.046	-7.41	0.443
Bacillaceae 1	0.99	-2.94	0.022	-1.68	0.959
Ruminococcaceae	0.73	-2.68	0.031		
Micrococcaceae	1.03	-2.62	0.031	-0.85	0.959
Carnobacteriaceae	0.16	2.06	0.046	-2.60	0.794
Hydrogenophilaceae	0.01	4.34	0.046		
Hyphomicrobiaceae	0.07	6.95	**5.86E-05**		
Desulfovibrionaceae	0.04	-7.46	0.046	-23.30	0.001
Bacillales *incertae sedis* XII	0.29	-3.45	0.415	-21.46	0.001
Oxalobacteraceae	0.02	-1.29	0.516	-8.54	0.045

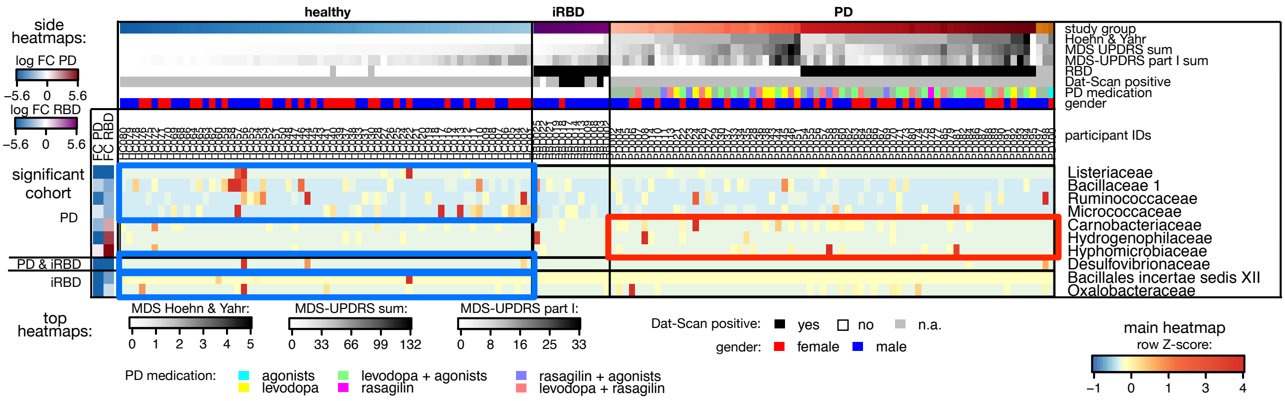

Heatmap: Relative abundances of bacterial families of the nasal microbiota that were found to be differentially abundant in either PD patients or iRBD patients or both compared to the healthy controls (FDR-adjusted P-values < 0.05). Legends for the cohort-related indications at the top, the summarizing heatmaps to the left and for the central heatmap are given to the left and below the heatmap.

Results of the differential analysis of the gut microbiota

Explanation of heatmaps with differentially abundant taxa, eg. Figure 2A:

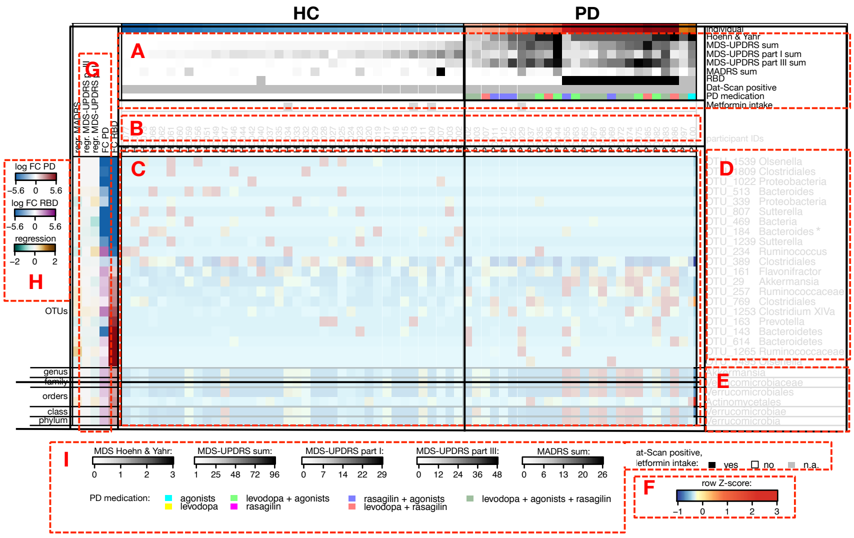

A. Color-coded representation of subject information: study group, staging, motor and non-motor symptoms, medication etc. (legends in I).
B. Subject identifiers.
C. Heatmap of microbial abundances. Each field represents the abundance of one microbial taxon in a given study subject's microbiome compared to the abundance of this taxon in all analyzed samples (color bar in F). The taxa were selected based on their differential abundance in PD patients compared to healthy controls (different taxonomic levels are delineated by a black line).
D. Names of differentially abundant operational taxonomic units (OTUs) and the lowest taxonomic classification of each OTU.
E. Names of differentially abundant taxa at genus, family, order, class or phylum level.
F. Color bar legend for the heatmap (C). Abundances are represented relative to the mean and standard deviation of the abundances of each taxon in the analyzed samples (Z-score of 1 is one standard deviation away from the mean). Taxa with a higher than average representation in an individual's microbiome are labeled in red, taxa with a lower than average representation are labeled in blue.
G. Heatmaps representing summarized measures of the taxon abundances. From right to left: $\log_2$ fold change between PD patients and healthy controls, $\log_2$ fold change between iRBD patients and healthy controls, unit by unit regression of taxon abundance to non-motor symptoms (MDS-UPDRS I), motor symptoms (MDS-UPDRS III) and depression (MADRS) in the PD patients (color bars in H).
H. Color bar legends of the heatmaps in G.
I. Color bars and legends for subject-related information represented in A.

Example (OTU_749, of genus *Anaerotruncus*):

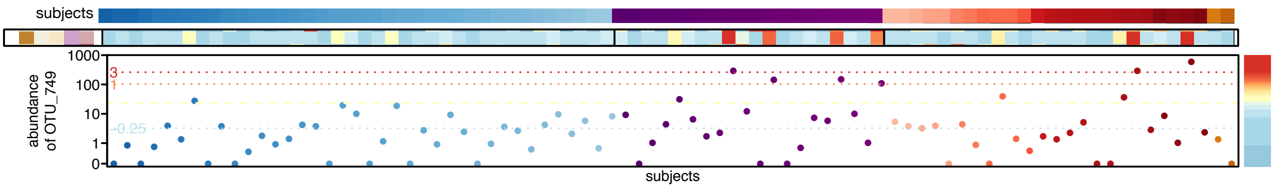

Visualizations of the distributions of the relative abundances of the differential taxa

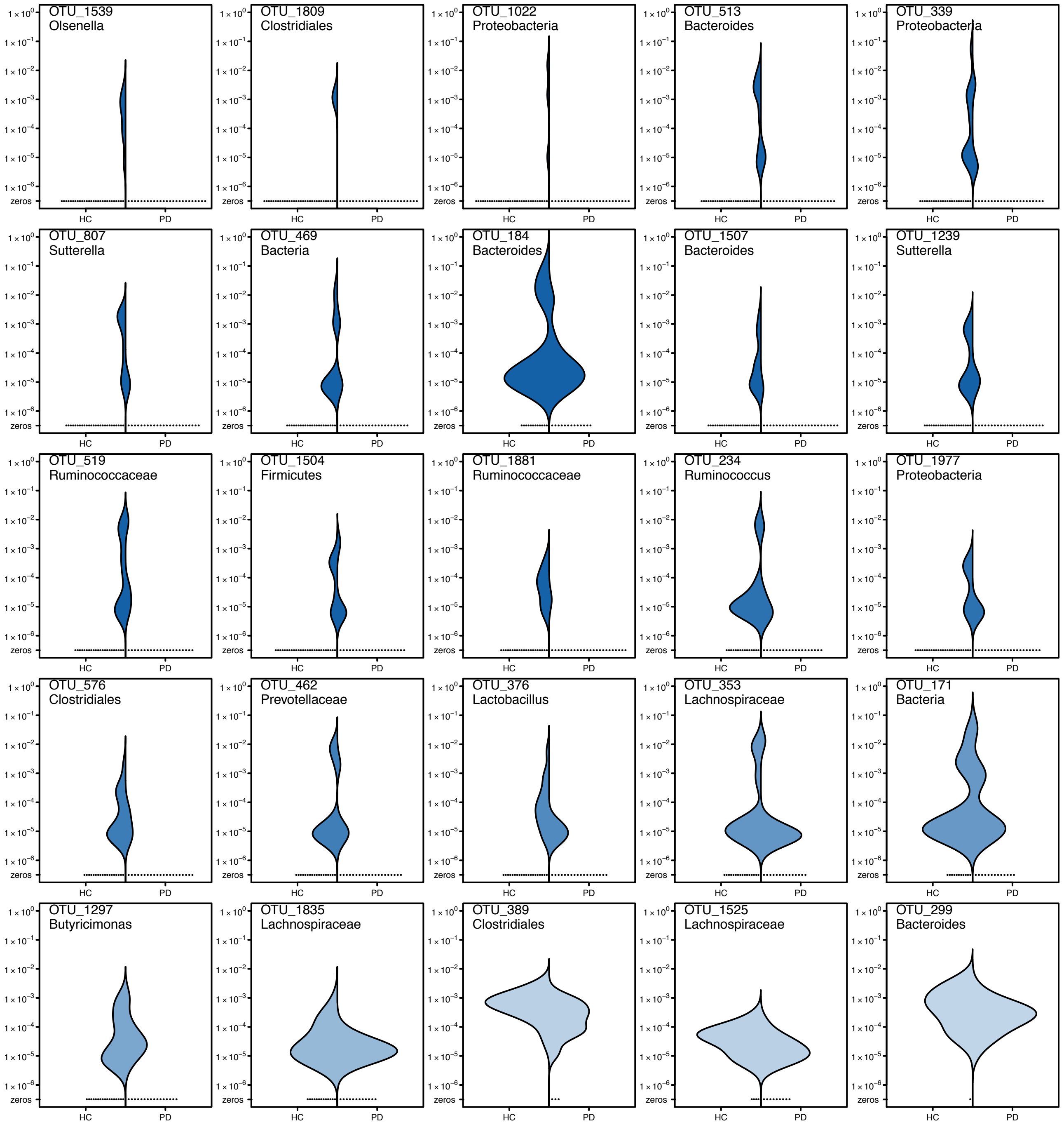

Histograms (part 1): Distributions of the relative abundances of bacterial OTUs in the gut microbiota that were found to be differentially abundant in PD patients compared to the healthy controls (FDR-adjusted P-values < 0.05). Relative abundances are estimated from sum-normalized rarefied data; only values for individuals without type 2 diabetes are shown; left: HC, right: PD.

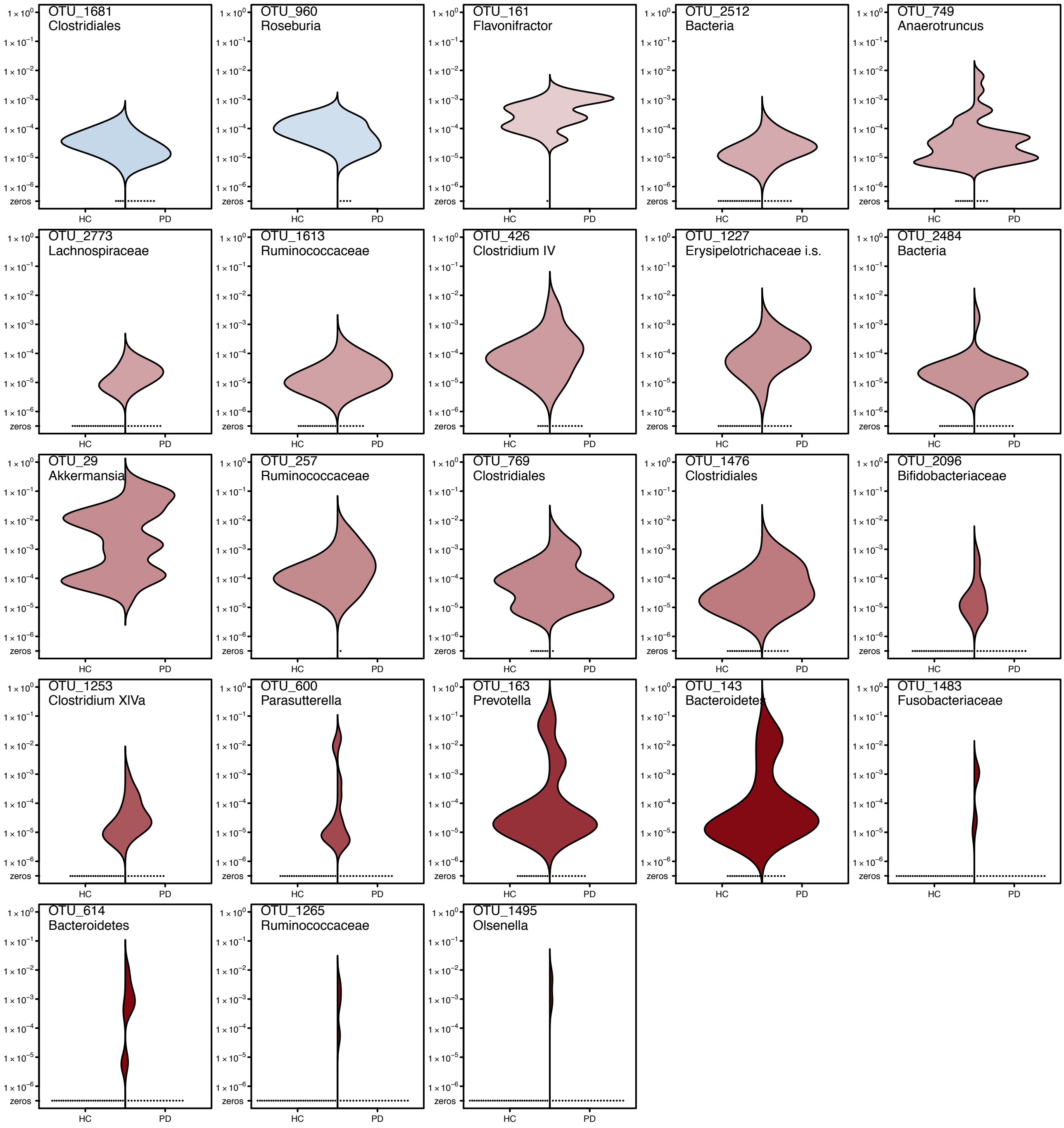

Histograms (part 2): Distributions of the relative abundances of bacterial OTUs in the gut microbiota that were found to be differentially abundant in PD patients compared to the healthy controls (FDR-adjusted P-values < 0.05). Relative abundances are estimated from sum-normalized rarefied data; only values for individuals without type 2 diabetes are shown; left: HC, right: PD.

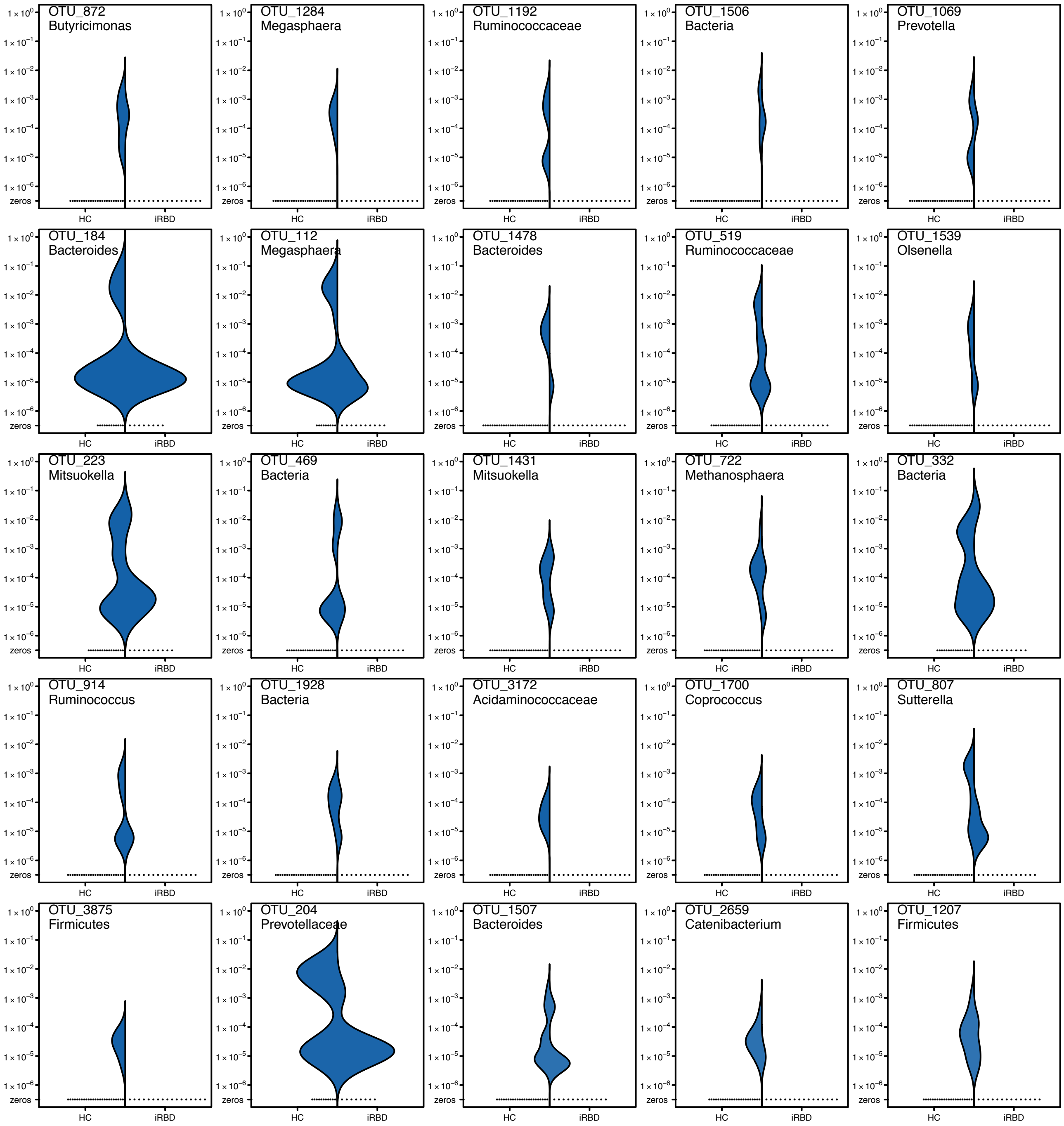

Histograms (part 3): Distributions of the relative abundances of bacterial OTUs in the gut microbiota that were found to be differentially abundant in individuals with iRBD compared to the healthy controls (FDR-adjusted P-values < 0.05). Relative abundances are estimated from sum-normalized rarefied data; only values for individuals without type 2 diabetes are shown; left: HC, right: iRBD.

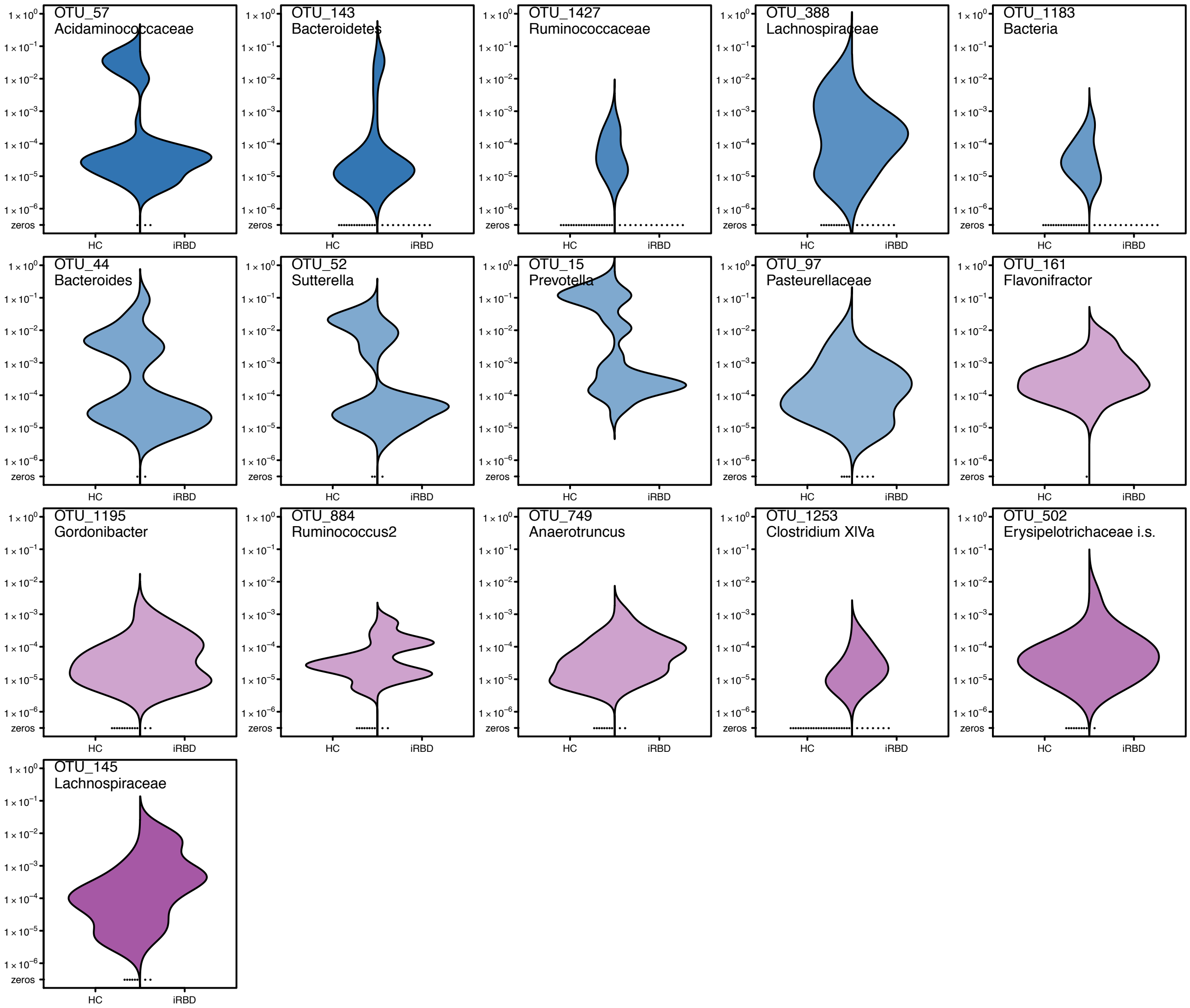

Histograms (part 4): Distributions of the relative abundances of bacterial OTUs in the gut microbiota that were found to be differentially abundant in individuals with iRBD compared to the healthy controls (FDR-adjusted P-values < 0.05). Relative abundances are estimated from sum-normalized rarefied data; only values for individuals without type 2 diabetes are shown; left: HC, right: iRBD.

Overview of the relative abundances of all differentially abundant taxa

The following heatmaps facilitate the assessment of the detected differences from the point of view of varying normalization strategies.

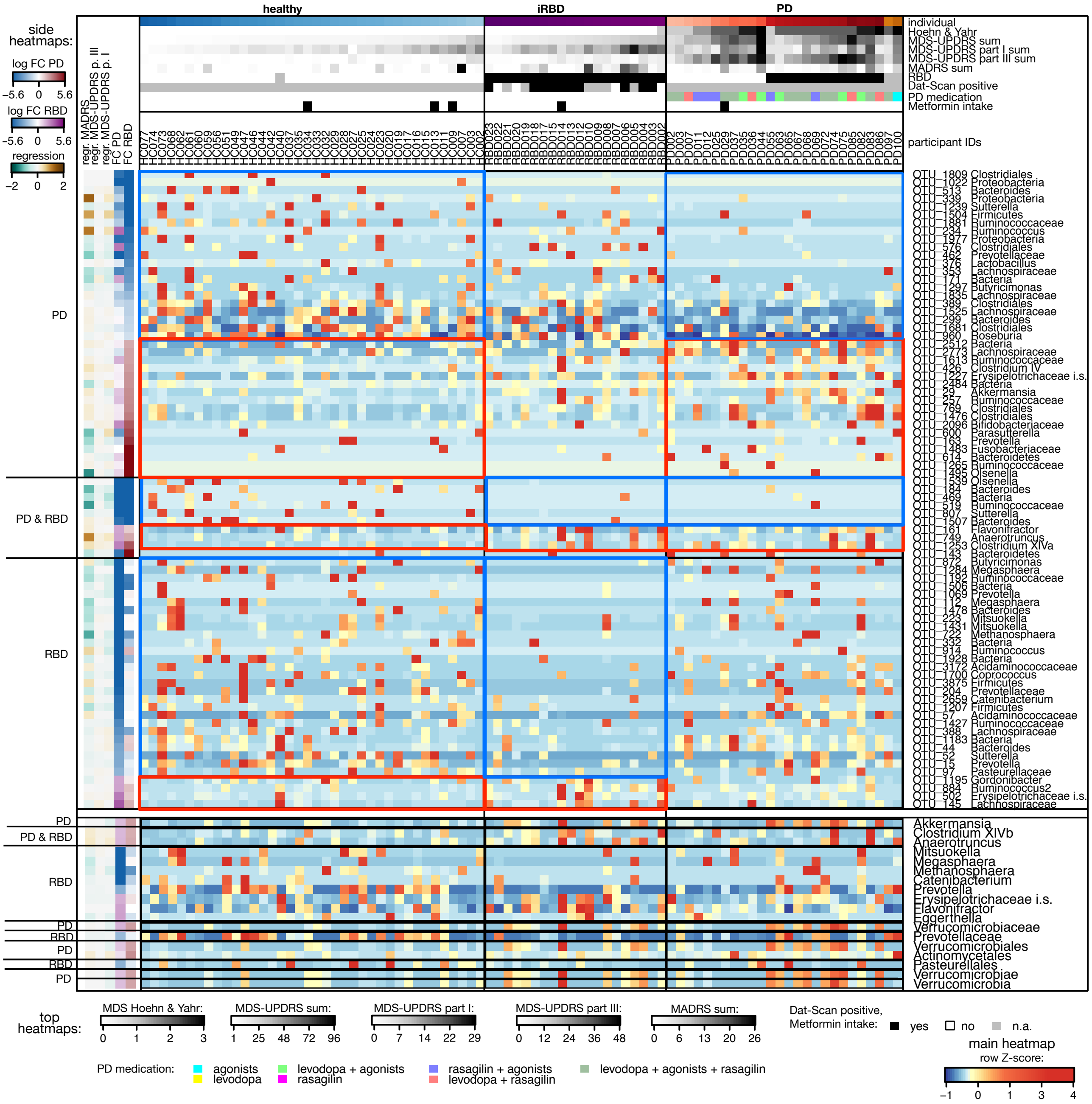

Heatmaps of differentially abundant (absolute $\log_2$ fold change > 1 and P-value < 0.05) taxa in PD patients and individuals with iRBD. Relative abundances (based on DESeq2-normalization) of prokaryotic OTUs and higher-level taxa of the gut microbiome that were found to be differentially abundant in either PD patients, or iRBD patients or both compared to the healthy controls.

Overview of relative abundances of all differentially abundant taxa, sum normalization

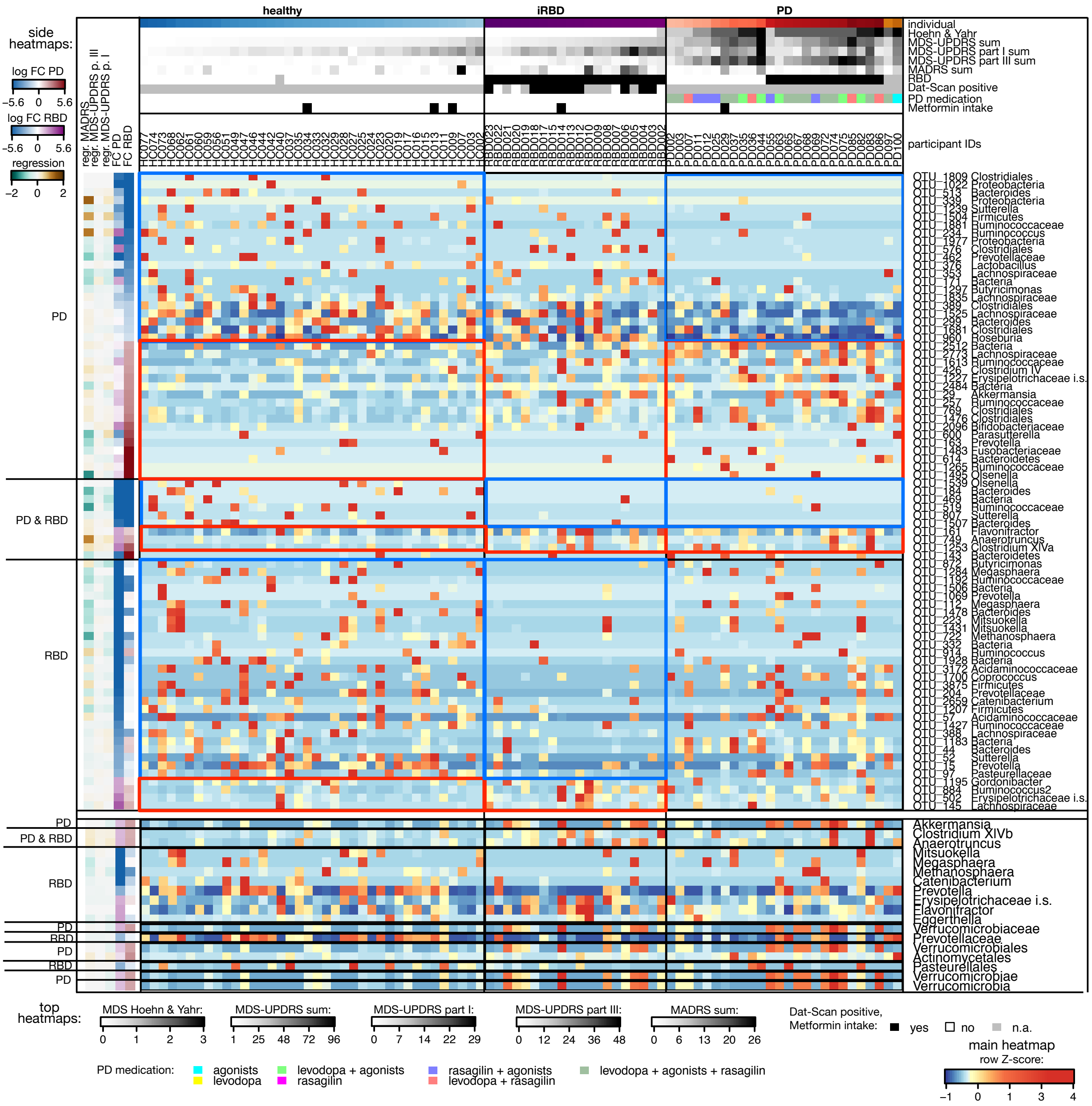

Heatmaps of differentially abundant (absolute $\log_2$ fold change > 1 and P-value < 0.05) taxa in PD patients and individuals with iRBD. Relative abundances (after sum normalization of all reads) of prokaryotic OTUs and higher-level taxa of the gut microbiome that were found to be differentially abundant in either PD patients, or iRBD patients or both compared to the healthy controls.

Overview of relative abundances of all differentially abundant taxa, sum normalization after rarefaction

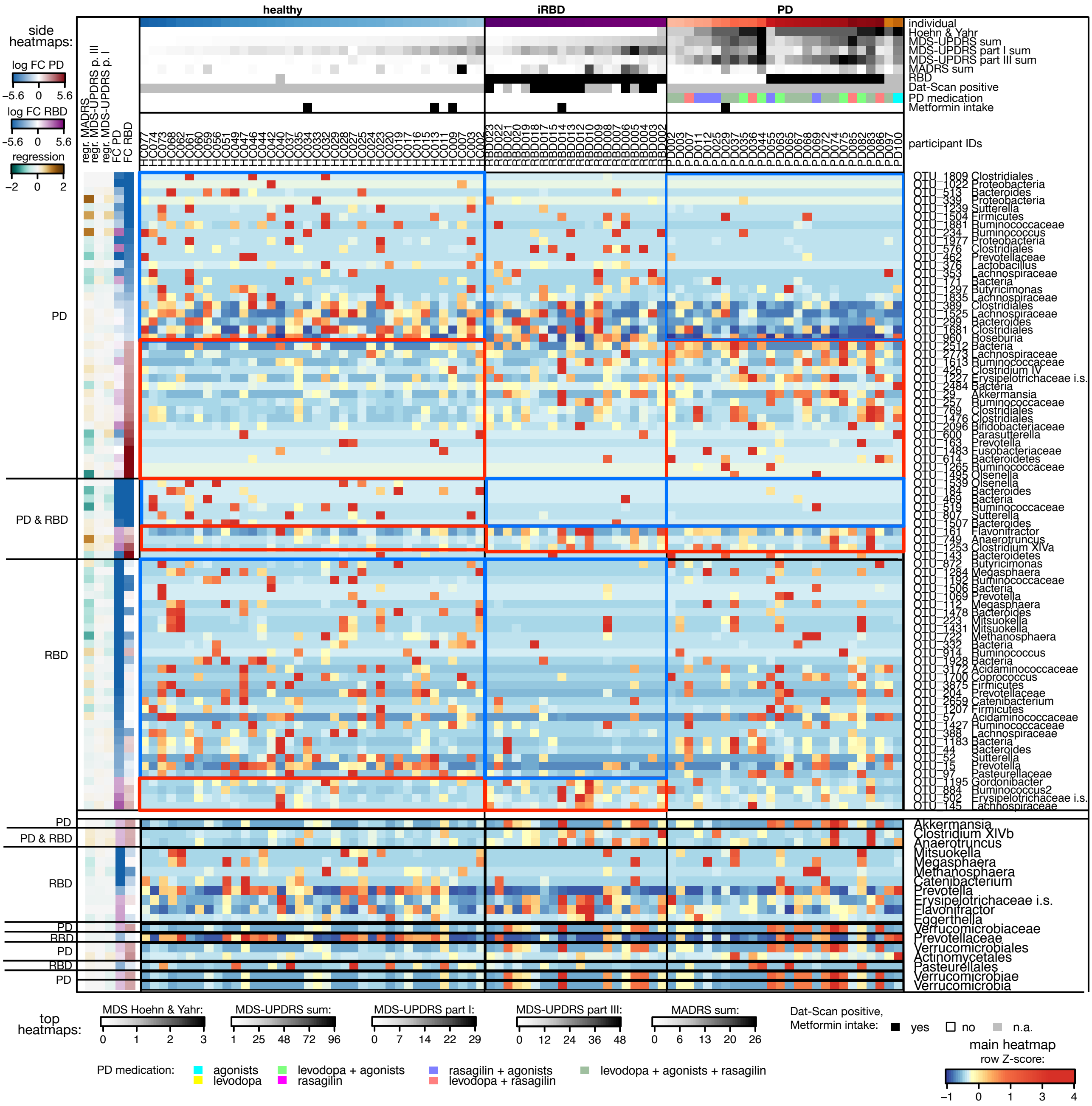

Heatmaps of differentially abundant (absolute $\log_2$ fold change > 1 and P-value < 0.05) taxa in PD patients and individuals with iRBD. Relative abundances (after sum normalization of all rarefied datasets) of prokaryotic OTUs and higher-level taxa of the gut microbiome that were found to be differentially abundant in either PD patients, or iRBD patients or both compared to the healthy controls.

Summarized results of the differential and regression analyses of the gut microbiota:

Differential analyses of the gut microbiome at OTU level (D): The fold change values are displayed in the two innermost heatmaps in **G**. ** (confirmed by ANCOM) and bold P-values are displayed in Figure 2.

	HC	PD vs HC		iRBD vs HC	
**OTU and taxon**	mean % abundance	log_2 fold change	adj. P value	log_2 fold change	adj. P value
OTU_1809 Clostridiales	0.01	-22.73	**1.85E-10**	-5.02	0.513
OTU_1022 Proteobacteria	0.04	-22.70	**1.85E-10**	-6.94	0.304
OTU_513 *Bacteroides*	0.05	-9.48	0.001	-2.80	0.638
OTU_339 Proteobacteria	0.14	-8.39	**2.87E-04**	-1.58	0.798
OTU_1239 *Sutterella*	0.01	-6.27	0.001	-3.80	0.227
OTU_1504 Firmicutes	3.5E-03	-5.87	0.020		
OTU_1881 Ruminococcaceae	3.6E-03	-5.59	0.001	-1.82	0.557
OTU_234 *Ruminococcus*	0.05	-5.04	0.001	3.29	0.181
OTU_1977 Proteobacteria	3.3E-03	-5.03	0.022	-5.14	0.145
OTU_576 Clostridiales	0.01	-4.64	0.003	3.01	0.181
OTU_462 Prevotellaceae	0.06	-4.55	0.015	-7.05	0.001
OTU_376 *Lactobacillus*	0.02	-4.16	0.007		
OTU_353 Lachnospiraceae	0.07	-3.72	0.007		
**OTU_171 Bacteria**	0.14	-3.65	0.021	2.02	0.392
OTU_1297 *Butyricimonas*	0.01	-3.14	0.035	-1.00	0.797
OTU_1835 Lachnospiraceae	4.7E-03	-2.49	0.028	-1.97	0.181
OTU_389 Clostridiales**	0.07	-1.66	0.004	-1.34	0.091
OTU_1525 Lachnospiraceae	4.1E-03	-1.65	0.022	-0.36	0.896
OTU_299 Bacteroides	0.08	-1.46	0.022	0.23	0.936
OTU_1681 Clostridiales	3.6E-03	-1.38	0.026	-0.90	0.329
OTU_960 *Roseburia*	0.01	-1.11	0.044	-0.30	0.829
OTU_2512 **Bacteria**	6.3E-04	1.95	0.025	0.46	0.798
OTU_2773 Lachnospiraceae	3.4E-04	2.11	0.019		
OTU_1613 Ruminococcaceae	6.5E-04	2.12	0.007	1.77	0.094
OTU_426 *Clostridium IV*	0.01	2.24	0.020		
OTU_1227 Erysipelotrichaceae i.s.	2.7E-03	2.46	0.039	0.45	0.930
OTU_2484 **Bacteria**	1.0E-03	2.47	0.027	0.32	0.901
OTU_29 *Akkermansia***	0.54	2.61	0.004	1.32	0.453
OTU_257 Ruminococcaceae	0.01	2.67	**1.47E-07**		
OTU_769 Clostridiales	0.01	2.75	0.001	0.54	0.798
OTU_1476 Clostridiales	1.5E-03	3.01	0.001	0.57	0.775
OTU_2096 Bifidobacteriaceae	3.7E-04	3.77	0.015	2.60	0.181
OTU_600 *Parasutterella*	0.04	4.16	0.019		
OTU_163 *Prevotella*	0.35	4.71	1.42E-05	-1.21	0.501
OTU_1483 Fusobacteriaceae	1.6E-03	8.12	0.025	-0.01	0.999
OTU_614 Bacteroidetes	9.7E-04	11.37	7.47E-06		
OTU_1265 Ruminococcaceae	0	21.91	3.93E-10		

OTU_1495 *Olsenella*	0	22.03	**3.71E-10**		
OTU_1539 *Olsenella*	0.01	-23.62	**3.04E-17**	-8.50	0.038
OTU_184 *Bacteroides*	0.50	-6.76	**1.92E-06**	-9.70	**2.10E-10**
**OTU_469 Bacteria**	0.06	-8.13	**4.58E-04**	-8.04	0.003
OTU_519 Ruminococcaceae	0.06	-6.11	0.007	-9.00	**4.37E-04**
OTU_807 *Sutterella*	0.02	-8.33	0.001	-6.21	0.038
OTU_1507 *Bacteroides*	0.01	-6.34	0.004	-5.04	0.038
OTU_161 *Flavonifractor***	0.04	1.21	0.015	2.01	0.002
OTU_749 *Anaerotruncus*	3.4E-03	1.99	0.026	2.09	0.037
OTU_1253 *Clostridium XlVa*	4.5E-04	3.85	0.001	2.83	0.040
OTU_143 Bacteroidetes	0.07	5.60	**1.42E-05**	-4.85	0.003
OTU_872 *Butyricimonas*	0.01	-0.65	0.935	-25.31	**5.56E-25**
OTU_1284 *Megasphaera*	0.01	-0.19	0.988	-23.14	**6.40E-16**
OTU_1192 Ruminococcaceae	0.01	-4.55	0.209	-22.85	**2.52E-16**
OTU_1506 **Bacteria**	0.01	-4.73	0.489	-21.71	**2.26E-08**
OTU_1069 *Prevotella*	0.01	0.40	0.966	-21.11	**9.65E-10**
OTU_112 *Megasphaera*	0.35	0.20	0.974	-9.47	**6.43E-11**
OTU_1478 *Bacteroides*	0.01	-3.82	0.347	-9.14	0.032
OTU_223 *Mitsuokella*	0.14	0.16	0.982	-8.40	**1.97E-07**
OTU_1431 *Mitsuokella*	4.4E-03	0.15	0.992	-7.59	0.015
OTU_722 *Methanosphaera*	0.02	-3.53	0.199	-7.58	0.007
OTU_332 **Bacteria**	0.08	-1.87	0.512	-7.30	**4.64E-06**
OTU_914 *Ruminococcus*	0.01	1.69	0.833	-7.06	0.016
OTU_1928 **Bacteria**	4.4E-03	-2.43	0.512	-6.99	0.040
OTU_3172 Acidaminococcaceae	1.1E-03	-0.68	0.887	-6.84	0.023
OTU_1700 *Coprococcus*	2.7E-03	0.40	0.960	-6.79	0.017
OTU_3875 Firmicutes	4.7E-03	-0.45	0.930	-5.97	0.022
OTU_204 Prevotellaceae	0.25	-0.53	0.912	-5.43	0.001
OTU_2659 *Catenibacterium*	1.7E-03	-0.25	0.966	-5.04	0.034
OTU_1207 Firmicutes	0.01	-0.26	0.966	-4.97	0.038
OTU_57 Acidaminococcaceae	1.1	0.08	0.992	-4.89	**1.06E-04**
OTU_1427 Ruminococcaceae	4.3E-03	-0.28	0.966	-4.24	0.037
OTU_388 Lachnospiraceae	0.12	-3.08	0.074	-3.96	0.023
OTU_1183 **Bacteria**	4.2E-03	1.09	0.557	-3.56	0.038
OTU_44 *Bacteroides*	0.76	1.26	0.419	-3.15	0.008
OTU_52 *Sutterella*	0.80	-1.29	0.498	-3.06	0.032
OTU_15 *Prevotella***	7.3	-0.51	0.834	-3.04	0.010
OTU_97 Pasteurellaceae	0.09	-0.73	0.786	-2.70	0.037
OTU_1195 Gordonibacter	0.01	0.51	0.843	2.03	0.048
OTU_884 *Ruminococcus 2*	3.1E-03	1.46	0.111	2.07	0.028
OTU_502 Erysipelotrichaceae *i.s.*	0.01	1.23	0.209	2.96	0.001
OTU_145 Lachnospiraceae	0.07	1.80	0.084	3.70	**9.81E-05**

Summarized results of the regression analyses of the gut microbiome of the PD cohort at OTU level (D): These values are displayed in the three outermost heatmaps in **G**.

OTU and taxon	**MDS-UPDRS part I sum** change per unit	**MDS-UPDRS part I sum** adj. P value	**MDS-UPDRS part III sum** change per unit	**MDS-UPDRS part III sum** adj. P value	**MADRS sum** change per unit	**MADRS sum** adj. P value
OTU_1809 Clostridiales						
OTU_1022 Proteobacteria						
OTU_513 *Bacteroides*						
OTU_339 Proteobacteria	0.34	0.306	0.02	0.959		
OTU_1239 *Sutterella*						
OTU_1504 Firmicutes	0.54	0.043			0.69	0.047
OTU_1881 Ruminococcaceae						
OTU_234 *Ruminococcus*	0.40	0.150	0.02	0.957		
OTU_1977 Proteobacteria						
OTU_576 Clostridiales						
OTU_462 Prevotellaceae			-0.15	0.401		
OTU_376 *Lactobacillus*						
OTU_353 Lachnospiraceae			-0.25	0.019		
**OTU_171 Bacteria**	-0.44	0.043			-0.44	0.127
OTU_1297 *Butyricimonas*			-0.12	0.411	0.19	0.606
OTU_1835 Lachnospiraceae	-0.04	0.924	-0.06	0.440	0.07	0.840
OTU_389 Clostridiales	-0.05	0.792	-0.07	0.200	0.00	0.998
OTU_1525 Lachnospiraceae	0.08	0.572	0.02	0.864	0.15	0.493
OTU_299 Bacteroides	0.03	0.793	-0.01	0.891	0.07	0.667
OTU_1681 Clostridiales	-0.13	0.336	-0.10	0.087	-0.06	0.862
OTU_960 *Roseburia*	0.09	0.415	0.02	0.864	0.08	0.695
OTU_2512 **Bacteria**	0.02	0.937	0.09	0.145	0.21	0.216
OTU_2773 Lachnospiraceae	-0.02	0.968	0.02	0.864	0.06	0.845
OTU_1613 Ruminococcaceae	-0.07	0.714	0.04	0.716	0.16	0.504
OTU_426 *Clostridium IV*	0.15	0.474	0.11	0.346		
OTU_1227 Erysipelotrichaceae i.s.	0.00	0.990	0.04	0.724	-0.08	0.837
OTU_2484 **Bacteria**	0.32	0.049	-0.08	0.543	0.52	0.027
OTU_29 *Akkermansia*	-0.26	0.037	0.01	0.962	0.08	0.825
OTU_257 Ruminococcaceae	-0.07	0.709	0.03	0.758	0.25	0.087
OTU_769 Clostridiales			0.02	0.856	0.26	0.130
OTU_1476 Clostridiales	0.09	0.688	0.03	0.810	0.20	0.472
OTU_2096 Bifidobacteriaceae	0.13	0.709	0.15	0.179		
OTU_600 *Parasutterella*			-0.09	0.679	0.92	0.008
OTU_163 *Prevotella*	-0.26	0.329			-0.33	0.331
OTU_1483 Fusobacteriaceae	0.03	0.984	0.05	0.864	-0.30	0.616
OTU_614 Bacteroidetes					0.22	0.692
OTU_1265 Ruminococcaceae					0.57	0.187
OTU_1495 *Olsenella*					-0.29	0.621

OTU_1539 *Olsenella*						
OTU_184 *Bacteroides*			-0.17	0.149	-0.14	0.732
**OTU_469 Bacteria**	-0.67	0.011	-0.12	0.574		
OTU_519 Ruminococcaceae						
OTU_807 *Sutterella*						
OTU_1507 *Bacteroides*						
OTU_161 *Flavonifractor*	0.01	0.982	0.03	0.608	0.09	0.512
OTU_749 *Anaerotruncus*	0.34	0.011	0.16	0.036	0.36	0.047
OTU_1253 *Clostridium XlVa*	0.12	0.572	0.17	0.047	0.20	0.546
OTU_143 Bacteroidetes					-0.10	0.862
OTU_872 *Butyricimonas*	-0.64	0.011	-0.30	0.014	0.30	0.616
OTU_1284 *Megasphaera*	0.00	0.997	0.00	0.986		
OTU_1192 Ruminococcaceae			-0.19	0.271		
OTU_1506 **Bacteria**						
OTU_1069 *Prevotella*	-0.39	0.174	-0.18	0.238	-0.37	0.466
OTU_112 *Megasphaera*	-0.03	0.965				
OTU_1478 *Bacteroides*	-0.16	0.709	-0.13	0.428		
OTU_223 *Mitsuokella*	0.04	0.942			-0.13	0.825
OTU_1431 *Mitsuokella*	-0.02	0.988			-0.16	0.806
OTU_722 *Methanosphaera*						
OTU_332 **Bacteria**	-0.24	0.335	-0.10	0.505	0.19	0.608
OTU_914 *Ruminococcus*			0.10	0.677	0.89	0.023
OTU_1928 **Bacteria**	-0.08	0.902	0.00	0.986	-0.26	0.617
OTU_3172 Acidaminococcaceae	-0.06	0.902	-0.06	0.717	0.09	0.865
OTU_1700 *Coprococcus*	-0.21	0.446			-0.27	0.570
OTU_3875 Firmicutes						
OTU_204 Prevotellaceae	-0.37	0.043			-0.71	0.004
OTU_2659 *Catenibacterium*	-0.06	0.935	-0.04	0.864	-0.12	0.870
OTU_1207 Firmicutes	-0.30	0.284	-0.15	0.294	-0.32	0.501
OTU_57 Acidaminococcaceae	-0.01	0.984	-0.01	0.955	0.14	0.670
OTU_1427 Ruminococcaceae			-0.08	0.720		
OTU_388 Lachnospiraceae	0.16	0.562	0.08	0.580		
OTU_1183 **Bacteria**	0.13	0.424	0.04	0.740	-0.01	0.984
OTU_44 *Bacteroides*			0.06	0.550	0.02	0.968
OTU_52 *Sutterella*	-0.02	0.966	-0.08	0.549	-0.02	0.965
OTU_15 *Prevotella*	-0.14	0.335	-0.07	0.411	-0.17	0.504
OTU_97 Pasteurellaceae	0.05	0.880			-0.02	0.973
OTU_1195 Gordonibacter			0.05	0.750	0.41	0.004
OTU_884 *Ruminococcus 2*	0.05	0.842	-0.02	0.864	0.25	0.068
OTU_502 Erysipelotrichaceae *i.s.*	0.08	0.596			0.07	0.788
OTU_145 Lachnospiraceae	-0.01	0.984	0.16	0.027		

Summarized results of the differential analyses of the gut microbiome at higher taxonomic levels (E): These values are displayed in the two innermost heatmaps in **G**. ** (confirmed by ANCOM and P-values < 0.001 are displayed in **Figure 2**.

taxon	HC mean % abundance	PD vs HC log$_2$ fold change	PD vs HC adj. P value	iRBD vs HC log$_2$ fold change	iRBD vs HC adj. P value
*Akkermansia***	0.63	2.23	0.014	1.52	0.292
Clostridium XlVb	0.01	1.88	0.011	1.58	0.024
Anaerotruncus	0.02	1.96	0.010	1.64	0.020
Mitsuokella	1.9E-03	-1.35	0.848	-7.83	**4.47E-09**
Megasphaera	0.02	0.23	0.974	-7.61	**1.56E-10**
Methanosphaera	0.11	-3.79	0.246	-7.45	0.004
Catenibacterium	0.13	-1.54	0.908	-6.05	0.005
*Prevotella***	0.41	-0.53	0.974	-2.81	0.004
Erysipelotrichaceae *incertae sedis*	0.02	0.69	0.723	1.81	0.020
Flavonifractor	0.25	0.03	0.977	2.06	**9.52E-05**
Eggerthella	12.4	-1.04	0.663	2.14	0.042
Verrucomicrobiaceae**	0.63	2.20	0.035	1.71	0.340
Prevotellaceae**	13.6	-0.46	0.981	-2.44	0.017
Verrucomicrobiales**	0.00	2.08	0.017	1.30	0.431
Actinomycetales**	0.63	2.58	0.001	0.77	0.626
Pasteurellales	0.10			-2.76	0.034
Verrucomicrobiae**	0.63	2.13	0.012	1.40	0.352
Verrucomicrobia**	0.64	2.06	0.007	1.48	0.160

Summarized results of the regression analyses of the gut microbiome of the PD cohort at higher taxonomic levels (E): These values are displayed in the three outermost heatmaps in **G**.

taxon	**MDS-UPDRS part I sum** change per unit	adj. P value	**MDS-UPDRS part III sum** change per unit	adj. P value	**MADRS sum** change per unit	adj. P value
Akkermansia	-0.23	0.016	0.00	0.981	0.00	0.996
Clostridium XlVb	0.14	0.290	0.12	0.040	0.26	0.163
Anaerotruncus	0.26	**4.39E-04**	0.11	0.040	0.31	0.022
Mitsuokella	0.01	0.962			-0.16	0.873
Megasphaera	-0.06	0.928				
Methanosphaera					-0.36	0.873
Catenibacterium			-0.04	0.902	-0.12	0.932
Prevotella	-0.03	0.938	-0.08	0.417	-0.07	0.873
Erysipelotrichaceae *incertae sedis*	-0.08	0.443	-0.08	0.040	-0.09	0.873
Flavonifractor	0.00	0.962	0.01	0.902	0.01	0.996
Eggerthella			-0.03	0.902	-0.28	0.247
Verrucomicrobiaceae	-0.21	0.040	0.01	0.876	0.04	0.968
Prevotellaceae	-0.05	0.826	-0.09	0.195	-0.09	0.705
Verrucomicrobiales	-0.20	0.021	0.00	0.951	0.03	0.960
Actinomycetales	0.22	0.010	0.02	0.797	0.22	0.368
Pasteurellales					0.08	0.940
Verrucomicrobiae	-0.20	0.024	0.01	0.865	0.03	0.941
Verrucomicrobia	-0.17	0.107	0.00	0.969	0.00	0.934

Supplementary References

1. Mathay C, Hamot G, Henry E, et al. Method Optimization for Fecal Sample Collection and Fecal DNA Extraction. Biopreserv Biobank 2015;13:79–93.

2. Suzuki MT, Taylor LT, DeLong EF. Quantitative analysis of small-subunit rRNA genes in mixed microbial populations via 5'-nuclease assays. Applied and Environmental Microbiology 2000;66:4605–14.

3. Hugerth LW, Wefer HA, Lundin S. DegePrime, a program for degenerate primer design for broad-taxonomic-range PCR in microbial ecology studies. Applied and Environmental Microbiology 2014;80:5116-23.

4. Herlemann DP, Labrenz M, Jürgens K, Bertilsson S, Waniek JJ, Andersson AF. Transitions in bacterial communities along the 2000 km salinity gradient of the Baltic Sea. The ISME Journal 2011;5:1571–9.

5. Hugerth LW, Muller EEL, Hu YOO, et al. Systematic Design of 18S rRNA Gene Primers for Determining Eukaryotic Diversity in Microbial Consortia. PLoS ONE 2014;9:e95567.

6. Hildebrand F, Tadeo R, Voigt AY, Bork P, Raes J. LotuS: an efficient and user-friendly OTU processing pipeline. Microbiome 2014;2:30.

7. R Core Team. R: A Language and Environment for Statistical Computing. Vienna, Austria: 2014.

8. Hu YOO, Karlson B, Charvet S, Andersson AF. Diversity of Pico- to Mesoplankton along the 2000 km Salinity Gradient of the Baltic Sea. Frontiers in Microbiology

2016;7:679.

9. Guillou L, Bachar D, Audic S, et al. The Protist Ribosomal Reference database (PR2): a catalog of unicellular eukaryote Small Sub-Unit rRNA sequences with curated taxonomy. Nucleic Acids Research 2012;41:D597–D604.

10. Narayanasamy S, Jarosz Y, Muller EEL, et al. IMP: a pipeline for reproducible metagenomic and metatranscriptomic analyses. Genome Biol 2016;17:260.

11. Eddy SR. Accelerated Profile HMM Searches. PLoS Comput Biol 2011;7:e1002195.

12. Heintz-Buschart A, May P, Laczny CC, et al. Integrated multi-omics of the human gut microbiome in a case study of familial type 1 diabetes. Nature Microbiology 2016;2:16180.

13. Dueholm MS, Albertsen M, Otzen D, Nielsen PH. Curli Functional Amyloid Systems Are Phylogenetically Widespread and Display Large Diversity in Operon and Protein Structure. PLoS ONE 2012;7:e51274.

14. Fu L, Niu B, Zhu Z, Wu S, Li W. CD-HIT: accelerated for clustering the next-generation sequencing data. Bioinformatics 2012;28:3150–2.

15. Langmead B, Salzberg SL. Fast gapped-read alignment with Bowtie 2. Nat Methods 2012;9:357–9.

16. Mende DR, Sunagawa S, Zeller G, Bork P. Accurate and universal delineation of prokaryotic species. Nat Methods 2013;10:881–4.

17. Kultima JR, Sunagawa S, Li J, et al. MOCAT: a metagenomics assembly and gene prediction toolkit. PLoS ONE 2012;7:e47656.

18. Sunagawa S, Mende DR, Zeller G, et al. Metagenomic species profiling using universal phylogenetic marker genes. Nat Methods 2013;10:1196–9.

19. Nielsen HB, Almeida M, Juncker AS, et al. Identification and assembly of genomes and genetic elements in complex metagenomic samples without using reference genomes. Nat Biotechnol 2014;32:822–8.

20. Aziz RK, Bartels D, Best AA, et al. The RAST Server: rapid annotations using subsystems technology. BMC Genomics 2008;9:75.

21. Laczny CC, Muller EEL, Heintz-Buschart A, et al. Identification, Recovery, and Refinement of Hitherto Undescribed Population-Level Genomes from the Human Gastrointestinal Tract. Frontiers in Microbiology 2016;7:533.

22. Di Rienzi SC, Sharon I, Wrighton KC, et al. The human gut and groundwater harbor non-photosynthetic bacteria belonging to a new candidate phylum sibling to Cyanobacteria. eLife 2013;2:e01102.

23. Rantala-Ylinen A, Kana S, Wang H, et al. Anatoxin-a Synthetase Gene Cluster of the Cyanobacterium Anabaena sp. Strain 37 and Molecular Methods To Detect Potential Producers. Applied and Environmental Microbiology 2011;77:7271–8.

24. Méjean A, Paci G, Gautier V, Ploux O. Biosynthesis of anatoxin-a and analogues (anatoxins) in cyanobacteria. Toxicon 2014;91:15–22.

25. Kellmann R, Mihali TK, Jeon YJ, Pickford R, Pomati F, Neilan BA. Biosynthetic Intermediate Analysis and Functional Homology Reveal a Saxitoxin Gene Cluster in Cyanobacteria. Applied and Environmental Microbiology 2008;74:4044–53.
