# Supplementary material for: The nasal and gut microbiome in Parkinson's disease and idiopathic rapid eye movement sleep behavior disorder
Source: Mov Disord. 2017 Aug 26;33(1):88–98. doi: 10.1002/mds.27105 (PMC5811909; doi:10.1002/mds.27105)
Supplement: Supplementary file 2 — Supplementary TABLE 1. Cohort characteristics; technical details relating to the sequenced patient samples and the control samples. Supplementary TABLE 2. Numbers of reads per sample and OTU, genus, family, order, class, or phylum for 16S rRNA gene amplicon sequencing data; numbers of reads per sample and OTU or division for 18S rRNA gene amplicon sequencing data; estimated relative abundances of KEGG (Kyoto Encyclopedia of Genes and Genomes) orthologous groups per sample; results of differential analysis of predicted KEGG orthologous groups in PD versus HC and iRBD versus HC; gene functions in the genome reconstructions of OTU_171 and OTU_469; and results of search for cyanobacterial toxin genes in the genome of OTU_171. Supplementary Information: Supplementary methods; explanation for Figure 2, detailed plots, numerical summaries, and statistics of the differential analyses; supplementary references. [file MDS-33-88-s002.pdf]

Characteristics of the whole cohort

Study group	HC	PD	iRBD
N	78	76	21
**Demographics**			
Male/female (% male)	46/32 (59%)	50/26 (66%)	12/9 (57%)
age, years [mean +/- standard deviation]	68.45 +/- 6.71	68.04 +/- 9.7	66.14 +/- 7.93
**Anthropometrics**			
weight, kg [mean +/- standard deviation]	80.15 +/- 16.02	83.68 +/- 15.52	78.01 +/- 14.28
height, cm [mean +/- standard deviation]	173.26 +/- 8.84	171.4 +/- 9.66	172.38 +/- 10.05
BMI, kg/(m*m) [mean +/- standard deviation]	26.54 +/- 4.07	28.46 +/- 4.52	26.15 +/- 3.44
**Motor Symptoms**			
Hoehn and Yahr staging [mean +/- standard deviation]	0 +/- 0	2.13 +/- 0.8	0.05 +/- 0.22
MDS-UPDRS II, sum [mean +/- standard deviation]	1.47 +/- 2.04	13.15 +/- 7.43	3.19 +/- 2.98
MDS-UPDRS III, sum [mean +/- standard deviation]	1.54 +/- 2.8	29.69 +/- 13.58	3.76 +/- 3.24
MDS-UPDRS IV, sum [mean +/- standard deviation]	0 +/- 0	1.06 +/- 1.81	0 +/- 0
MDS-UPDRS, sum [mean +/- standard deviation]	8.47 +/- 7.76	55.62 +/- 24.84	18.81 +/- 9.35
**Non-motor symptoms**			
MDS-UPDRS I, sum [mean +/- standard deviation]	5.46 +/- 4.3	11.72 +/- 7.22	11.86 +/- 6
MDS-UPDRS anxiety (Question I.4) [mean +/- standard deviation]	0.23 +/- 0.53	0.67 +/- 0.84	1.05 +/- 0.97
MDS-UPDRS pain (Question I.9) [mean +/- standard deviation]	0.69 +/- 0.83	1.28 +/- 1.12	0.9 +/- 0.7
MDS-UPDRS constipation (Question I.11) [mean +/- standard deviation]	0.27 +/- 0.7	1.23 +/- 1.13	0.95 +/- 1.2
Scopa-AUT, sum [mean +/- standard deviation]	8.37 +/- 5.67	13.09 +/- 7.09	12.38 +/- 5.45
Scopa-AUT gastrointestinal part (Questions 1-7), sum [mean +/- standard deviation]	0.91 +/- 1.11	3.18 +/- 2.36	3 +/- 1.97
Scopa-AUT constipation (Question 5)	often:2 (3%); sometimes:5 (6%); never:71 (91%)	often:4 (5%); regularly:2 (3%); sometimes:25 (34%); never:43 (58%); 2 n.d.	often:1 (5%); regularly:1 (5%); sometimes:9 (43%); never:10 (48%);
NMS-Quest constipation (Question 5), yes/no (%)	9/69 (12%)	38/38 (50%)	8/13 (38%)
Non-motor symptom assessment scale (NMSS) constipation (Question 21, strength*frequency) [mean +/- standard deviation]	0.04 +/- 0.25	0.45 +/- 1.52	0.67 +/- 1.43
constipation by interview, N (%)	1 (1%)	15 (20%)	n.d.
other GI dysfunction by interview, N (%)	17 (22%)	24 (32%)	n.d.
Beck depression inventory (BDI), sum [mean +/- standard deviation]	3.71 +/- 4.47	7.35 +/- 5.92	6.79 +/- 5.55
Geriatric Depression Scale (GDS), sum [mean +/- standard deviation]	1.34 +/- 2.2	2.55 +/- 2.61	2.62 +/- 2.36
Epworth Sleepiness Scale (ESS), sum [mean +/- standard deviation]	6.02 +/- 3.69	7.86 +/- 3.84	9.5 +/- 4.36
Mini-Mental-State-Examination (MMSE), total score [mean +/- standard deviation]	28.71 +/- 1.53	27.72 +/- 2.35	28.67 +/- 0.86
Montreal Cognitive Assessment (MoCa), total score [mean +/- standard deviation]	26.1 +/- 2.73	24.32 +/- 4.2	23.81 +/- 3.2
Montgomery-Åsberg Depression Rating Scale (MADRS), sum [mean +/- standard deviation]	1.89 +/- 5.05	2.79 +/- 3.78	5.24 +/- 6.14
RBD by polysomnography, N	0	40; 6 n.d.	21
Smell identification test (by 16 item Sniffin Sticks) [mean +/- standard deviation]	11.81 +/- 2.59	5.91 +/- 3.45	6.4 +/- 3.93
**Intake of PD medication**			
Azilect, N (%)	0 (0%)	50 (66%)	0 (0%)
any MAO-B inhibitors, N (%)	0 (0%)	52 (68%)	0 (0%)
Levodopa, N (%)	0 (0%)	66, (87%)	0 (0%)
L-Dopa agonists, N (%)	0 (0%)	52 (68%)	0 (0%)
COMT inhibitors, N (%)	0 (0%)	4 (5%)	0 (0%)
OTHER: macrogol intake, N (%)	1 (1%)	8 (11%)	0 (0%)
**Medical history**			
depression, N (%)	11 (14%)	7 (9%)	6 (29%)
anxiety, N (%)	2 (3%)	4 (5%)	3, 1 n.d. (15%)
sleep apnoe, N (%)	8 (10%)	2 (3%)	3 (14%)
periodic limb movement disorder, N (%)	0 (0%)	1 (1%)	1 (5%)
hypertension, N (%)	36 (46%)	43 (56%)	10 (48%)
hyperlipidemia, N (%)	46 (59%)	31 (41%)	7 (33%)
diabetes mellitus, N (%)	4 (5%)	6 (8%)	3 (14%)
osteoathritis, N (%)	37 (47%)	19 (25%)	11 (52%)
rheumatoid arthritis, N (%)	1 (1%)	6 (8%)	0 (0%)
coronary artery disease, N (%)	2 (3%)	8 (11%)	2 (10%)
congestive heart disease, N (%)	2 (3%)	3 (4%)	1 (5%)
hypotheroidism, N (%)	7 (9%)	7 (9%)	3 (14%)
asthma/COPD, N (%)	9 (12%)	6 (8%)	5 (24%)
cancer, N (%)	4 (5%)	5 (7%)	0 (0%)
antidepressant intake, N (%)	5, 1 n.d. (6%)	5 (7%)	0 (0%)
current diabetes and/or metformin intake, N (%)	6 (8%)	7 (9%)	3 (14%)
metformin intake, N (%)	3 (4%)	4 (5%)	3 (14%)

Characteristics of the cohort with nasal wash samples

Study group	HC	PD	iRBD
N	65	70	12
**Demographics**			
Male/female (% male)	36/29 (55%)	47/23 (67%)	7/5 (58%)
age, years [mean +/- standard deviation]	68.32 +/- 6.28	68.14 +/- 10.06	68 +/- 7.32
**Anthropometrics**			
weight, kg [mean +/- standard deviation]	80.31 +/- 16.36	83.59 +/- 15.58	79.54 +/- 16.14
height, cm [mean +/- standard deviation]	172.89 +/- 8.56	171.64 +/- 9.69	172.17 +/- 10.94
BMI, kg/(m*m) [mean +/- standard deviation]	26.71 +/- 4.26	28.34 +/- 4.44	26.65 +/- 3.48
**Motor Symptoms**			
Hoehn and Yahr staging [mean +/- standard deviation]	0 +/- 0	2.16 +/- 0.82	0.08 +/- 0.29
MDS-UPDRS II, sum [mean +/- standard deviation]	1.45 +/- 1.93	13.54 +/- 7.56	3.08 +/- 3.09
MDS-UPDRS III, sum [mean +/- standard deviation]	1.62 +/- 2.72	29.99 +/- 13.91	3.83 +/- 3.38
MDS-UPDRS IV, sum [mean +/- standard deviation]	0 +/- 0	1.12 +/- 1.87	0 +/- 0
MDS-UPDRS, sum [mean +/- standard deviation]	8.6 +/- 7.75	56.35 +/- 25.5	17.17 +/- 9.79
**Non-motor symptoms**			
MDS-UPDRS I, sum [mean +/- standard deviation]	5.54 +/- 4.49	11.71 +/- 7.36	10.25 +/- 5.03
MDS-UPDRS anxiety (Question I.4) [mean +/- standard deviation]	0.28 +/- 0.57	0.64 +/- 0.82	0.92 +/- 1
MDS-UPDRS pain (Question I.9) [mean +/- standard deviation]	0.68 +/- 0.85	1.26 +/- 1.13	0.83 +/- 0.72
MDS-UPDRS constipation (Question I.11) [mean +/- standard deviation]	0.31 +/- 0.75	1.26 +/- 1.13	0.5 +/- 0.9
Scopa-AUT, sum [mean +/- standard deviation]	8.72 +/- 5.67	13.05 +/- 7.29	10.42 +/- 4.72
Scopa-AUT gastrointestinal part (Questions 1-7), sum [mean +/- standard deviation]	0.98 +/- 1.17	3.14 +/- 2.35	2.08 +/- 1.51
Scopa-AUT constipation (Question 5)	often:2 (3%); sometimes:4 (6%); never:59 (91%)	often:4 (6%); regularly:1 (1%); sometimes:25 (37%); never:38 (56%); 2 n.d.	sometimes:4 (33%); never:8 (67%);
NMS-Quest constipation (Question 5), yes/no (%)	9/56 (16%)	36/34 (51%)	3/9 (25%)
Non-motor symptom assessment scale (NMSS) constipation (Question 21, strength*frequency) [mean +/- standard deviation]	0.05 +/- 0.28	0.49 +/- 1.58	0.67 +/- 1.56
constipation by interview, N (%)	1 (2%)	13 (19%)	n.d.
other GI dysfunction by interview, N (%)	16 (25%)	23 (33%)	n.d.
Beck depression inventory (BDI), sum [mean +/- standard deviation]	3.89 +/- 4.77	7.17 +/- 5.7	5.25 +/- 4.64
Geriatric Depression Scale (GDS), sum [mean +/- standard deviation]	1.44 +/- 2.35	2.55 +/- 2.67	1.76 +/- 1.43
Epworth Sleepiness Scale (ESS), sum [mean +/- standard deviation]	6.15 +/- 3.83	7.72 +/- 3.71	n.d.
Mini-Mental-State-Examination (MMSE), total score [mean +/- standard deviation]	28.71 +/- 1.53	27.72 +/- 2.35	28.67 +/- 0.86
Montreal Cognitive Assessment (MoCa), total score [mean +/- standard deviation]	26.02 +/- 2.48	24.21 +/- 4.29	23.33 +/- 3.68
Montgomery-Åsberg Depression Rating Scale (MADRS), sum [mean +/- standard deviation]	1.97 +/- 5.46	2.74 +/- 3.66	3.25 +/- 3.19
RBD by polysomnography, N	0	37; 5 n.d.	12
Smell identification test (by 16 item Sniffin Sticks) [mean +/- standard deviation]	11.88 +/- 2.56	5.8 +/- 3.52	7 +/- 3.22
**Intake of PD medication**			
Azilect, N (%)	0 (0%)	44 (63%)	0 (0%)
any MAO-B inhibitors, N (%)	0 (0%)	46 (66%)	0 (0%)
Levodopa, N (%)	0 (0%)	62, (89%)	0 (0%)
L-Dopa agonists, N (%)	0 (0%)	46 (66%)	0 (0%)
COMT inhibitors, N (%)	0 (0%)	4 (6%)	0 (0%)
OTHER: macrogol intake, N (%)	1 (2%)	7 (10%)	0 (0%)
**Medical history**			
depression, N (%)	11 (17%)	7 (10%)	1 (8%)
anxiety, N (%)	2 (3%)	4 (6%)	0, 1 n.d. (0%)
sleep apnoe, N (%)	7 (11%)	2 (3%)	2 (17%)
periodic limb movement disorder, N (%)	0 (0%)	1 (1%)	1 (8%)
hypertension, N (%)	30 (46%)	39 (56%)	6 (50%)
hyperlipidemia, N (%)	39 (60%)	28 (40%)	3 (25%)
diabetes mellitus, N (%)	4 (6%)	6 (9%)	2 (17%)
osteoathritis, N (%)	34 (52%)	18 (26%)	8 (67%)
rheumatoid arthritis, N (%)	1 (2%)	6 (9%)	0 (0%)
coronary artery disease, N (%)	2 (3%)	6 (9%)	1 (8%)
congestive heart disease, N (%)	2 (3%)	3 (4%)	1 (8%)
hypotheroidism, N (%)	7 (6%)	5 (7%)	3 (25%)
asthma/COPD, N (%)	9 (14%)	6 (9%)	3 (25%)
cancer, N (%)	4 (6%)	5 (7%)	0, 2 n.d. (0%)
antidepressant intake, N (%)	5, 1 n.d. (6%)	5 (7%)	0 (0%)
current diabetes and/or metformin intake, N (%)	6 (9%)	6 (9%)	2 (17%)
metformin intake, N (%)	3 (5%)	3 (4%)	2 (17%)

Characteristics of the cohort with stool samples analyzed by 16S rRNA gene amplicon sequencing

**Study group**	**HC**	**PD**	**iRBD**
N	38	26	20
**Demographics**			
Male/female (% male)	25/13 (66%)	19/7 (73%)	12/8 (60%)
age, years [mean +/- standard deviation]	69.84 +/- 6.6	65.96 +/- 8.01	66.05 +/- 8.13
**Anthropometrics**			
weight, kg [mean +/- standard deviation]	80.55 +/- 14.92	85.09 +/- 13.69	78.76 +/- 14.22
height, cm [mean +/- standard deviation]	172.55 +/- 8.92	173.17 +/- 9.19	172.75 +/- 10.17
BMI, kg/(m*m) [mean +/- standard deviation]	26.9 +/- 3.68	28.47 +/- 4.05	26.3 +/- 3.46
**Motor Symptoms**			
Hoehn and Yahr staging [mean +/- standard deviation]	0 +/- 0	2 +/- 0.58	0.05 +/- 0.22
MDS-UPDRS II, sum [mean +/- standard deviation]	1.42 +/- 1.93	11.38 +/- 5.39	3.35 +/- 2.96
MDS-UPDRS III, sum [mean +/- standard deviation]	1.5 +/- 2.83	27.46 +/- 11.81	3.95 +/- 3.2
MDS-UPDRS IV, sum [mean +/- standard deviation]	0 +/- 0	0.69 +/- 1.26	0 +/- 0
MDS-UPDRS, sum [mean +/- standard deviation]	8.97 +/- 7.33	49.81 +/- 20.11	19.55 +/- 8.94
**Non-motor symptoms**			
MDS-UPDRS I, sum [mean +/- standard deviation]	6.05 +/- 4.05	10.27 +/- 6.35	12.25 +/- 5.87
MDS-UPDRS anxiety (Question I.4) [mean +/- standard deviation]	0.26 +/- 0.6	0.5 +/- 0.76	1.1 +/- 0.97
MDS-UPDRS pain (Question I.9) [mean +/- standard deviation]	0.68 +/- 0.77	1.08 +/- 1.2	0.95 +/- 0.69
MDS-UPDRS constipation (Question I.11) [mean +/- standard deviation]	0.32 +/- 0.77	0.77 +/- 0.95	1 +/- 1.21
Scopa-AUT, sum [mean +/- standard deviation]	7.58 +/- 5.58	13 +/- 6.41	12.3 +/- 5.58
Scopa-AUT gastrointestinal part (Questions 1-7), sum [mean +/- standard deviation]	0.82 +/- 1.09	3.16 +/- 2.36	3.1 +/- 1.97
Scopa-AUT constipation (Question 5)	often:2 (5%); sometimes:4 (11%); never:32 (84%)	often:1 (4%); regularly:1 (4%); sometimes:8 (31%); never:16 (62%)	often:1 (5%); regularly:1 (5%); sometimes:9 (45%); never:9 (45%);
NMS-Quest constipation (Question 5), yes/no (%)	4/34 (11%)	11/15 (42%)	8/12 (40%)
Non-motor symptom assessment scale (NMSS) constipation (Question 21, strength*frequency) [mean +/- standard deviation]	0.08 +/- 0.36	0 +/- 0	0.7 +/- 1.45
constipation by interview, N (%)	1 (3%)	4 (15%)	n.d.
other GI dysfunction by interview, N (%)	6 (16%)	8 (33%)	n.d.
Beck depression inventory (BDI), sum [mean +/- standard deviation]	2.95 +/- 3.02	6.76 +/- 7.01	6.47 +/- 5.51
Geriatric Depression Scale (GDS), sum [mean +/- standard deviation]	1.11 +/- 1.54	2.53 +/- 2.57	2.55 +/- 2.4
Epworth Sleepiness Scale (ESS), sum [mean +/- standard deviation]	5.56 +/- 3.21	8.27 +/- 3.69	9.5 +/- 4.36
Mini-Mental-State-Examination (MMSE), total score [mean +/- standard deviation]	28.5 +/- 1.91	28.5 +/- 1.7	28.75 +/- 0.79
Montreal Cognitive Assessment (MoCa), total score [mean +/- standard deviation]	25.76 +/- 3.03	25.46 +/- 3.54	23.75 +/- 3.27
Montgomery-Åsberg Depression Rating Scale (MADRS), sum [mean +/- standard deviation]	1.69 +/- 4.6	3.19 +/- 4.31	5.15 +/- 6.29
RBD by polysomnography, N	0	13; 2 n.d.	20
Smell identification test (by 16 item Sniffin Sticks) [mean +/- standard deviation]	11.5 +/- 2.86	5.35 +/- 2.99	6.21 +/- 3.94
**Intake of PD medication**			
Azilect, N (%)	0 (0%)	20 (77%)	0 (0%)
any MAO-B inhibitors, N (%)	0 (0%)	21 (81%)	0 (0%)
Levodopa, N (%)	0 (0%)	20, (77%)	0 (0%)
L-Dopa agonists, N (%)	0 (0%)	22 (85%)	0 (0%)
COMT inhibitors, N (%)	0 (0%)	1 (4%)	0 (0%)
OTHER: macrogol intake, N (%)	1 (3%)	2 (8%)	0 (0%)
**Medical history**			
depression, N (%)	4 (11%)	0 (0%)	6 (30%)
anxiety, N (%)	0 (0%)	0 (0%)	3, 1 n.d. (16%)
sleep apnoe, N (%)	6 (16%)	1 (4%)	3 (15%)
periodic limb movement disorder, N (%)	0 (0%)	0 (0%)	1 (5%)
hypertension, N (%)	18 (47%)	12 (46%)	10 (50%)
hyperlipidemia, N (%)	23 (61%)	7 (27%)	7 (35%)
diabetes mellitus, N (%)	3 (8%)	0 (0%)	3 (15%)
osteoathritis, N (%)	14 (37%)	3 (12%)	10 (50%)
rheumatoid arthritis, N (%)	1 (3%)	2 (8%)	0 (0%)
coronary artery disease, N (%)	1 (3%)	2 (8%)	2 (10%)
congestive heart disease, N (%)	2 (5%)	2 (8%)	1 (5%)
hypotheroidism, N (%)	4 (11%)	3 (12%)	3 (15%)
asthma/COPD, N (%)	4 (11%)	0 (0%)	5 (25%)
cancer, N (%)	1 (3%)	0 (0%)	0 (0%)
antidepressant intake, N (%)	2, 1 n.d. (6%)	1 (4%)	0 (0%)
current diabetes and/or metformin intake, N (%)	3 (8%)	1 (4%)	3 (15%)
metformin intake, N (%)	1 (3%)	1 (4%)	3 (15%)

Characteristics of the cohort with stool samples analyzed by 18S rRNA gene amplicon sequencing

**Study group**	**HC**	**PD**	**iRBD**
N	32	14	15
**Demographics**			
Male/female (% male)	20/12 (63%)	10/4 (71%)	9/6 (50%)
age, years [mean +/- standard deviation]	69.31 +/- 5.9	65.43 +/- 6.57	65.87 +/- 7.86
**Anthropometrics**			
weight, kg [mean +/- standard deviation]	79.89 +/- 14.92	84.27 +/- 13.42	81.75 +/- 14.74
height, cm [mean +/- standard deviation]	173.12 +/- 8.43	173.61 +/- 10.66	173.73 +/- 11.1
BMI, kg/(m*m) [mean +/- standard deviation]	26.51 +/- 3.77	28 +/- 4.12	27.02 +/- 3.69
**Motor Symptoms**			
Hoehn and Yahr staging [mean +/- standard deviation]	0 +/- 0	2.04 +/- 0.66	0.07 +/- 0.26
MDS-UPDRS II, sum [mean +/- standard deviation]	1.16 +/- 1.55	10.29 +/- 4.87	3.27 +/- 2.55
MDS-UPDRS III, sum [mean +/- standard deviation]	1.34 +/- 2.39	26.93 +/- 12.72	3.67 +/- 3.31
MDS-UPDRS IV, sum [mean +/- standard deviation]	0 +/- 0	0.93 +/- 1.49	0 +/- 0
MDS-UPDRS, sum [mean +/- standard deviation]	8.59 +/- 6.67	48.57 +/- 21.06	19.6 +/- 8.85
**Non-motor symptoms**			
MDS-UPDRS I, sum [mean +/- standard deviation]	6.09 +/- 4.21	10.43 +/- 7.23	12.67 +/- 5.89
MDS-UPDRS anxiety (Question I.4) [mean +/- standard deviation]	0.28 +/- 0.63	0.64 +/- 0.84	1.2 +/- 0.86
MDS-UPDRS pain (Question I.9) [mean +/- standard deviation]	0.75 +/- 0.8	0.93 +/- 1.27	1.07 +/- 0.59
MDS-UPDRS constipation (Question I.11) [mean +/- standard deviation]	0.38 +/- 0.83	0.86 +/- 1.03	1 +/- 1.2
Scopa-AUT, sum [mean +/- standard deviation]	7.72 +/- 5.85	13.18 +/- 6.29	12.27 +/- 6.31
Scopa-AUT gastrointestinal part (Questions 1-7), sum [mean +/- standard deviation]	0.84 +/- 1.14	3.08 +/- 2.5	3.33 +/- 2.16
Scopa-AUT constipation (Question 5)	often:2 (6%);<br>sometimes:3 (9%);<br>never:27 (84%)	regularly:1 (8%);<br>sometimes:5 (38%);<br>never:8 (62%);<br>1 n.d.	often:1 (7%);<br>regularly:1 (7%);<br>sometimes:7 (47%);<br>never:6 (40%);
NMS-Quest constipation (Question 5), yes/no (%)	4/28 (13%)	6/8 (43%)	7/8 (47%)
Non-motor symptom assessment scale (NMSS) constipation (Question 21, strength*frequency) [mean +/- standard deviation]	0.09 +/- 0.39	0 +/- 0	0.67 +/- 1.4
constipation by interview, N (%)	1 (3%)	2 (14%)	n.d.
other GI dysfunction by interview, N (%)	6 (19%)	4 (29%)	n.d.
Beck depression inventory (BDI), sum [mean +/- standard deviation]	3.1 +/- 3.16	7.67 +/- 7.89	6.37 +/- 6.26
Geriatric Depression Scale (GDS), sum [mean +/- standard deviation]	1.1 +/- 1.51	3.26 +/- 3.13	2.94 +/- 2.61
Epworth Sleepiness Scale (ESS), sum [mean +/- standard deviation]	5.62 +/- 3.17	8.36 +/- 3.1	9.5 +/- 4.36
Mini-Mental-State-Examination (MMSE), total score [mean +/- standard deviation]	28.91 +/- 1.38	28.57 +/- 2.03	28.67 +/- 0.82
Montreal Cognitive Assessment (MoCa), total score [mean +/- standard deviation]	26.19 +/- 2.26	25 +/- 3.74	23.33 +/- 3.39
Montgomery-Åsberg Depression Rating Scale (MADRS), sum [mean +/- standard deviation]	1.87 +/- 4.92	3.5 +/- 4.82	6.53 +/- 6.67
RBD by polysomnography, N	0	9; 1 n.d.	15
Smell identification test (by 16 item Sniffin Sticks) [mean +/- standard deviation]	11.47 +/- 2.66	5.43 +/- 2.98	5.86 +/- 3.84
**Intake of PD medication**			
Azilect, N (%)	0 (0%)	9 (64%)	0 (0%)
any MAO-B inhibitors, N (%)	0 (0%)	10 (71%)	0 (0%)
Levodopa, N (%)	0 (0%)	12 (86%)	0 (0%)
L-Dopa agonists, N (%)	0 (0%)	12 (86%)	0 (0%)
COMT inhibitors, N (%)	0 (0%)	1 (7%)	0 (0%)
OTHER: macrogol intake, N (%)	1 (3%)	2 (14%)	0 (0%)
**Medical history**			
depression, N (%)	4 (13%)	0 (0%)	4 (27%)
anxiety, N (%)	0 (0%)	0 (0%)	2, 1 n.d. (14%)
sleep apnoe, N (%)	4 (13%)	0 (0%)	3 (20%)
periodic limb movement disorder, N (%)	0 (0%)	0 (0%)	1 (7%)
hypertension, N (%)	14 (44%)	5 (36%)	6 (40%)
hyperlipidemia, N (%)	20 (63%)	3 (21%)	4 (27%)
diabetes mellitus, N (%)	2 (6%)	0 (0%)	1 (7%)
osteoathritis, N (%)	14 (44%)	2 (14%)	7 (47%)
rheumatoid arthritis, N (%)	1 (3%)	2 (14%)	0 (0%)
coronary artery disease, N (%)	1 (3%)	0 (0%)	1 (7%)
congestive heart disease, N (%)	2 (6%)	1 (7%)	1 (7%)
hypotheroidism, N (%)	3 (9%)	2 (14%)	3 (20%)
asthma/COPD, N (%)	3 (9%)	0 (0%)	4 (27%)
cancer, N (%)	0 (0%)	0 (0%)	0 (0%)
antidepressant intake, N (%)	1, 1 n.d. (6%)	1 (7%)	0 (0%)
current diabetes and/or metformin intake, N (%)	2 (6%)	1 (7%)	1 (7%)
metformin intake, N (%)	1 (3%)	1 (7%)	1 (7%)

comparison PD versus HC						
KEGG orthologous group	HC mean % abundan	greater median in	estimate of location parameter	P-Value (Wilcoxon rank sum test)	adjusted P-value	KEGG description
K13038	0.069%	HC	-0.0049%	0.004	1.0	dfp; fused 4'-phosphopantothenoylcysteine decarboxylase/phosphopantothenoylcysteine synthetase, FMN-binding (EC:4.1.1.36 6.3.2.5); K13038 phosphopantothenoylcysteine decarboxylase / phosphopantothenate--cysteine ligase [EC:4.1.1.36 6.3.2.5] (A)
K01749	0.022%	PD	0.0032%	0.009	1.0	HMBS, PBG-D, PBGD, PORC, UPS; hydroxymethylbilane synthase (EC:2.5.1.61); K01749 hydroxymethylbilane synthase [EC:2.5.1.61] (A)
K01845	0.032%	PD	0.0019%	0.006	1.0	GSA2; glutamate-1-semialdehyde 2,1-aminomutase 2; K01845 glutamate-1-semialdehyde 2,1-aminomutase [EC:5.4.3.8] (A)
K00925	0.078%	HC	0.0005%	0.007	1.0	similar to acetate kinase; K00925 acetate kinase [EC:2.7.2.1] (A)
K02492	0.025%	PD	0.0042%	0.004	1.0	HEMA2; glutamyl-tRNA reductase 2; K02492 glutamyl-tRNA reductase [EC:1.2.1.70] (A)
K09812	0.073%	HC	-0.0057%	0.005	1.0	ftsE; predicted transporter subunit: ATP-binding component of ABC superfamily; K09812 cell division transport system ATP-binding protein (A)
K06998	0.013%	PD	0.0027%	0.007	1.0	PBLD, MAWBP, MAWDBP; phenazine biosynthesis-like protein domain containing; K06998 (A)
K02049	0.027%	PD	0.0034%	0.004	1.0	putative ABC transporter; K02049 NitT/TauT family transport system ATP-binding protein (A)
K01607	0.028%	PD	0.0030%	0.005	1.0	AO090038000029; 4-carboxymuconolactone decarboxylase; K01607 4-carboxymuconolactone decarboxylase [EC:4.1.1.44] (A)
K00537	0.011%	PD	0.0027%	0.003	1.0	arsenate reductase-like; K00537 arsenate reductase [EC:1.20.4.1] (A)
K03783	0.065%	HC	0.0004%	0.009	1.0	PNP, NP, PRO1837, PUNP; purine nucleoside phosphorylase (EC:2.4.2.1); K03783 purine-nucleoside phosphorylase [EC:2.4.2.1] (A)
K02652	0.019%	PD	0.0033%	0.004	1.0	pilus biogenesis protein; K02652 type IV pilus assembly protein PilB (A)
K02669	0.035%	PD	0.0030%	0.005	1.0	yggR; predicted pilus retraction ATPase; K02669 twitching motility protein PilT (A)
K00100	0.016%	PD	0.0029%	0.001	1.0	Akr1c6, 3alpha-HSD, Akr1c1, Hsd17b5; aldo-keto reductase family 1, member C6; K00100 [EC:1.1.1.-] (A)
K01966	0.003%	PD	0.0027%	0.005	1.0	PCCB; propionyl CoA carboxylase, beta polypeptide (EC:6.4.1.3); K01966 propionyl-CoA carboxylase beta chain [EC:6.4.1.3] (A)
K01447	0.007%	PD	0.0060%	0.007	1.0	N-acetylmuramoyl-L-alanine amidase; K01447 N-acetylmuramoyl-L-alanine amidase [EC:3.5.1.28] (A)
K00955	0.008%	PD	0.0018%	0.001	1.0	nodQ; bifunctional sulfate adenylyltransferase/adenylyl-sulfate kinase (EC:2.7.1.25 2.7.7.4); K00955 bifunctional enzyme CysN/CysC [EC:2.7.7.4 2.7.1.25] (A)
K13542	0.020%	PD	0.0035%	0.006	1.0	hemD; uroporphyrinogen III C2,C7-methyltransferase and uroporphyrinogen III synthase; K13542 uroporphyrinogen III methyltransferase / synthase [EC:2.1.1.107 4.2.1.75] (A)
K07192	0.018%	PD	0.0008%	0.009	1.0	FLOT1; flotillin 1; K07192 flotillin (A)
K09940	0.002%	PD	0.0028%	0.009	1.0	hypothetical protein; K09940 hypothetical protein (A)
K03818	0.002%	PD	0.0026%	0.007	1.0	wcaF; predicted acyl transferase; K03818 putative colanic acid biosynthesis acetyltransferase WcaF [EC:2.3.1.-] (A)
K16870	0.000%	PD	0.0000%	0.007	1.0	N-acetylglucosaminyl-diphospho-decaprenol L-rhamnosyltransferase [EC:2.4.1.289]

comparison iRBD versus HC

KEGG orthologous group	HC mean % abundan	greater median in	estimate of location parameter	P-Value (Wilcoxon rank sum test)	adjusted P-value	KEGG description
K02863	0.080%	HC	-0.0022%	0.009	0.4	PRPL1; Plastid ribosomal protein L1 large ribosomal subunit; K02863 large subunit ribosomal protein L1 (A)
K02906	0.080%	HC	-0.0022%	0.009	0.4	50S ribosomal protein L3-1; K02906 large subunit ribosomal protein L3 (A)
K02933	0.072%	HC	-0.0014%	0.007	0.4	emb2394; 50S ribosomal protein L6; K02933 large subunit ribosomal protein L6 (A)
K02950	0.080%	HC	-0.0022%	0.008	0.4	rps12; ribosomal protein S12; K02950 small subunit ribosomal protein S12 (A)
K02967	0.079%	HC	-0.0024%	0.010	0.4	rps2; 30S ribosomal protein S2; K02967 small subunit ribosomal protein S2 (A)
K02996	0.061%	HC	-0.0056%	0.010	0.4	RPS9; 30S ribosomal protein S9; K02996 small subunit ribosomal protein S9 (A)
K01533	0.080%	HC	-0.0008%	0.008	0.4	ATP7A, DSMAX, MK, MNK, SMAX3; ATPase, Cu++ transporting, alpha polypeptide (EC:3.6.3.4); K01533 Cu2+-exporting ATPase [EC:3.6.3.4] (A)
K00600	0.076%	HC	-0.0036%	0.009	0.4	SHMT1, CSHMT, SHMT; serine hydroxymethyltransferase 1 (soluble) (EC:2.1.2.1); K00600 glycine hydroxymethyltransferase [EC:2.1.2.1] (A)
K00820	0.082%	HC	-0.0031%	0.005	0.4	GFPT1, GFA, GFAT, GFAT_1, GFAT1, GFAT1m, GFPT, GFPT1L, MSLG; glutamine--fructose-6-phosphate transaminase 1 (EC:2.6.1.16); K00820 glucosamine--fructose-6-phosphate aminotransferase (isomerizing) [EC:2.6.1.16] (A)
K04077	0.082%	HC	-0.0027%	0.008	0.4	HSPD1, CPN60, GROEL, HLD4, HSP-60, HSP60, HSP65, HuCHA60, SPG13; heat shock 60kDa protein 1 (chaperonin); K04077 chaperonin GroEL (A)
K03168	0.073%	HC	-0.0005%	0.007	0.4	DNA topoisomerase-like protein (EC:5.99.1.2); K03168 DNA topoisomerase I [EC:5.99.1.2] (A)
K00609	0.080%	HC	-0.0026%	0.007	0.4	PYRB; aspartate carbamoyltransferase; K00609 aspartate carbamoyltransferase catalytic subunit [EC:2.1.3.2] (A)
K04078	0.082%	HC	-0.0026%	0.003	0.3	HSPE1, CPN10, EPF, GROES, HSP10; heat shock 10kDa protein 1 (chaperonin 10); K04078 chaperonin GroES (A)
K00602	0.084%	HC	-0.0037%	0.003	0.3	ATIC, AICAR, AICARFT, IMPCHASE, PURH; 5-aminoimidazole-4-carboxamide ribonucleotide formyltransferase/IMP cyclohydrolase (EC:2.1.2.3 3.5.4.10); K00602 phosphoribosylaminoimidazolecarboxamide formyltransferase / IMP cyclohydrolase [EC:2.1.2.3 3.5.4.10] (A)
K02887	0.080%	HC	-0.0023%	0.010	0.4	rpl20; 50S ribosomal protein L20; K02887 large subunit ribosomal protein L20 (A)
K02355	0.127%	HC	-0.0047%	0.009	0.4	GFM2, EF-G2mt, EFG2, MRRF2, MST027, RRF2, RRF2mt, hEFG2, mEF-G_2; G elongation factor, mitochondrial 2; K02355 elongation factor G (A)
K02358	0.084%	HC	-0.0033%	0.004	0.4	TUFM, COXPD4, EF-TuMT, EFTU, P43; Tu translation elongation factor, mitochondrial; K02358 elongation factor Tu (A)
K02926	0.079%	HC	-0.0023%	0.010	0.4	RPL4; 50S ribosomal protein L4; K02926 large subunit ribosomal protein L4 (A)
K03086	0.131%	HC	-0.0141%	0.001	0.3	SIGA; sigma factor A; K03086 RNA polymerase primary sigma factor (A)
K03977	0.079%	HC	-0.0023%	0.009	0.4	GTPase Der-like; K03977 GTP-binding protein (A)
K01924	0.081%	HC	-0.0021%	0.004	0.3	murC; UDP-N-acetylmuramate:L-alanine ligase (EC:6.3.2.8); K01924 UDP-N-acetylmuramate--alanine ligase [EC:6.3.2.8] (A)
K03723	0.081%	HC	-0.0023%	0.004	0.3	mfd; transcription-repair coupling factor; K03723 transcription-repair coupling factor (superfamily II helicase) [EC:3.6.4.-] (A)
K06997	0.080%	HC	-0.0029%	0.007	0.4	PROSC; proline synthetase co-transcribed homolog (bacterial); K06997 (A)
K02907	0.078%	HC	-0.0010%	0.007	0.4	large subunit ribosomal protein L30; K02907 large subunit ribosomal protein L30 (A)
K02860	0.081%	HC	-0.0024%	0.004	0.3	RimM-like protein; K02860 16S rRNA processing protein RimM (A)
K00817	0.082%	HC	-0.0035%	0.002	0.3	HISN6B; Histidinol-phosphate aminotransferase; K00817 histidinol-phosphate aminotransferase [EC:2.6.1.9] (A)
K01262	0.089%	HC	-0.0023%	0.001	0.3	XPNPEP3, APP3, NPHPL1; X-prolyl aminopeptidase (aminopeptidase P) 3, putative (EC:3.4.11.9); K01262 Xaa-Pro aminopeptidase [EC:3.4.11.9] (A)
K06287	0.082%	HC	-0.0027%	0.004	0.4	1810009N02Rik; RIKEN cDNA 1810009N02 gene; K06287 septum formation protein (A)
K02013	0.144%	RBD	0.0055%	0.003	0.3	fhuC; iron-hydroxamate transporter subunit; K02013 iron complex transport system ATP-binding protein [EC:3.6.3.34] (A)
K06966	0.049%	HC	-0.0082%	0.004	0.4	LOG1; cytokinin riboside 5'-monophosphate phosphoribohydrolase LOG1; K06966 (A)
K02015	0.189%	RBD	0.0057%	0.003	0.3	fhuB; fused iron-hydroxamate transporter subunits of ABC superfamily: membrane components; K02015 iron complex transport system permease protein (A)
K00645	0.082%	HC	-0.0029%	0.006	0.4	MCAT, FASN2C, MCT, MT, NET62, fabD; malonyl CoA:ACP acyltransferase (mitochondrial) (EC:2.3.1.39); K00645 [acyl-carrier-protein] S-malonyltransferase [EC:2.3.1.39] (A)
K04485	0.075%	HC	-0.0036%	0.003	0.3	DNA repair protein RadA homolog; K04485 DNA repair protein RadA/Sms (A)
K01776	0.066%	HC	-0.0046%	0.003	0.3	murI; glutamate racemase (EC:5.1.1.3); K01776 glutamate racemase [EC:5.1.1.3] (A)
K01652	0.115%	RBD	0.0053%	0.006	0.4	similar to 2-hydroxyacyl-CoA lyase 1; K01652 acetolactate synthase I/II/III large subunit [EC:2.2.1.6] (A)
K00648	0.083%	HC	-0.0052%	0.004	0.3	KAS_III; 3-oxoacyl-[acyl-carrier-protein] synthase III; K00648 3-oxoacyl-[acyl-carrier-protein] synthase III [EC:2.3.1.180] (A)
K01778	0.090%	RBD	0.0072%	0.009	0.4	diaminopimelate epimerase; K01778 diaminopimelate epimerase [EC:5.1.1.7] (A)
K00640	0.110%	HC	-0.0051%	0.003	0.3	SERAT2;1; serine O-acetyltransferase; K00640 serine O-acetyltransferase [EC:2.3.1.30] (A)
K01662	0.088%	HC	-0.0054%	0.001	0.3	DXPS1; 1-deoxy-D-xylulose 5-phosphate synthase 1; K01662 1-deoxy-D-xylulose-5-phosphate synthase [EC:2.2.1.7] (A)

K00759	0.058%	HC	-0.0068%	0.003	0.3	APRT, AMP; adenine phosphoribosyltransferase (EC:2.4.2.7); K00759 adenine phosphoribosyltransferase [EC:2.4.2.7] (A)
K01897	0.108%	HC	-0.0108%	0.008	0.4	ACSL1, ACS1, FACL1, FACL2, LACS, LACS1, LACS2; acyl-CoA synthetase long-chain family member 1 (EC:6.2.1.3); K01897 long-chain acyl-CoA synthetase [EC:6.2.1.3] (A)
K00616	0.050%	RBD	0.0038%	0.006	0.4	TALDO1, TAL, TAL-H, TALDOR, TALH; transaldolase 1 (EC:2.2.1.2); K00616 transaldolase [EC:2.2.1.2] (A)
K00950	0.023%	HC	-0.0050%	0.010	0.4	DHPS, PPPK; dihydropteroate synthetase (EC:2.5.1.15); K00796 dihydropteroate synthase [EC:2.5.1.15] K00950 2-amino-4-hydroxy-6-hydroxymethyldihydropteridine diphosphokinase [EC:2.7.6.3] (A)
K07560	0.071%	HC	-0.0037%	0.005	0.4	DTD1, C20orf88, DUEB, HARS2, bA379J5.3, bA555E18.1, pqn-68; D-tyrosyl-tRNA deacylase 1 homolog (S. cerevisiae) (EC:6.1.1.21); K07560 D-tyrosyl-tRNA(Tyr) deacylase [EC:3.1.-.-] (A)
K07567	0.075%	HC	-0.0015%	0.009	0.4	endoribonuclease L-PSP, putative; K07567 TdcF protein (A)
K01752	0.122%	RBD	0.0076%	0.007	0.4	SDS, SDH; serine dehydratase (EC:4.3.1.17 4.3.1.19); K01752 L-serine dehydratase [EC:4.3.1.17] (A)
K00831	0.078%	HC	-0.0021%	0.006	0.4	PSAT1, EPIP, PSA, PSAT; phosphoserine aminotransferase 1 (EC:2.6.1.52); K00831 phosphoserine aminotransferase [EC:2.6.1.52] (A)
K03786	0.047%	HC	-0.0084%	0.009	0.4	DEHA2F23584p; K03786 3-dehydroquinate dehydratase II [EC:4.2.1.10] (A)
K01953	0.105%	RBD	0.0049%	0.003	0.3	ASNS, TS11; asparagine synthetase (glutamine-hydrolyzing) (EC:6.3.5.4); K01953 asparagine synthase (glutamine-hydrolysing) [EC:6.3.5.4] (A)
K02687	0.077%	HC	-0.0030%	0.004	0.4	prmA; methyltransferase for 50S ribosomal subunit protein L11 (EC:2.1.1.-); K02687 ribosomal protein L11 methyltransferase [EC:2.1.1.-] (A)
K03978	0.080%	HC	-0.0025%	0.004	0.3	GTP-binding protein At2g22870-like; K03978 GTP-binding protein (A)
K03570	0.078%	HC	-0.0024%	0.001	0.3	mreC; cell wall structural complex MreBCD transmembrane component MreC; K03570 rod shape-determining protein MreC (A)
K02970	0.079%	HC	-0.0025%	0.009	0.4	rpsU; 30S ribosomal subunit protein S21; K02970 small subunit ribosomal protein S21 (A)
K01719	0.013%	HC	-0.0052%	0.006	0.4	UROS, UROIIIS; uroporphyrinogen III synthase (EC:4.2.1.75); K01719 uroporphyrinogen-III synthase [EC:4.2.1.75] (A)
K02837	0.064%	HC	-0.0055%	0.003	0.3	prfC; peptide chain release factor RF-3; K02837 peptide chain release factor 3 (A)
K07636	0.077%	HC	-0.0005%	0.009	0.4	phoR; sensory histidine kinase in two-component regulatory system with PhoB (EC:2.7.3.-); K07636 two-component system, OmpR family, phosphate regulon sensor histidine kinase PhoR [EC:2.7.13.3] (A)
K06950	0.129%	HC	-0.0104%	0.006	0.4	Metal-dependent phosphohydrolase; K06950 uncharacterized protein (A)
K09760	0.042%	HC	-0.0060%	0.008	0.4	rmuC; predicted recombination limiting protein; K09760 DNA recombination protein RmuC (A)
K01187	0.099%	RBD	0.0098%	0.008	0.4	sucrase-isomaltase, intestinal-like; K01187 alpha-glucosidase [EC:3.2.1.20] (A)
K03699	0.047%	RBD	0.0067%	0.004	0.3	tlyC; hemolysin; K03699 putative hemolysin (A)
K05366	0.079%	HC	-0.0082%	0.000	0.3	mrcA; fused penicillin-binding protein 1a: murein transglycosylase/murein transpeptidase (EC:2.4.2.- 3.4.-.-); K05366 penicillin-binding protein 1A [EC:2.4.1.- 3.4.-.-] (A)
K01433	0.006%	HC	-0.0011%	0.003	0.3	Formyl transferase; K01433 formyltetrahydrofolate deformylase [EC:3.5.1.10] (A)
K07053	0.105%	RBD	0.0053%	0.003	0.3	Polymerase/histidinol phosphatase-like protein; K07053 (A)
K03547	0.045%	RBD	0.0081%	0.006	0.4	sbcD; exonuclease, dsDNA, ATP-dependent; K03547 exonuclease SbcD (A)
K03294	0.025%	RBD	0.0036%	0.005	0.4	uncharacterized LOC100776671; K03294 basic amino acid/polyamine antiporter, APA family (A)
K03581	0.045%	RBD	0.0040%	0.008	0.4	recD; exonuclease V (RecBCD complex), alpha chain (EC:3.1.11.5); K03581 exodeoxyribonuclease V alpha subunit [EC:3.1.11.5] (A)
K06890	0.034%	HC	-0.0084%	0.004	0.4	GRINA, HNRGW, LFG1, NMDARA1, TMBIM3; glutamate receptor, ionotropic, N-methyl D-aspartate-associated protein 1 (glutamate binding); K06890 (A)
K01259	0.004%	HC	-0.0013%	0.002	0.3	PIP; proline iminopeptidase; K01259 proline iminopeptidase [EC:3.4.11.5] (A)
K09125	0.011%	HC	-0.0053%	0.005	0.4	yhhQ; inner membrane protein, DUF165 family; K09125 hypothetical protein (A)
K07054	0.004%	HC	-0.0027%	0.000	0.3	ypfJ; conserved protein; K07054 (A)
K05794	0.003%	HC	-0.0006%	0.000	0.3	terC; phage inhibition, colicin resistance and tellurite resistance protein; K05794 tellurite resistance protein TerC (A)
K03316	0.004%	HC	-0.0006%	0.004	0.4	CPA1 family transporter: sodium ion/proton; K03316 monovalent cation:H+ antiporter, CPA1 family (A)
K01151	0.047%	RBD	0.0043%	0.001	0.3	DNA LYASE/ENDONUCLEASE 4; K01151 deoxyribonuclease IV [EC:3.1.21.2] (A)
K00782	0.009%	HC	-0.0036%	0.006	0.4	ykgG; predicted transporter; K00782 hypothetical protein (A)
K07727	0.042%	RBD	0.0034%	0.007	0.4	XRE family transcriptional regulator; K07727 putative transcriptional regulator (A)
K05521	0.016%	HC	-0.0071%	0.003	0.3	yegU; predicted hydrolase; K05521 ADP-ribosylglycohydrolase [EC:3.2.-.-] (A)
K03549	0.005%	HC	-0.0007%	0.005	0.4	KT2; potassium transporter 2; K03549 KUP system potassium uptake protein (A)
K08281	0.005%	HC	-0.0040%	0.000	0.3	pncA; nicotinamidase/pyrazinamidase (EC:3.5.1.19 3.5.1.-); K08281 nicotinamidase/pyrazinamidase [EC:3.5.1.19 3.5.1.-] (A)
K03929	0.012%	HC	-0.0009%	0.008	0.4	putative esterase; K03929 para-nitrobenzyl esterase [EC:3.1.1.-] (A)
K01654	0.022%	RBD	0.0029%	0.007	0.4	neuB; sialic acid synthase NeuB (EC:1.2.4.1); K01654 N-acetylneuraminate synthase [EC:2.5.1.56] (A)
K06177	0.010%	HC	-0.0053%	0.003	0.3	rluA; pseudouridine synthase for 23S rRNA (position 746) and tRNAphe(position 32) (EC:4.2.1.70); K06177 tRNA pseudouridine32 synthase / 23S rRNA pseudouridine746 synthase [EC:5.4.99.28 5.4.99.29] (A)
K07259	0.012%	HC	-0.0065%	0.001	0.3	dacB; D-alanyl-D-alanine carboxypeptidase (EC:3.4.16.4); K07259 D-alanyl-D-alanine carboxypeptidase / D-alanyl-D-alanine-endopeptidase (penicillin-binding protein 4) [EC:3.4.16.4 3.4.21.-] (A)
K08963	0.028%	RBD	0.0050%	0.008	0.4	MRI1, MTNA, Ypr118w; methylthioribose-1-phosphate isomerase homolog (S. cerevisiae) (EC:5.3.1.23); K08963 methylthioribose-1-phosphate isomerase [EC:5.3.1.23] (A)
K04651	0.036%	RBD	0.0037%	0.004	0.4	hypA; protein involved in nickel insertion into hydrogenases 3; K04651 hydrogenase nickel incorporation protein HypA/HybF (A)
K04652	0.034%	RBD	0.0038%	0.003	0.3	hypB; GTP hydrolase involved in nickel liganding into hydrogenases; K04652 hydrogenase nickel incorporation protein HypB (A)
K01417	0.009%	RBD	0.0023%	0.007	0.4	ATP-dependent Zn protease FtsH2; K01417 [EC:3.4.24.-] (A)

K07023	0.036%	RBD	0.0039%	0.010	0.4	HDDC2, C6orf74, NS5ATP2, dJ167O5.2; HD domain containing 2; K07023 putative hydrolases of HD superfamily (A)
K07185	0.013%	RBD	0.0011%	0.003	0.3	TspO/MBR family protein; K07185 tryptophan-rich sensory protein (A)
K05516	0.011%	HC	-0.0059%	0.004	0.4	cbpA; curved DNA-binding protein, DnaJ homologue that functions as a co-chaperone of DnaK; K05516 curved DNA-binding protein (A)
K00983	0.023%	RBD	0.0033%	0.007	0.4	CMAS, CSS; cytidine monophosphate N-acetylneuraminic acid synthetase (EC:2.7.7.43); K00983 N-acylneuraminate cytidylyltransferase [EC:2.7.7.43] (A)
K10536	0.023%	RBD	0.0047%	0.008	0.4	EMB1873; agmatine deiminase; K10536 agmatine deiminase [EC:3.5.3.12] (A)
K07456	0.075%	HC	-0.0017%	0.009	0.4	DNA mismatch repair protein MutS2; K07456 DNA mismatch repair protein MutS2 (A)
K07334	0.006%	HC	-0.0017%	0.001	0.3	putative toxin-plasmid maintenance system killer protein; K07334 proteic killer suppression protein (A)
K07213	0.012%	HC	-0.0047%	0.008	0.4	ATOX1, ATX1, HAH1; ATX1 antioxidant protein 1 homolog (yeast) (EC:6.2.1.2); K07213 copper chaperone (A)
K00231	0.005%	HC	-0.0033%	0.002	0.3	PPOX, PPO, V290M, VP; protoporphyrinogen oxidase (EC:1.3.3.4); K00231 oxygen-dependent protoporphyrinogen oxidase [EC:1.3.3.4] (A)
K01734	0.099%	RBD	0.0102%	0.004	0.4	mgsA; methylglyoxal synthase (EC:4.2.3.3); K01734 methylglyoxal synthase [EC:4.2.3.3] (A)
K02007	0.029%	RBD	0.0027%	0.001	0.3	cbiM; cobalamin biosynthesis protein CbiM; K02007 cobalt/nickel transport system permease protein (A)
K02006	0.029%	RBD	0.0029%	0.001	0.3	ABC transporter ATP-binding protein; K02006 cobalt/nickel transport system ATP-binding protein (A)
K01858	0.006%	HC	-0.0007%	0.002	0.3	ISYNA1, INO1, INOS, IPS, IPS_1, IPS-1; inositol-3-phosphate synthase 1 (EC:5.5.1.4); K01858 myo-inositol-1-phosphate synthase [EC:5.5.1.4] (A)
K01442	0.059%	RBD	0.0099%	0.006	0.4	choloylglycine hydrolase; K01442 choloylglycine hydrolase [EC:3.5.1.24] (A)
K03387	0.012%	HC	-0.0033%	0.008	0.4	ahpF; alkyl hydroperoxide reductase, F52a subunit, FAD/NAD(P)-binding (EC:1.11.1.15); K03387 alkyl hydroperoxide reductase subunit F [EC:1.6.4.-] (A)
K03286	0.005%	HC	-0.0009%	0.010	0.4	ompA; outer membrane protein A (3a;II*;G;d); K03286 OmpA-OmpF porin, OOP family (A)
K02843	0.005%	HC	-0.0023%	0.006	0.4	rfaF; ADP-heptose:LPS heptosyltransferase II (EC:2.-.-.-); K02843 heptosyltransferase II [EC:2.4.-.-] (A)
K03718	0.019%	HC	-0.0016%	0.007	0.4	asnC; DNA-binding transcriptional dual regulator; K03718 Lrp/AsnC family transcriptional regulator, regulator for asnA, asnC and gidA (A)
K08987	0.002%	HC	0.0040%	0.002	0.3	integral membrane protein; K08987 putative membrane protein (A)
K01552	0.013%	RBD	0.0022%	0.007	0.4	PfATPase7; P-type ATPase, putative (EC:3.6.3.-); K01552 [EC:3.6.3.-] (A)
K04764	0.004%	HC	-0.0033%	0.001	0.3	ihfA; integration host factor (IHF), DNA-binding protein, alpha subunit; K04764 integration host factor subunit alpha (A)
K06973	0.049%	RBD	0.0091%	0.002	0.3	peptidase membrane zinc metallopeptidase; K06973 (A)
K08974	0.036%	RBD	0.0077%	0.006	0.4	membrane protein; K08974 putative membrane protein (A)
K00282	0.011%	HC	-0.0013%	0.006	0.4	glycine cleavage system protein P; K00282 glycine dehydrogenase subunit 1 [EC:1.4.4.2] (A)
K00283	0.011%	HC	-0.0013%	0.006	0.4	glycine cleavage system protein P; K00283 glycine dehydrogenase subunit 2 [EC:1.4.4.2] (A)
K07214	0.007%	HC	-0.0001%	0.006	0.4	fes; enterobactin/ferric enterobactin esterase; K07214 enterochelin esterase and related enzymes (A)
K02361	0.009%	HC	-0.0005%	0.003	0.3	entC; isochorismate synthase 1 (EC:5.4.4.2); K02361 isochorismate synthase [EC:5.4.4.2] (A)
K12960	0.029%	RBD	0.0038%	0.009	0.4	chlorohydrolase family protein; K12960 5-methylthioadenosine/S-adenosylhomocysteine deaminase [EC:3.5.4.31 3.5.4.28] (A)
K07273	0.012%	HC	-0.0003%	0.001	0.3	yegX; predicted hydrolase; K07273 lysozyme (A)
K08217	0.037%	RBD	0.0035%	0.004	0.3	macrolide-efflux protein; K08217 MFS transporter, DHA3 family, macrolide efflux protein (A)
K05801	0.010%	HC	-0.0023%	0.003	0.3	djlA; DnaJ-like protein, membrane anchored; K05801 DnaJ like chaperone protein (A)
K07284	0.011%	RBD	0.0020%	0.004	0.4	LPXTG-site transpeptidase; K07284 sortase A [EC:3.4.22.70] (A)
K07192	0.018%	RBD	0.0020%	0.009	0.4	FLOT1; flotillin 1; K07192 flotillin (A)
K07454	0.003%	HC	-0.0028%	0.001	0.3	HNH nuclease; K07454 putative restriction endonuclease (A)
K01278	0.019%	HC	-0.0066%	0.004	0.3	DPP4, ADABP, ADCP2, CD26, DPPIV, TP103; dipeptidyl-peptidase 4 (EC:3.4.14.5); K01278 dipeptidyl-peptidase 4 [EC:3.4.14.5] (A)
K09702	0.022%	RBD	0.0050%	0.007	0.4	REE1; Ree1p; K09702 hypothetical protein (A)
K01185	0.003%	HC	-0.0063%	0.001	0.3	LYZL2; lysozyme-like 2 (EC:3.2.1.17); K01185 lysozyme [EC:3.2.1.17] (A)
K03773	0.012%	HC	-0.0022%	0.008	0.4	FBP11-1; peptidyl-prolyl cis-trans isomerase, FKBP-type; K03773 FKBP-type peptidyl-prolyl cis-trans isomerase FklB [EC:5.2.1.8] (A)
K07979	0.032%	RBD	0.0042%	0.009	0.4	GntR family transcriptional regulator; K07979 GntR family transcriptional regulator (A)
K07218	0.001%	HC	0.0016%	0.000	0.3	NosD, nitrous oxidase accessory protein; K07218 nitrous oxidase accessory protein (A)
K03816	0.060%	HC	-0.0028%	0.006	0.4	xanthine phosphoribosyltransferase (EC:2.4.2.22); K03816 xanthine phosphoribosyltransferase [EC:2.4.2.22] (A)
K06990	0.002%	HC	0.0020%	0.003	0.3	MEMO1, C2orf4, MEMO, NS5ATP7; mediator of cell motility 1; K06990 (A)
K00376	0.001%	HC	0.0016%	0.000	0.3	nitrous-oxide reductase (EC:1.7.99.6); K00376 nitrous-oxide reductase [EC:1.7.2.4] (A)
K13626	0.022%	RBD	0.0026%	0.010	0.4	flagellar assembly protein FliW; K13626 flagellar assembly factor FliW (A)
K05364	0.012%	RBD	0.0005%	0.005	0.4	peptidoglycan glycosyltransferase (EC:2.4.1.129); K05364 peptidoglycan glycosyltransferase [EC:2.4.1.129] (A)
K16922	0.002%	HC	-0.0047%	0.000	0.3	putative peptide zinc metalloprotease protein
K08984	0.002%	HC	0.0020%	0.000	0.3	yjdF; conserved inner membrane protein; K08984 putative membrane protein (A)
K05499	0.001%	HC	-0.0044%	0.001	0.3	cytR; DNA-binding transcriptional dual regulator; K05499 LacI family transcriptional regulator, repressor for deo operon, udp, cdd, tsx, nupC, and nupG (A)
K07000	0.003%	HC	-0.0014%	0.006	0.4	ycfP; conserved protein; K07000 (A)
K03385	0.008%	HC	-0.0051%	0.008	0.4	nrfA; nitrite reductase, formate-dependent, cytochrome; K03385 cytochrome c-552 [EC:1.7.2.2] (A)

K00096	0.025%	RBD	0.0020%	0.007	0.4	glycerol dehydrogenase; K00096 glycerol-1-phosphate dehydrogenase [NAD(P)] [EC:1.1.1.261] (A)
K00690	0.013%	RBD	0.0007%	0.004	0.3	ycjM; predicted glucosyltransferase; K00690 sucrose phosphorylase [EC:2.4.1.7] (A)
K17318	0.137%	RBD	0.0217%	0.008	0.4	putative aldouronate transport system substrate-binding protein
K17319	0.101%	RBD	0.0146%	0.007	0.4	putative aldouronate transport system permease protein
K17320	0.101%	RBD	0.0146%	0.007	0.4	putative aldouronate transport system permease protein
K09155	0.013%	HC	-0.0047%	0.005	0.4	PAS/PAC sensor protein; K09155 hypothetical protein (A)
K07079	0.068%	RBD	0.0085%	0.006	0.4	aldo/keto reductase; K07079 (A)
K01713	0.002%	HC	0.0023%	0.002	0.3	cyclohexadienyl dehydratase; K01713 cyclohexadienyl dehydratase [EC:4.2.1.51 4.2.1.91] (A)
K02385	0.020%	RBD	0.0025%	0.007	0.4	flbD; flagellar protein FlbD; K02385 flagellar protein FlbD (A)
K08364	0.001%	HC	0.0014%	0.002	0.3	mercuric transport protein periplasmic componentprecursor MerP; K08364 periplasmic mercuric ion binding protein (A)
K09474	0.006%	HC	-0.0028%	0.001	0.3	acid phosphatase; K09474 acid phosphatase (class A) [EC:3.1.3.2] (A)
K15876	0.006%	HC	-0.0052%	0.004	0.3	NRFH; small subunit of cytochrome c nitrite reductase; K15876 cytochrome c-type protein (A)
K03716	0.014%	RBD	0.0001%	0.010	0.4	DNA repair photolyase; K03716 spore photoproduct lyase [EC:4.1.99.14] (A)
K05792	0.001%	HC	-0.0045%	0.002	0.3	terA; phage inhibition, colicin resistance and tellurite resistance protein; K05792 tellurite resistance protein TerA (A)
K05791	0.001%	HC	0.0014%	0.001	0.3	terZ; phage inhibition, colicin resistance and tellurite resistance protein; K05791 tellurium resistance protein TerZ (A)
K07791	0.006%	HC	-0.0027%	0.008	0.4	dcuA; C4-dicarboxylate antiporter; K07791 anaerobic C4-dicarboxylate transporter DcuA (A)
K07792	0.003%	HC	-0.0032%	0.008	0.4	dcuB; C4-dicarboxylate transporter, anaerobic; DcuS co-sensor; K07792 anaerobic C4 dicarboxylate transporter DcuB (A)
K00113	0.002%	HC	0.0023%	0.001	0.3	glpC; anaerobic sn-glycerol-3-phosphate dehydrogenase, C subunit, 4Fe-4S iron-sulfur cluster; K00113 glycerol-3-phosphate dehydrogenase subunit C [EC:1.1.5.3] (A)
K00895	0.008%	HC	-0.0007%	0.004	0.3	pyrophosphate--fructose-6-phosphate 1-phosphotransferase; K00895 pyrophosphate--fructose-6-phosphate 1-phosphotransferase [EC:2.7.1.90] (A)
K00112	0.002%	HC	0.0023%	0.001	0.3	glpB; sn-glycerol-3-phosphate dehydrogenase (anaerobic), membrane anchor subunit (EC:1.1.5.3); K00112 glycerol-3-phosphate dehydrogenase subunit B [EC:1.1.5.3] (A)
K11921	0.003%	HC	-0.0037%	0.003	0.3	cynR; transcriptional activator of cyn operon; autorepressor; K11921 LysR family transcriptional regulator (A)
K07014	0.002%	HC	-0.0031%	0.001	0.3	yejM; predicted hydrolase, inner membrane; K07014 (A)
K09805	0.002%	HC	-0.0048%	0.000	0.3	hypothetical protein; K09805 hypothetical protein (A)
K07720	0.188%	RBD	0.0212%	0.008	0.4	yesN; two-component response regulator YesM; K07720 two-component system, response regulator YesN (A)
K03810	0.001%	HC	-0.0043%	0.001	0.3	yceM; predicted oxidoreductase with NAD(P)-binding Rossmann-fold domain; K03810 virulence factor (A)
K07718	0.186%	RBD	0.0218%	0.008	0.4	yesM; two-component sensor histidine kinase YesN; K07718 two-component system, sensor histidine kinase YesM [EC:2.7.13.3] (A)
K06610	0.001%	HC	-0.0044%	0.002	0.3	major facilitator superfamily protein; K06610 MFS transporter, SP family, inositol transporter (A)
K11031	0.002%	HC	0.0023%	0.006	0.4	Thiol-activated cytolysin; K11031 thiol-activated cytolysin (A)
K06374	0.023%	RBD	0.0026%	0.009	0.4	spmB; spore maturation protein B; K06374 spore maturation protein B (A)
K06373	0.029%	RBD	0.0047%	0.009	0.4	spore maturation protein A; K06373 spore maturation protein A (A)
K01085	0.003%	HC	-0.0044%	0.001	0.3	agp; glucose-1-phosphatase/inositol phosphatase (EC:3.1.3.10); K01085 glucose-1-phosphatase [EC:3.1.3.10] (A)
K04835	0.001%	HC	0.0017%	0.001	0.3	methylaspartate ammonia-lyase C-terminal domain protein; K04835 methylaspartate ammonia-lyase [EC:4.3.1.2] (A)
K12962	0.001%	HC	-0.0044%	0.003	0.3	arnE; undecaprenyl phosphate-alpha-L-ara4N exporter; flippase ArnEF subunit; K12962 undecaprenyl phosphate-alpha-L-ara4N flippase subunit ArnE (A)
K09680	0.013%	HC	-0.0015%	0.004	0.3	PANK1, PANK; pantothenate kinase 1 (EC:2.7.1.33); K09680 type II pantothenate kinase [EC:2.7.1.33] (A)
K03146	0.002%	HC	-0.0029%	0.003	0.3	THI1; thiazole biosynthetic enzyme; K03146 thiamine biosynthetic enzyme (A)
K10797	0.010%	RBD	0.0015%	0.008	0.4	NADH:flavin oxidoreductase; K10797 2-enoate reductase [EC:1.3.1.31] (A)
K15974	0.001%	HC	0.0014%	0.003	0.3	mprA; DNA-binding transcriptional repressor of microcin B17 synthesis and multidrug efflux; K15974 MarR family transcriptional regulator, negative regulator of the multidrug operon emrRAB (A)
K15855	0.001%	HC	-0.0044%	0.001	0.3	similar to exo-beta-D-glucosaminidase; K15855 exo-1,4-beta-D-glucosaminidase [EC:3.2.1.165] (A)
K03289	0.005%	HC	-0.0026%	0.001	0.3	nupG; nucleoside transporter; K03289 MFS transporter, NHS family, nucleoside permease (A)

ID	analyzed samples	comment	Sample ID	extraction batch	sequencing run	sequencing coordinate	library PCR yield	qPCR human DNA	qPCR bacterial yield	fastq Files
			nose							
HC001	nose		HC001.n	L2.n	P5	D06	3.5	0.01	0.24	L-106-HC001-P5-D06_S42_L001_R1_001.fastq.gz,L-106-HC001-P5-D06_S42_L001_R2_001.fastq.gz
HC002	nose & stool		HC002.n	D2.n	P4	D03	6.49	0	0	D03-D-103-HC002_S39_L001_R1_001.fastq.gz,D03-D-103-HC002_S39_L001_R2_001.fastq.gz
HC003	stool	too few reads in 18S stool								
HC004	nose		HC004.n	D2.n	P4	D02	10.63	0.03	0.38	D02-D-102-HC004_S38_L001_R1_001.fastq.gz,D02-D-102-HC004_S38_L001_R2_001.fastq.gz
HC005	nose		HC005.n	A2.n	P4	A04	4.29	0.01	0.68	A04-A-104-HC005_S4_L001_R1_001.fastq.gz,A04-A-104-HC005_S4_L001_R2_001.fastq.gz
HC006	nose		HC006.n	F2.n	P4	F10	3.58	0	0.04	F10-F-110-HC006_S70_L001_R1_001.fastq.gz,F10-F-110-HC006_S70_L001_R2_001.fastq.gz
HC007	nose & stool		HC007.n	D1.n	P2	D11		0.21	2.01	D11-HC007-09b_S121_L001_R1_001.fastq,D11-HC007-09b_S121_L001_R2_001.fastq
HC008	nose		HC008.n	O2.n	P3	B01	7.82	0.01	0.04	O-101-HC008-945-3-16S_S13_L001_R1_001.fastq.gz,O-101-HC008-945-3-16S_S13_L001_R2_001.fastq.gz
HC009	nose & stool		HC009.n	E1.n	P2	E10		0.06	0.12	E10-HC009-09b_S132_L001_R1_001.fastq,E10-HC009-09b_S132_L001_R2_001.fastq
HC010	nose		HC010.n	I2.n	P5	B07	9.7	0	0.13	I-107-HC010-P5-B07_S19_L001_R1_001.fastq.gz,I-107-HC010-P5-B07_S19_L001_R2_001.fastq.gz
HC011	nose & stool		HC011.n	N2.n	P3	A01	16.27	0.07	1.79	N-101-HC011-1294-3-16S_S1_L001_R1_001.fastq.gz,N-101-HC011-1294-3-16S_S1_L001_R2_001.fastq.gz
HC012	nose		HC012.n	O2.n	P3	B03	3.01	0.01	0.52	O-103-HC012-1161-3-16S_S15_L001_R1_001.fastq.gz,O-103-HC012-1161-3-16S_S15_L001_R2_001.fastq.gz
HC013	nose & stool		HC013.n	M2.n	P5	E05	14.7	0	0.88	M-105-HC013-P5-E05_S53_L001_R1_001.fastq.gz,M-105-HC013-P5-E05_S53_L001_R2_001.fastq.gz
HC014	nose		HC014.n	L2.n	P5	D04	35.5	0.01	0.08	L-104-HC014-P5-D04_S40_L001_R1_001.fastq.gz,L-104-HC014-P5-D04_S40_L001_R2_001.fastq.gz
HC015	stool	too few reads in 18S stool								
HC016	nose & stool		HC016.n	A2.n	P4	A05	14.91	0.03	4.02	A05-A-105-HC016_S5_L001_R1_001.fastq.gz,A05-A-105-HC016_S5_L001_R2_001.fastq.gz
HC017	nose & stool		HC017.n	O2.n	P3	B12	21.82	0.01	0	O-112-HC017-1164-3-16S_S24_L001_R1_001.fastq.gz,O-112-HC017-1164-3-16S_S24_L001_R2_001.fastq.gz
HC018	nose		HC018.n	H2.n	P9	B10	76.7	0	0.05	H-110-HC018-P9-B10_S94_L001_R1_001.fastq.gz,H-110-HC018-P9-B10_S94_L001_R2_001.fastq.gz
HC019	nose & stool		HC019.n	E2.n	P4	E11	6.53	0.01	0	E11-E-111-HC019_S59_L001_R1_001.fastq.gz,E11-E-111-HC019_S59_L001_R2_001.fastq.gz
HC020	nose & stool		HC020.n	P2.n	P3	C12	2.82	0	0.27	P-112-HC020-1228-3-16S_S36_L001_R1_001.fastq.gz,P-112-HC020-1228-3-16S_S36_L001_R2_001.fastq.gz
HC021	nose		HC021.n	L2.n	P5	D03	11.9	0.01	0.82	L-103-HC021-P5-D03_S39_L001_R1_001.fastq.gz,L-103-HC021-P5-D03_S39_L001_R2_001.fastq.gz
HC022	nose		HC022.n	L2.n	P5	D07	4.4	0.03	3.85	L-107-HC022-P5-D07_S43_L001_R1_001.fastq.gz,L-107-HC022-P5-D07_S43_L001_R2_001.fastq.gz
HC023	stool									
HC024	nose & stool		HC024.n	H2.n	P9	B08	114.6	0	2.59	H-108-HC024-P9-B08_S92_L001_R1_001.fastq.gz,H-108-HC024-P9-B08_S92_L001_R2_001.fastq.gz
HC025	nose & stool		HC025.n	E2.n	P4	E10	10.37	0.01	0.18	E10-E-110-HC025_S58_L001_R1_001.fastq.gz,E10-E-110-HC025_S58_L001_R2_001.fastq.gz
HC026	nose		HC026.n	H2.n	P9	B07	37.3	0.01	0	H-107-HC026-P9-B07_S91_L001_R1_001.fastq.gz,H-107-HC026-P9-B07_S91_L001_R2_001.fastq.gz
HC027	nose & stool		HC027.n	T2.n	P9	A12	39.4	0.01	0.06	T-112-HC027-P9-A12_S84_L001_R1_001.fastq.gz,T-112-HC027-P9-A12_S84_L001_R2_001.fastq.gz
HC028	nose & stool		HC028.n	B2.n	P4	B02	20.98	0.01	2.35	B02-B-102-HC028_S14_L001_R1_001.fastq.gz,B02-B-102-HC028_S14_L001_R2_001.fastq.gz
HC029	stool									
HC030	nose		HC030.n	E2.n	P4	E01	10.7	0.01	0.07	E01-E-101-HC030_S49_L001_R1_001.fastq.gz,E01-E-101-HC030_S49_L001_R2_001.fastq.gz
HC031	nose		HC031.n	C2.n	P4	C01	5.03	0.01	0.12	C01-C-101-HC031_S25_L001_R1_001.fastq.gz,C01-C-101-HC031_S25_L001_R2_001.fastq.gz
HC032	stool									
HC033	nose & stool		HC033.n	Q2.n	P3	D01	4.23	0.02	0.14	Q-101-HC033-2060-3-16S_S37_L001_R1_001.fastq.gz,Q-101-HC033-2060-3-16S_S37_L001_R2_001.fastq.gz
HC034	stool									
HC035	stool									
HC037	nose & stool		HC037.n	G2.n	P4	G11	3.69	0	0.02	G11-G-111-HC037_S83_L001_R1_001.fastq.gz,G11-G-111-HC037_S83_L001_R1_001.fastq.gz
HC038	nose		HC038.n	J2.n	P5	A07	107.4	0.02	1.03	J-107-HC038-P5-A07_S7_L001_R1_001.fastq.gz,J-107-HC038-P5-A07_S7_L001_R2_001.fastq.gz
HC039	nose		HC039.n	B2.n	P4	B05	4.67	0	0.1	B05-B-105-HC039_S17_L001_R1_001.fastq.gz,B05-B-105-HC039_S17_L001_R2_001.fastq.gz
HC040	nose & stool		HC040.n	D1.n	P2	D08		0.04	0.12	D08-HC040-09b_S118_L001_R1_001.fastq,D08-HC040-09b_S118_L001_R2_001.fastq
HC041	nose		HC041.n	E2.n	P4	E12	5.59	0	0	E12-E-112-HC041_S60_L001_R1_001.fastq.gz,E12-E-112-HC041_S60_L001_R2_001.fastq.gz
HC042	stool									
HC043	nose		HC043.n	G2.n	P4	G10	4.16	0	0.05	G10-G-110-HC043_S82_L001_R1_001.fastq.gz,G10-G-110-HC043_S82_L001_R2_001.fastq.gz
HC044	nose & stool		HC044.n	P2.n	P3	C02	11.14	0	0	P-102-HC044-1286-3-16S_S26_L001_R1_001.fastq.gz,P-102-HC044-1286-3-16S_S26_L001_R2_001.fastq.gz
HC045	nose		HC045.n	I2.n	P5	B08	6.5	0	0.25	I-108-HC045-P5-B08_S20_L001_R1_001.fastq.gz,I-108-HC045-P5-B08_S20_L001_R2_001.fastq.gz
HC046	nose & stool		HC046.n	A2.n	P4	A03	0.9	0	0	A03-A-103-HC046_S3_L001_R1_001.fastq.gz,A03-A-103-HC046_S3_L001_R2_001.fastq.gz
HC047	nose & stool	too few reads in 18S stool	HC047.n	M2.n	P5	E03	98.6	0.04	0.17	M-103-HC047-P5-E03_S51_L001_R1_001.fastq.gz,M-103-HC047-P5-E03_S51_L001_R2_001.fastq.gz
HC048	nose		HC048.n	K2.n	P5	C07	4.9	0	0.03	K-107-HC048-P5-C07_S31_L001_R1_001.fastq.gz,K-107-HC048-P5-C07_S31_L001_R2_001.fastq.gz
HC049	stool									
HC050	nose		HC050.n	Q2.n	P3	D05	3.08	0	0.17	Q-105-HC050-1323-3-16S_S41_L001_R1_001.fastq.gz,Q-105-HC050-1323-3-16S_S41_L001_R2_001.fastq.gz
HC051	nose & stool		HC051.n	C1.n	P2	C11		0.33	2.41	C11-HC051-09b_S109_L001_R1_001.fastq,C11-HC051-09b_S109_L001_R2_001.fastq
HC052	nose		HC052.n	P2.n	P3	C01	20.27	0.09	2.17	P-101-HC052-942-3-16S_S25_L001_R1_001.fastq.gz,P-101-HC052-942-3-16S_S25_L001_R2_001.fastq.gz
HC053	nose		HC053.n	M2.n	P5	E02	28.3	0.01	0.07	M-102-HC053-P5-E02_S50_L001_R1_001.fastq.gz,M-102-HC053-P5-E02_S50_L001_R2_001.fastq.gz
HC054	nose		HC054.n	B2.n	P4	B04	1.54	0	0.11	B04-B-104-HC054_S16_L001_R1_001.fastq.gz,B04-B-104-HC054_S16_L001_R2_001.fastq.gz
HC055	nose		HC055.n	F2.n	P4	F12	8.23	0	0.09	F12-F-112-HC055_S72_L001_R1_001.fastq.gz,F12-F-112-HC055_S72_L001_R2_001.fastq.gz
HC056	nose & stool	too few reads in 18S stool	HC056.n	J2.n	P5	A09	34.1	0.01	0	J-109-HC056-P5-A09_S9_L001_R1_001.fastq.gz,J-109-HC056-P5-A09_S9_L001_R2_001.fastq.gz
HC057	nose		HC057.n	Q2.n	P3	D10	0	0	0	Q-110-HC057-958-3-16S_S46_L001_R1_001.fastq.gz,Q-110-HC057-958-3-16S_S46_L001_R2_001.fastq.gz
HC058	nose		HC058.n	A2.n	P4	A02	0.42	0	0	A02-A-102-HC058_S2_L001_R1_001.fastq.gz,A02-A-102-HC058_S2_L001_R2_001.fastq.gz
HC059	nose & stool		HC059.n	J2.n	P5	A08	29.8	0.01	0	J-108-HC059-P5-A08_S8_L001_R1_001.fastq.gz,J-108-HC059-P5-A08_S8_L001_R2_001.fastq.gz
HC060	nose & stool		HC060.n	T2.n	P9	A11	34.2	0.01	0.36	T-111-HC060-P9-A11_S83_L001_R1_001.fastq.gz,T-111-HC060-P9-A11_S83_L001_R2_001.fastq.gz
HC061	stool									
HC062	nose & stool		HC062.n	K2.n	P5	C04	61.4	0.09	0.25	K-104-HC062-P5-C04_S28_L001_R1_001.fastq.gz,K-104-HC062-P5-C04_S28_L001_R2_001.fastq.gz
HC063	nose		HC063.n	N2.n	P3	A02	0	0.02	1.02	N-102-HC063-931-3-16S_S2_L001_R1_001.fastq.gz,N-102-HC063-931-3-16S_S2_L001_R2_001.fastq.gz
HC064	nose		HC064.n	J2.n	P710	G05		0.01	0.09	P10-G05-J-105-HC064_S51_L001_R1_001.fastq.gz,P10-G05-J-105-HC064_S51_L001_R2_001.fastq.gz
HC065	nose		HC065.n	K2.n	P5	C05	14.8	0.01	0.26	K-105-HC065-P5-C05_S29_L001_R1_001.fastq.gz,K-105-HC065-P5-C05_S29_L001_R2_001.fastq.gz
HC066	nose		HC066.n	I2.n	P5	B06	25.8	0.01	0.46	I-106-HC066-P5-B06_S18_L001_R1_001.fastq.gz,I-106-HC066-P5-B06_S18_L001_R2_001.fastq.gz
HC068	nose & stool		HC068.n	I2.n	P5	B09	79.9	0.04	0.93	I-109-HC068-P5-B09_S21_L001_R1_001.fastq.gz,I-109-HC068-P5-B09_S21_L001_R2_001.fastq.gz
HC069	nose		HC069.n	N2.n	P3	A05	37.4	0.04	0.21	N-105-HC069-918-3-16S_S5_L001_R1_001.fastq.gz,N-105-HC069-918-3-16S_S5_L001_R2_001.fastq.gz
HC070	nose		HC070.n	F2.n	P4	F01	3.25	0	0.12	F01-F-101-HC070_S61_L001_R1_001.fastq.gz,F01-F-101-HC070_S61_L001_R2_001.fastq.gz
HC071	nose		HC071.n	D2.n	P4	D12	7.94	0	0.3	D12-D-112-HC071_S48_L001_R1_001.fastq.gz,D12-D-112-HC071_S48_L001_R2_001.fastq.gz
HC072	nose		HC072.n	D2.n	P4	D11	8.31	0.01	0	D11-D-111-HC072_S47_L001_R1_001.fastq.gz,D11-D-111-HC072_S47_L001_R2_001.fastq.gz
HC073	stool									
HC074	stool									
HC075	nose		HC075.n	H2.n	P9	B09	52.1	0	0.02	H-109-HC075-P9-B09_S93_L001_R1_001.fastq.gz,H-109-HC075-P9-B09_S93_L001_R2_001.fastq.gz
HC076	nose		HC076.n	J2.n	P5	A06	19.9	0	0.23	J-106-HC076-P5-A06_S6_L001_R1_001.fastq.gz,J-106-HC076-P5-A06_S6_L001_R2_001.fastq.gz
HC077	stool									
HC078	nose		HC078.n	M2.n	P5	E04	37.9	0	3.42	M-104-HC078-P5-E04_S52_L001_R1_001.fastq.gz,M-104-HC078-P5-E04_S52_L001_R2_001.fastq.gz
HC079	nose		HC079.n	N2.n	P3	A03	13.93	0	0.49	N-103-HC079-1220-3-16S_S3_L001_R1_001.fastq.gz,N-103-HC079-1220-3-16S_S3_L001_R2_001.fastq.gz
HC080	nose		HC080.n	H2.n	P9	B11	57.2	0.02	0.16	H-111-HC080-P9-B11_S95_L001_R1_001.fastq.gz,H-111-HC080-P9-B11_S95_L001_R2_001.fastq.gz
PD002	nose & stool		PD002.n	M2.n	P5	E07	33.4	0	0	M-107-PD002-P5-E07_S55_L001_R1_001.fastq.gz,M-107-PD002-P5-E07_S55_L001_R2_001.fastq.gz
PD003	stool									
PD004	nose		PD004.n	E2.n	P4	E03	17.14	0.02	0.04	E03-E-103-PD004_S51_L001_R1_001.fastq.gz,E03-E-103-PD004_S51_L001_R2_001.fastq.gz
PD005	nose		PD005.n	O2.n	P3	B05	14.25	0.04	1.18	O-105-PD005-1061-3-16S_S17_L001_R1_001.fastq.gz,O-105-PD005-1061-3-16S_S17_L001_R2_001.fastq.gz
PD006	nose		PD006.n	A2.n	P4	A08	0.3	0	0	A08-A-108-PD006_S8_L001_R1_001.fastq.gz,A08-A-108-PD006_S8_L001_R2_001.fastq.gz
PD007	nose & stool		PD007.n	C1.n	P2	C08		0.05	5.54	C08-PD007-09l_S106_L001_R1_001.fastq,C08-PD007-09l_S106_L001_R2_001.fastq
		reads were merged with PD007.n	PD007-09r.n	D1.n	P2	D03		0.02	0.73	D03-PD007-09r_S113_L001_R1_001.fastq,D03-PD007-09r_S113_L001_R2_001.fastq
		reads were merged with PD007.n	PD007-09r-2.n	F1.n	P2	F03				F03-PD007-09r-2_S137_L001_R1_001.fastq,F03-PD007-09r-2_S137_L001_R2_001.fastq
		reads were merged with PD007.n	PD007-09r-3.n	H1.n	P2	H03				H03-PD007-09r-3_S160_L001_R1_001.fastq,H03-PD007-09r-3_S160_L001_R2_001.fastq
PD008	nose		PD008.n	M2.n	P5	E09	18.9	0.07	0.95	M-109-PD008-P5-E09_S57_L001_R1_001.fastq.gz,M-109-PD008-P5-E09_S57_L001_R2_001.fastq.gz
PD010	nose		PD010.n	L2.n	P5	D08	7.6	0	0	L-108-PD010-P5-D08_S44_L001_R1_001.fastq.gz,L-108-PD010-P5-D08_S44_L001_R2_001.fastq.gz
PD011	nose & stool		PD011.n	A1.n	P2	A02		0.11	0.2	A02-PD011-09r_S76_L001_R1_001.fastq,A02-PD011-09r_S76_L001_R2_001.fastq
PD012	stool									
PD013	nose (& stool)	too few reads in 16S and 18S stool	PD013.n	C2.n	P4	C07	18.17	0.09	0.14	C07-C-107-PD013_S31_L001_R1_001.fastq.gz,C07-C-107-PD013_S31_L001_R2_001.fastq.gz
PD014	nose		PD014.n	L2.n	P5	D11	40.9	0.17	0.06	L-111-PD014-P5-D11_S47_L001_R1_001.fastq.gz,L-111-PD014-P5-D11_S47_L001_R2_001.fastq.gz
PD021	nose		PD021.n	K2.n	P5	C11	61.3	0	0.23	K-111-PD021-P5-C11_S35_L001_R1_001.fastq.gz,K-111-PD021-P5-C11_S35_L001_R2_001.fastq.gz
PD022	nose		PD022.n	N2.n	P3	A06	24.1	0	0	N-106-PD022-1291-3-16S_S6_L001_R1_001.fastq.gz,N-106-PD022-1291-3-16S_S6_L001_R2_001.fastq.gz
PD023	nose		PD023.n	L2.n	P5	D10	7	0.03	0.2	L-110-PD023-P5-D10_S46_L001_R1_001.fastq.gz,L-110-PD023-P5-D10_S46_L001_R2_001.fastq.gz
PD024	nose		PD024.n	B2.n	P4	B06	1.3	0.01	0	B06-B-106-PD024_S18_L001_R1_001.fastq.gz,B06-B-106-PD024_S18_L001_R2_001.fastq.gz
PD025	stool	too few reads in 18S stool								
PD026	nose		PD026.n	B2.n	P4	B07	4.13	0.01	4.02	B07-B-107-PD026_S19_L001_R1_001.fastq.gz,B07-B-107-PD026_S19_L001_R2_001.fastq.gz
PD027	nose		PD027.n	E2.n	P4	E02	5.5	0.04	0.06	E02-E-102-PD027_S50_L001_R1_001.fastq.gz,E02-E-102-PD027_S50_L001_R2_001.fastq.gz
PD028	nose		PD028.n	H2.n	P9	B01	88.7	0	0.15	H-101-PD028-P9-B01_S85_L001_R1_001.fastq.gz,H-101-PD028-P9-B01_S85_L001_R2_001.fastq.gz
PD029	nose & stool	too few reads in 18S stool	PD029.n	B2.n	P4	B09	11.17	0.01	0	B09-B-109-PD029_S21_L001_R1_001.fastq.gz,B09-B-109-PD029_S21_L001_R2_001.fastq.gz
PD030	nose		PD030.n	P2.n	P3	C04	27.71	0.12	0.12	P-104-PD030-1108-3-16S_S28_L001_R1_001.fastq.gz,P-104-PD030-1108-3-16S_S28_L001_R2_001.fastq.gz
PD033	nose		PD033.n	O2.n	P3	B08	9.82	0.01	0.02	O-108-PD033-1027-3-16S_S20_L001_R1_001.fastq.gz,O-108-PD033-1027-3-16S_S20_L001_R2_001.fastq.gz
PD034	nose		PD034.n	P2.n	P3	C03	12.14	0.01	0.18	P-103-PD034-834-3-16S_S27_L001_R1_001.fastq.gz,P-103-PD034-834-3-16S_S27_L001_R2_001.fastq.gz
PD035	nose & stool		PD035.n	K2.n	P5	C12	42	0.02	0.06	K-112-PD035-P5-C12_S36_L001_R1_001.fastq.gz,K-112-PD035-P5-C12_S36_L001_R2_001.fastq.gz
PD036	nose & stool		PD036.n	E1.n	P2	E08		0.05	0.25	E08-PD036-09b_S130_L001_R1_001.fastq,E08-PD036-09b_S130_L001_R2_001.fastq
PD037	nose & stool		PD037.n	A2.n	P4	A07	7.01	0.01	0.06	A07-A-107-PD037_S7_L001_R1_001.fastq.gz,A07-A-107-PD037_S7_L001_R2_001.fastq.gz
PD038	nose		PD038.n	G2.n	P4	G12	29.35	0	0.03	G12-G-112-PD038_S84_L001_R1_001.fastq.gz,G12-G-112-PD038_S84_L001_R2_001.fastq.gz
PD043	nose		PD043.n	C2.n	P4	C04	6.41	0.01	0.04	C04-C-104-PD043_S28_L001_R1_001.fastq.gz,C04-C-104-PD043_S28_L001_R2_001.fastq.gz
PD044	nose & stool		PD044.n	K2.n	P5	C10	181.7	0.86	3.25	K-110-PD044-P5-C10_S34_L001_R1_001.fastq.gz,K-110-PD044-P5-C10_S34_L001_R2_001.fastq.gz
PD045	nose		PD045.n	O2.n	P3	B04	5.42	0.04	1.58	O-104-PD045-870-3-16S_S16_L001_R1_001.fastq.gz,O-104-PD045-870-3-16S_S16_L001_R2_001.fastq.gz
PD046	nose		PD046.n	P2.n	P3	C05	26.48	0.03	0.5	P-105-PD046-797-3-16S_S29_L001_R1_001.fastq.gz,P-105-PD046-797-3-16S_S29_L001_R2_001.fastq.gz
PD051	nose		PD051.n	J2.n	P710	G01		0	0.69	P10-G01-J-101-PD051_S5_L001_R1_001.fastq.gz,P10-G01-J-101-PD051_S5_L001_R2_001.fastq.gz
PD054	nose		PD054.n	C2.n	P4	C05	24.54	0.03	0.05	C05-C-105-PD054_S29_L001_R1_001.fastq.gz,C05-C-105-PD054_S29_L001_R2_001.fastq.gz
PD055	nose & stool		PD055.n	A1.n	P2	A04		0.04	1	A04-PD055-09l_S78_L001_R1_001.fastq,A04-PD055-09l_S78_L001_R2_001.fastq
		reads were merged with PD055.n	PD055-09r.n	A1.n	P2	A11		0.03	0.24	A11-PD055-09r_S85_L001_R1_001.fastq,A11-PD055-09r_S85_L001_R2_001.fastq
PD056	nose		PD056.n	F2.n	P4	F03	15.02	0.03	1.04	F03-F-103-PD056_S63_L001_R1_001.fastq.gz,F03-F-103-PD056_S63_L001_R2_001.fastq.gz
PD057	nose		PD057.n	M2.n	P5	E08	26.1	0	0.08	M-108-PD057-P5-E08_S56_L001_R1_001.fastq.gz,M-108-PD057-P5-E08_S56_L001_R2_001.fastq.gz
PD058	nose		PD058.n	D2.n	P4	D06	1.68	0	0.07	D06-D-106-PD058_S42_L001_R1_001.fastq.gz,D06-D-106-PD058_S42_L001_R2_001.fastq.gz
PD059	nose		PD059.n	R2.n	P3	E08	17.4	0	1.79	R-108-PD059-1034-3-16S_S56_L001_R1_001.fastq.gz,R-108-PD059-1034-3-16S_S56_L001_R2_001.fastq.gz
PD060	nose		PD060.n	C2.n	P4	C06	14.45	0.02	0.14	C06-C-106-PD060_S30_L001_R1_001.fastq.gz,C06-C-106-PD060_S30_L001_R2_001.fastq.gz
PD062	nose		PD062.n	N2.n	P3	A07	42.33	0.02	15.44	N-107-PD062-1042-3-16S_S7_L001_R1_001.fastq.gz,N-107-PD062-1042-3-16S_S7_L001_R2_001.fastq.gz
PD063	nose & stool		PD063.n	N2.n	P3	A09	3.32	0.01	1.47	N-109-PD063-1232-3-16S_S9_L001_R1_001.fastq.gz,N-109-PD063-1232-3-16S_S9_L001_R2_001.fastq.gz
PD064	nose		PD064.n	J2.n	P5	A12	108.8	0	1.14	J-112-PD064-P5-A12_S12_L001_R1_001.fastq.gz,J-112-PD064-P5-A12_S12_L001_R2_001.fastq.gz
PD065	nose & stool	too few reads in 18S stool	PD065.n	T2.n	P9	A08	133.4	0.18	1.02	T-108-PD065-P9-A08_S80_L001_R1_001.fastq.gz,T-108-PD065-P9-A08_S80_L001_R2_001.fastq.gz
PD066	nose		PD066.n	I2.n	P5	B01	68.9	0.02	1.64	I-101-PD066-P5-B01_S13_L001_R1_001.fastq.gz,I-101-PD066-P5-B01_S13_L001_R2_001.fastq.gz
PD067	nose & stool		PD067.n	J2.n	P710	G03		0.02	0.13	P10-G03-J-103-PD067_S29_L001_R1_001.fastq.gz,P10-G03-J-103-PD067_S29_L001_R2_001.fastq.gz
PD068	stool									
PD069	nose & stool		PD069.n	F2.n	P4	F04	4.24	0.01	0.05	F04-F-104-PD069_S64_L001_R1_001.fastq.gz,F04-F-104-PD069_S64_L001_R2_001.fastq.gz
PD070	nose		PD070.n	I2.n	P5	B02	23.3	0	1.28	I-102-PD070-P5-B02_S14_L001_R1_001.fastq.gz,I-102-PD070-P5-B02_S14_L001_R2_001.fastq.gz
PD071	nose		PD071.n	Q2.n	P3	D12	19.07	0	0	Q-112-PD071-776-3-16S_S48_L001_R1_001.fastq.gz,Q-112-PD071-776-3-16S_S48_L001_R2_001.fastq.gz
PD072	stool	too few reads in 18S stool								
PD073	nose		PD073.n	L2.n	P5	D09	18.9	0.01	0.17	L-109-PD073-P5-D09_S45_L001_R1_001.fastq.gz,L-109-PD073-P5-D09_S45_L001_R2_001.fastq.gz
PD074	nose & stool		PD074.n	N2.n	P3	A08	13.02	0	0	N-108-PD074-998-3-16S_S8_L001_R1_001.fastq.gz,N-108-PD074-998-3-16S_S8_L001_R2_001.fastq.gz
PD075	nose & stool		PD075.n	J2.n	P5	A10	90.2	0.05	3.88	J-110-PD075-P5-A10_S10_L001_R1_001.fastq.gz,J-110-PD075-P5-A10_S10_L001_R2_001.fastq.gz
PD076	nose (& stool)	too few reads in 16S and 18S stool	PD076.n	D2.n	P4	D07	9.79	0.01	0	D07-D-107-PD076_S43_L001_R1_001.fastq.gz,D07-D-107-PD076_S43_L001_R2_001.fastq.gz
PD077	nose		PD077.n	G2.n	P4	G03	21.66	0.02	0.15	G03-G-103-PD077_S75_L001_R1_001.fastq.gz,G03-G-103-PD077_S75_L001_R2_001.fastq.gz
PD079	nose		PD079.n	H2.n	P9	B03	66.6	0.02	0.23	H-103-PD079-P9-B03_S87_L001_R1_001.fastq.gz,H-103-PD079-P9-B03_S87_L001_R2_001.fastq.gz
PD080	nose		PD080.n	T2.n	P9	A10	80.3	0.05	1	T-110-PD080-P9-A10_S82_L001_R1_001.fastq.gz,T-110-PD080-P9-A10_S82_L001_R2_001.fastq.gz
PD081	nose		PD081.n	G2.n	P4	G02	1.95	0.01	1.51	G02-G-102-PD081_S74_L001_R1_001.fastq.gz,G02-G-102-PD081_S74_L001_R2_001.fastq.gz
PD082	nose & stool		PD082.n	U2.n	P710	A10		0.32	0.01	P7-A10-U-110-PD082_S14_L001_R1_001.fastq.gz,P7-A10-U-110-PD082_S14_L001_R2_001.fastq.gz
PD083	stool									
PD084	nose		PD084.n	B2.n	P4	B08	28.57	0.14	4.48	B08-B-108-PD084_S20_L001_R1_001.fastq.gz,B08-B-108-PD084_S20_L001_R2_001.fastq.gz
PD085	nose & stool		PD085.n	D1.n	P2	D04		1.38	352.58	D04-PD085-09b_S114_L001_R1_001.fastq,D04-PD085-09b_S114_L001_R2_001.fastq
		reads were merged with PD085.n	PD085-2.n	F1.n	P2	F04				F04-PD085-09b-2_S138_L001_R1_001.fastq,F04-PD085-09b-2_S138_L001_R2_001.fastq
		reads were merged with PD085.n	PD085-3.n	H1.n	P2	H04				H04-PD085-09b-3_S161_L001_R1_001.fastq,H04-PD085-09b-3_S161_L001_R2_001.fastq
PD086	nose & stool		PD086.n	Q2.n	P3	D04	24.65	0.22	27.85	Q-104-PD086-1276-3-16S_S40_L001_R1_001.fastq.gz,Q-104-PD086-1276-3-16S_S40_L001_R2_001.fastq.gz
PD087	nose		PD087.n	P2.n	P3	C06	21.11	0	0	P-106-PD087-1368-3-16S_S30_L001_R1_001.fastq.gz,P-106-PD087-1368-3-16S_S30_L001_R2_001.fastq.gz
PD088	nose		PD088.n	O2.n	P3	B07	30.64	0.13	4.47	O-107-PD088-1212-3-16S_S19_L001_R1_001.fastq.gz,O-107-PD088-1212-3-16S_S19_L001_R2_001.fastq.gz
PD089	nose		PD089.n	V2.n	P710	B01		0.11	0.12	P7-B01-V-101-PD089_S49_L001_R1_001.fastq.gz,P7-B01-V-101-PD089_S49_L001_R2_001.fastq.gz
PD090	nose		PD090.n	T2.n	P9	A09	52.3	0.03	0	T-109-PD090-P9-A09_S81_L001_R1_001.fastq.gz,T-109-PD090-P9-A09_S81_L001_R2_001.fastq.gz
PD091	nose		PD091.n	J2.n	P5	A11	53.6	0.02	0.06	J-111-PD091-P5-A11_S11_L001_R1_001.fastq.gz,J-111-PD091-P5-A11_S11_L001_R2_001.fastq.gz
PD092	nose		PD092.n	H2.n	P9	B02	144.9	0.08	1	H-102-PD092-P9-B02_S86_L001_R1_001.fastq.gz,H-102-PD092-P9-B02_S86_L001_R2_001.fastq.gz
PD093	nose		PD093.n	A2.n	P4	A06	2.64	0	0.05	A06-A-106-PD093_S6_L001_R1_001.fastq.gz,A06-A-106-PD093_S6_L001_R2_001.fastq.gz
PD094	nose		PD094.n	F2.n	P4	F05	7.67	0.01	0.03	F05-F-105-PD094_S65_L001_R1_001.fastq.gz,F05-F-105-PD094_S65_L001_R2_001.fastq.gz
PD095	nose		PD095.n	R2.n	P3	E01	5.89	0.02	0.32	R-101-PD095-1117-3-16S_S49_L001_R1_001.fastq.gz,R-101-PD095-1117-3-16S_S49_L001_R2_001.fastq.gz
PD097	nose & stool		PD097.n	P2.n	P3	C07	29.09	0.05	0.28	P-107-PD097-811-3-16S_S31_L001_R1_001.fastq.gz,P-107-PD097-811-3-16S_S31_L001_R2_001.fastq.gz
PD098	nose		PD098.n	D2.n	P4	D05	20.82	0.03	0.73	D05-D-105-PD098_S41_L001_R1_001.fastq.gz,D05-D-105-PD098_S41_L001_R2_001.fastq.gz
PD100	nose & stool	too few reads in 18S stool	PD100.n	K2.n	P5	C09	27.2	0	0.56	K-109-PD100-P5-C09_S33_L001_R1_001.fastq.gz,K-109-PD100-P5-C09_S33_L001_R2_001.fastq.gz
RBD002	nose & stool		RBD002.n	C1.n	P2	C07		0.02	0	C07-RBD002-09r_S105_L001_R1_001.fastq,C07-RBD002-09r_S105_L001_R2_001.fastq
		reads were merged with RBD002.n	RBD002-09l.n	C1.n	P2	C10		0.03	0.38	C10-RBD002-09l_S108_L001_R1_001.fastq,C10-RBD002-09l_S108_L001_R2_001.fastq
RBD003	nose & stool	too few reads in 18S stool	RBD003.n	B2.n	P4	B10	2.14	0	0	B10-B-110-RBD003_S22_L001_R1_001.fastq.gz,B10-B-110-RBD003_S22_L001_R2_001.fastq.gz
RBD004	stool									
RBD005	stool									
RBD006	stool									
RBD007	stool	too few reads in 18S stool								
RBD008	nose & stool		RBD008.n	O2.n	P3	B09	17.53	0.8	28.34	O-109-RBD008-2111-16S_S21_L001_R1_001.fastq.gz,O-109-RBD008-2111-16S_S21_L001_R2_001.fastq.gz
RBD009	nose & stool		RBD009.n	I2.n	P5	B03	30.9	0.04	0.03	I-103-RBD009-P5-B03_S15_L001_R1_001.fastq.gz,I-103-RBD009-P5-B03_S15_L001_R2_001.fastq.gz
RBD010	stool									
RBD012	stool									
RBD013	nose & stool		RBD013.n	T2.n	P9	A01	6.6	0	0.51	T-101-RBD013-P9-A01_S73_L001_R1_001.fastq.gz,T-101-RBD013-P9-A01_S73_L001_R2_001.fastq.gz
RBD014	nose & stool	too few reads in 18S stool	RBD014-v1.n	G1.n	P2	G06		0.03	0	G06-RBD014-09b_S152_L001_R1_001.fastq,G06-RBD014-09b_S152_L001_R2_001.fastq
RBD015	stool	too few reads in 18S stool								
RBD017	nose & stool		RBD017.n	Q2.n	P3	D07	44.44	0.02	0.97	Q-107-RBD017-1929-1928-16S_S43_L001_R1_001.fastq.gz,Q-107-RBD017-1929-1928-16S_S43_L001_R2_001.fastq.gz
RBD018	nose & stool		RBD018.n	T2.n	P9	A02	36.6	0.01	2.28	T-102-RBD018-P9-A02_S74_L001_R1_001.fastq.gz,T-102-RBD018-P9-A02_S74_L001_R2_001.fastq.gz
RBD019	nose & stool		RBD019.n	D2.n	P4	D08	5.77	0	0	D08-D-108-RBD019_S44_L001_R1_001.fastq.gz,D08-D-108-RBD019_S44_L001_R2_001.fastq.gz
RBD020	stool									
RBD021	nose & stool		RBD021.n	U2.n	P710	A05		0.16	0.01	P7-A05-U-105-RBD021_S48_L001_R1_001.fastq.gz,P7-A05-U-105-RBD021_S48_L001_R2_001.fastq.gz
RBD022	nose & stool		RBD022.n	B1.n	P2	B05		0.09	0.25	B05-RBD022-09r_S91_L001_R1_001.fastq,B05-RBD022-09r_S91_L001_R2_001.fastq
		reads were merged with RBD022.n	RBD022-09l.n	B1.n	P2	B11		0.04	0	B11-RBD022-09l_S97_L001_R1_001.fastq,B11-RBD022-09l_S97_L001_R2_001.fastq
		reads were merged with RBD022.n	RBD022-09r-2.n	F1.n	P2	F06				F06-RBD022-09r-2_S140_L001_R1_001.fastq,F06-RBD022-09r-2_S140_L001_R2_001.fastq
		reads were merged with RBD022.n	RBD022-09r-3.n	H1.n	P2	H06				H06-RBD022-09r-3_S163_L001_R1_001.fastq,H06-RBD022-09r-3_S163_L001_R2_001.fastq
RBD023	stool									
RBD025	nose (& stool)	too few reads in 16S and 18S stool	RBD025.n	L2.n	P5	D12	28.4	0	0	L-112-RBD025-P5-D12_S48_L001_R1_001.fastq.gz,L-112-RBD025-P5-D12_S48_L001_R2_001.fastq.gz

ID	comment	Sample ID	extraction batch	sequencing run	sequencing coordinate	fastq Files	Forward Primer	Reverse Primer
none	mock-extraction control	exCA0D.f	A1.f	P1	A04	A04-exCFAD-16s_S4_L001_R1_001.fastq,A04-exCFAD-16s_S4_L001_R2_001.fastq	GTGBCAGCMGCCGCGGTAA	GACTACHVGGGTATCTAATCC
none	mock-extraction control	exCB0D.f	B1.f	P1	B04	B04-exCFBD-16s_S14_L001_R1_001.fastq,B04-exCFBD-16s_S14_L001_R2_001.fastq	GTGBCAGCMGCCGCGGTAA	GACTACHVGGGTATCTAATCC
none	mock-extraction control	exCA0cD.f	A1.f	P1	G08	G08-exCFAcD-16s_S65_L001_R1_001.fastq,G08-exCFAcD-16s_S65_L001_R2_001.fastq	GTGBCAGCMGCCGCGGTAA	GACTACHVGGGTATCTAATCC
none	mock-extraction control	exCB0cD.f	B1.f	P1	H08	H08-exCFBcD-16s_S73_L001_R1_001.fastq,H08-exCFBcD-16s_S73_L001_R2_001.fastq	GTGBCAGCMGCCGCGGTAA	GACTACHVGGGTATCTAATCC
none	mock-extraction control	exCE1D.f	E2.f	P11	A12	P11.A12-117-exCFE-16S_S12_L001_R1_001.fastq.gz,P11.A12-117-exCFE-16S_S12_L001_R2_001.fastq.gz	GTGBCAGCMGCCGCGGTAA	GACTACHVGGGTATCTAATCC
none	mock-extraction control	exCC1D.f	C2.f	P11	B04	P11.B04-102-exCFC-16S_S16_L001_R1_001.fastq.gz,P11.B04-102-exCFC-16S_S16_L001_R2_001.fastq.gz	GTGBCAGCMGCCGCGGTAA	GACTACHVGGGTATCTAATCC
none	mock-extraction control	elu-P11.f	NA	P11	B09	P11.B09-ntc-16S_S21_L001_R1_001.fastq.gz,P11.B09-ntc-16S_S21_L001_R2_001.fastq.gz	GTGBCAGCMGCCGCGGTAA	GACTACHVGGGTATCTAATCC
none	mock-extraction control	exCB1D.f	B2.f	P11	C02	P11.C02-111-exCFB-16S_S26_L001_R1_001.fastq.gz,P11.C02-111-exCFB-16S_S26_L001_R2_001.fastq.gz	GTGBCAGCMGCCGCGGTAA	GACTACHVGGGTATCTAATCC
none	mock-extraction control	exCA0.n	A1.n	P2	A12	A12-eCA_S86_L001_R1_001.fastq,A12-eCA_S86_L001_R2_001.fastq	GTGBCAGCMGCCGCGGTAA	GACTACHVGGGTATCTAATCC
none	mock-extraction control	exCB0.n	B1.n	P2	B12	B12-eCB_S98_L001_R1_001.fastq,B12-eCB_S98_L001_R2_001.fastq	GTGBCAGCMGCCGCGGTAA	GACTACHVGGGTATCTAATCC
none	mock-extraction control	exCC0.n	C1.n	P2	C12	C12-eCC_S110_L001_R1_001.fastq,C12-eCC_S110_L001_R2_001.fastq	GTGBCAGCMGCCGCGGTAA	GACTACHVGGGTATCTAATCC
none	mock-extraction control	exCD0.n	D1.n	P2	D12	D12-eCD_S122_L001_R1_001.fastq,D12-eCD_S122_L001_R2_001.fastq	GTGBCAGCMGCCGCGGTAA	GACTACHVGGGTATCTAATCC
none	mock-extraction control	exCE0.n	E1.n	P2	E12	E12-eCE_S134_L001_R1_001.fastq,E12-eCE_S134_L001_R2_001.fastq	GTGBCAGCMGCCGCGGTAA	GACTACHVGGGTATCTAATCC
none	mock-extraction control	exCG0.n	G1.n	P2	G10	G10-eCG_S156_L001_R1_001.fastq,G10-eCG_S156_L001_R2_001.fastq	GTGBCAGCMGCCGCGGTAA	GACTACHVGGGTATCTAATCC
none	mock-extraction control	exCN1.n	N2.n	P3	A12	N-112-exN1-16S_S12_L001_R1_001.fastq.gz,N-112-exN1-16S_S12_L001_R2_001.fastq.gz	GTGBCAGCMGCCGCGGTAA	GACTACHVGGGTATCTAATCC
none	mock-extraction control	exCO1.n	O2.n	P3	B11	O-111-exO1-16S_S23_L001_R1_001.fastq.gz,O-111-exO1-16S_S23_L001_R2_001.fastq.gz	GTGBCAGCMGCCGCGGTAA	GACTACHVGGGTATCTAATCC
none	mock-extraction control	exCP1.n	P2.n	P3	C10	P-110-exP1-16S_S34_L001_R1_001.fastq.gz,P-110-exP1-16S_S34_L001_R2_001.fastq.gz	GTGBCAGCMGCCGCGGTAA	GACTACHVGGGTATCTAATCC
none	mock-extraction control	exCQ1.n	Q2.n	P3	D09	Q-109-exQ1-16S_S45_L001_R1_001.fastq.gz,Q-109-exQ1-16S_S45_L001_R2_001.fastq.gz	GTGBCAGCMGCCGCGGTAA	GACTACHVGGGTATCTAATCC
none	mock-extraction control	exCR1.n	R2.n	P3	E02	R-102-exR1-16S_S50_L001_R1_001.fastq.gz,R-102-exR1-16S_S50_L001_R2_001.fastq.gz	GTGBCAGCMGCCGCGGTAA	GACTACHVGGGTATCTAATCC
none	mock-extraction control	exCA1.n	A2.n	P4	A01	A01-A-101_S1_L001_R1_001.fastq.gz,A01-A-101_S1_L001_R2_001.fastq.gz	GTGBCAGCMGCCGCGGTAA	GACTACHVGGGTATCTAATCC
none	mock-extraction control	exCB1.n	B2.n	P4	B12	B12-B-112_S24_L001_R1_001.fastq.gz,B12-B-112_S24_L001_R2_001.fastq.gz	GTGBCAGCMGCCGCGGTAA	GACTACHVGGGTATCTAATCC
none	mock-extraction control	exCC1.n	C2.n	P4	C11	C11-C-111_S35_L001_R1_001.fastq.gz,C11-C-111_S35_L001_R2_001.fastq.gz	GTGBCAGCMGCCGCGGTAA	GACTACHVGGGTATCTAATCC
none	mock-extraction control	exCD1.n	D2.n	P4	D10	D10-D-110_S46_L001_R1_001.fastq.gz,D10-D-110_S46_L001_R2_001.fastq.gz	GTGBCAGCMGCCGCGGTAA	GACTACHVGGGTATCTAATCC
none	mock-extraction control	exCE1.n	E2.n	P4	E09	E09-E-109_S57_L001_R1_001.fastq.gz,E09-E-109_S57_L001_R2_001.fastq.gz	GTGBCAGCMGCCGCGGTAA	GACTACHVGGGTATCTAATCC
none	mock-extraction control	exCF1.n	F2.n	P4	F08	F08-F-108_S68_L001_R1_001.fastq.gz,F08-F-108_S68_L001_R2_001.fastq.gz	GTGBCAGCMGCCGCGGTAA	GACTACHVGGGTATCTAATCC
none	mock-extraction control	exCG1.n	G2.n	P4	G07	G07-G-107_S79_L001_R1_001.fastq.gz,G07-G-107_S79_L001_R2_001.fastq.gz	GTGBCAGCMGCCGCGGTAA	GACTACHVGGGTATCTAATCC
none	mock-extraction control	exCT1.n	T2.n	P59	A04	T-104-P9-A04_S76_L001_R1_001.fastq.gz,T-104-P9-A04_S76_L001_R2_001.fastq.gz	GTGBCAGCMGCCGCGGTAA	GACTACHVGGGTATCTAATCC
none	mock-extraction control	exCI1.n	I2.n	P59	B05	I-105-P5-B05_S17_L001_R1_001.fastq.gz,I-105-P5-B05_S17_L001_R2_001.fastq.gz	GTGBCAGCMGCCGCGGTAA	GACTACHVGGGTATCTAATCC
none	mock-extraction control	exCH1.n	H2.n	P59	B06	H-106-P9-B06_S90_L001_R1_001.fastq.gz,H-106-P9-B06_S90_L001_R2_001.fastq.gz	GTGBCAGCMGCCGCGGTAA	GACTACHVGGGTATCTAATCC
none	mock-extraction control	exCK1.n	K2.n	P59	C03	K-103-P5-C03_S27_L001_R1_001.fastq.gz,K-103-P5-C03_S27_L001_R2_001.fastq.gz	GTGBCAGCMGCCGCGGTAA	GACTACHVGGGTATCTAATCC
none	mock-extraction control	exCL1.n	L2.n	P59	D02	L-102-P5-D02_S38_L001_R1_001.fastq.gz,L-102-P5-D02_S38_L001_R2_001.fastq.gz	GTGBCAGCMGCCGCGGTAA	GACTACHVGGGTATCTAATCC
none	mock-extraction control	exCM1.n	M2.n	P59	E01	M-101-P5-E01_S49_L001_R1_001.fastq.gz,M-101-P5-E01_S49_L001_R2_001.fastq.gz	GTGBCAGCMGCCGCGGTAA	GACTACHVGGGTATCTAATCC
none	mock-extraction control	exCJ1.n	J2.n	P710	G04	P10-G04-J-104_S40_L001_R1_001.fastq.gz,P10-G04-J-104_S40_L001_R2_001.fastq.gz	GTGBCAGCMGCCGCGGTAA	GACTACHVGGGTATCTAATCC
none	mock-extraction control	exCU1.n	U2.n	P710	A11	P7-A11-U-111_S26_L001_R1_001.fastq.gz,P7-A11-U-111_S26_L001_R2_001.fastq.gz	GTGBCAGCMGCCGCGGTAA	GACTACHVGGGTATCTAATCC
none	mock-extraction control	exCV1.n	V2.n	P710	B06	P7-B06-V-106_S15_L001_R1_001.fastq.gz,P7-B06-V-106_S15_L001_R2_001.fastq.gz	GTGBCAGCMGCCGCGGTAA	GACTACHVGGGTATCTAATCC
none	mock-extraction control	exCA1D.f	A2.f	P710	H03	P10-H03-FA-103-exCFA_S30_L001_R1_001.fastq.gz,P10-H03-FA-103-exCFA_S30_L001_R2_001.fastq.gz	GTGBCAGCMGCCGCGGTAA	GACTACHVGGGTATCTAATCC
none	mock-extraction control	exCB1D.f	B2.f	P12	C05	C05_111_exCFB_18S_GCGTAGTA-TCGACTAG_L001_R1_001.fastq,C05_111_exCFB_18S_GCGTAGTA-TCGACTAG_L001_R2_001.fastq	CGGTAAYTCCAGCTCYV	CCGTCAATTHCTTYAART
none	mock-extraction control	exCC1D.f	C2.f	P13	B01	P13B01-102-exCFC_S49_L001_R1_001.fastq,P13B01-102-exCFC_S49_L001_R2_001.fastq	CGGTAAYTCCAGCTCYV	CCGTCAATTHCTTYAART
none	mock-extraction control	exCE1D.f	E2.f	P12	C07	C07_117_exCFE_18S_TACGCTGC-TCGACTAG_L001_R1_001.fastq,C07_117_exCFE_18S_TACGCTGC-TCGACTAG_L001_R2_001.fastq	CGGTAAYTCCAGCTCYV	CCGTCAATTHCTTYAART
none	nasal wash solution	saline09	Saline	P2	G11	G11-saline09_S157_L001_R1_001.fastq,G11-saline09_S157_L001_R2_001.fastq	GTGBCAGCMGCCGCGGTAA	GACTACHVGGGTATCTAATCC
none	no template control	NTC-P1	NA	P1	H09	H09-NTC-16s_S74_L001_R1_001.fastq,H09-NTC-16s_S74_L001_R2_001.fastq	GTGBCAGCMGCCGCGGTAA	GACTACHVGGGTATCTAATCC
none	no template control	NTC-P2	NA	P2	F02	F02-NTC_S136_L001_R1_001.fastq,F02-NTC_S136_L001_R2_001.fastq	GTGBCAGCMGCCGCGGTAA	GACTACHVGGGTATCTAATCC
none	no template control	NTC-P3	NA	P3	F10	S-110-16S_S70_L001_R1_001.fastq.gz,S-110-16S_S70_L001_R2_001.fastq.gz	GTGBCAGCMGCCGCGGTAA	GACTACHVGGGTATCTAATCC
none	reagent control	elu-P2.n	NA	P2	F07	F07-elu_S141_L001_R1_001.fastq,F07-elu_S141_L001_R2_001.fastq	GTGBCAGCMGCCGCGGTAA	GACTACHVGGGTATCTAATCC
none	reagent control	elu-P3-1.n	NA	P3	D03	NTC-16S_S39_L001_R1_001.fastq.gz,NTC-16S_S39_L001_R2_001.fastq.gz	GTGBCAGCMGCCGCGGTAA	GACTACHVGGGTATCTAATCC
none	reagent control	elu-P3-2.n	NA	P3	H01	NTC2-16S_S73_L001_R1_001.fastq.gz,NTC2-16S_S73_L001_R2_001.fastq.gz	GTGBCAGCMGCCGCGGTAA	GACTACHVGGGTATCTAATCC
none	reagent control	elu-P4-1.n	NA	P4	E05	E05-E-105_S53_L001_R1_001.fastq.gz,E05-E-105_S53_L001_R2_001.fastq.gz	GTGBCAGCMGCCGCGGTAA	GACTACHVGGGTATCTAATCC
none	reagent control	elu-P4-2.n	NA	P4	H01	H01-NTC_S85_L001_R1_001.fastq.gz,H01-NTC_S85_L001_R2_001.fastq.gz	GTGBCAGCMGCCGCGGTAA	GACTACHVGGGTATCTAATCC
none	reagent control	elu-P5o.n	NA	P59	C01	NTCo-P9-C01_S69_L001_R1_001.fastq.gz,NTCo-P9-C01_S69_L001_R2_001.fastq.gz	GTGBCAGCMGCCGCGGTAA	GACTACHVGGGTATCTAATCC
none	reagent control	elu-P5n.n	NA	P59	C02	NTCn-P9-C02_S70_L001_R1_001.fastq.gz,NTCn-P9-C02_S70_L001_R2_001.fastq.gz	GTGBCAGCMGCCGCGGTAA	GACTACHVGGGTATCTAATCC
none	reagent control	elu-P7n.n	NA	P710	G06	P10-G06-NTCn_S61_L001_R1_001.fastq.gz,P10-G06-NTCn_S61_L001_R2_001.fastq.gz	GTGBCAGCMGCCGCGGTAA	GACTACHVGGGTATCTAATCC
none	reagent control	elu-P7.n	NA	P710	C01	P7-C01-NTC_S4_L001_R1_001.fastq.gz,P7-C01-NTC_S4_L001_R2_001.fastq.gz	GTGBCAGCMGCCGCGGTAA	GACTACHVGGGTATCTAATCC
none	reagent control	elu-P13.f	NA	P13	B06	P13B06-ntc_S54_L001_R1_001.fastq,P13B06-ntc_S54_L001_R2_001.fastq	CGGTAAYTCCAGCTCYV	CCGTCAATTHCTTYAART
generous donor A	internal standard	GDAcD-P1	GDA	P1	A09	A09-GDAcD-16s_S9_L001_R1_001.fastq,A09-GDAcD-16s_S9_L001_R2_001.fastq	GTGBCAGCMGCCGCGGTAA	GACTACHVGGGTATCTAATCC
generous donor A	internal standard	GDA-2-P1	GDA	P1	A10	A10-GDAD-16s_S10_L001_R1_001.fastq,A10-GDAD-16s_S10_L001_R2_001.fastq	GTGBCAGCMGCCGCGGTAA	GACTACHVGGGTATCTAATCC
generous donor A	internal standard	GDA-0.2-P1	GDA	P1	B10	B10-GDAD0-1-16s_S20_L001_R1_001.fastq,B10-GDAD0-1-16s_S20_L001_R2_001.fastq	GTGBCAGCMGCCGCGGTAA	GACTACHVGGGTATCTAATCC
generous donor A	internal standard	GDA-0.02-P1	GDA	P1	C10	C10-GDAD0-01-16s_S30_L001_R1_001.fastq,C10-GDAD0-01-16s_S30_L001_R2_001.fastq	GTGBCAGCMGCCGCGGTAA	GACTACHVGGGTATCTAATCC
generous donor A	internal standard	GDA-0.002-P1	GDA	P1	D10	D10-GDAD0001-16s_S40_L001_R1_001.fastq,D10-GDAD0001-16s_S40_L001_R2_001.fastq	GTGBCAGCMGCCGCGGTAA	GACTACHVGGGTATCTAATCC
generous donor A	internal standard	GDA1-P11	GDA	P11	E03	P11.E03-GDA1-16S_S51_L001_R1_001.fastq.gz,P11.E03-GDA1-16S_S51_L001_R2_001.fastq.gz	GTGBCAGCMGCCGCGGTAA	GACTACHVGGGTATCTAATCC
generous donor A	internal standard	GDA2-P11	GDA	P11	F03	P11.F03-GDA2-16S_S62_L001_R1_001.fastq.gz,P11.F03-GDA2-16S_S62_L001_R2_001.fastq.gz	GTGBCAGCMGCCGCGGTAA	GACTACHVGGGTATCTAATCC
generous donor A	internal standard	GDA2-P2	GDA	P2	H09	H09-GD1_S165_L001_R1_001.fastq,H09-GD1_S165_L001_R2_001.fastq	GTGBCAGCMGCCGCGGTAA	GACTACHVGGGTATCTAATCC
generous donor A	internal standard	GDA0.3-P3	GDA	P3	H02	GDA0-3-16S_S74_L001_R1_001.fastq.gz,GDA0-3-16S_S74_L001_R2_001.fastq.gz	GTGBCAGCMGCCGCGGTAA	GACTACHVGGGTATCTAATCC
generous donor A	internal standard	GDA0.03-P3	GDA	P3	H03	GDA0-03-16S_S75_L001_R1_001.fastq.gz,GDA0-03-16S_S75_L001_R2_001.fastq.gz	GTGBCAGCMGCCGCGGTAA	GACTACHVGGGTATCTAATCC
generous donor A	internal standard	GDA0.3-P4	GDA	P4	H02	H02-GDA0-3_S86_L001_R1_001.fastq.gz,H02-GDA0-3_S86_L001_R2_001.fastq.gz	GTGBCAGCMGCCGCGGTAA	GACTACHVGGGTATCTAATCC
generous donor A	internal standard	GDA0.03-P4	GDA	P4	H03	H03-GDA0-03_S87_L001_R1_001.fastq.gz,H03-GDA0-03_S87_L001_R2_001.fastq.gz	GTGBCAGCMGCCGCGGTAA	GACTACHVGGGTATCTAATCC
generous donor A	internal standard	GDA0.3-P10	GDA	P710	G07	P10-G07-GDA0-3_S71_L001_R1_001.fastq.gz,P10-G07-GDA0-3_S71_L001_R2_001.fastq.gz	GTGBCAGCMGCCGCGGTAA	GACTACHVGGGTATCTAATCC
generous donor A	internal standard	GDA0.03-P10	GDA	P710	G08	P10-G08-GDA0-03_S81_L001_R1_001.fastq.gz,P10-G08-GDA0-03_S81_L001_R2_001.fastq.gz	GTGBCAGCMGCCGCGGTAA	GACTACHVGGGTATCTAATCC
generous donor A	internal standard	GDA0.3-P7	GDA	P710	C02	P7-C02-GDA0-3_S16_L001_R1_001.fastq.gz,P7-C02-GDA0-3_S16_L001_R2_001.fastq.gz	GTGBCAGCMGCCGCGGTAA	GACTACHVGGGTATCTAATCC
generous donor A	internal standard	GDA0.03-P7	GDA	P710	C03	P7-C03-GDA0-03_S28_L001_R1_001.fastq.gz,P7-C03-GDA0-03_S28_L001_R2_001.fastq.gz	GTGBCAGCMGCCGCGGTAA	GACTACHVGGGTATCTAATCC
generous donor A	internal standard	GDA1-P10	GDA	P710	H11	P10-H11-GDA1-GDA1_S31_L001_R1_001.fastq.gz,P10-H11-GDA1-GDA1_S31_L001_R2_001.fastq.gz	GTGBCAGCMGCCGCGGTAA	GACTACHVGGGTATCTAATCC
generous donor A	internal standard	GDA2-P10	GDA	P710	H12	P10-H12-GDA2-GDA2_S42_L001_R1_001.fastq.gz,P10-H12-GDA2-GDA2_S42_L001_R2_001.fastq.gz	GTGBCAGCMGCCGCGGTAA	GACTACHVGGGTATCTAATCC
generous donor A	internal standard	GDA0.3-P9	GDA	P9	C03	GDA0-3-P9-C03_S71_L001_R1_001.fastq.gz,GDA0-3-P9-C03_S71_L001_R2_001.fastq.gz	GTGBCAGCMGCCGCGGTAA	GACTACHVGGGTATCTAATCC
generous donor A	internal standard	GDA0.03-P9	GDA	P9	C04	GDA0-03-P9-C04_S72_L001_R1_001.fastq.gz,GDA0-03-P9-C04_S72_L001_R2_001.fastq.gz	GTGBCAGCMGCCGCGGTAA	GACTACHVGGGTATCTAATCC
generous donor A	internal standard	GDA1-P12	GDA	P12	F03	F03__GDA1_18S_ACTCGCTA-GCGTAAGA_L001_R1_001.fastq,F03__GDA1_18S_ACTCGCTA-GCGTAAGA_L001_R2_001.fastq	CGGTAAYTCCAGCTCYV	CCGTCAATTHCTTYAART
generous donor A	internal standard	GDA1-P13	GDA	P13	F03	P13F03-GDA1_S79_L001_R1_001.fastq,P13F03-GDA1_S79_L001_R2_001.fastq	CGGTAAYTCCAGCTCYV	CCGTCAATTHCTTYAART
generous donor A	internal standard	GDA2-P12	GDA	P12	E03	E03__GDA2_18S_ACTCGCTA-CCTAGAGT_L001_R1_001.fastq,E03__GDA2_18S_ACTCGCTA-CCTAGAGT_L001_R2_001.fastq	CGGTAAYTCCAGCTCYV	CCGTCAATTHCTTYAART
generous donor A	internal standard	GDA2-P13	GDA	P13	E03	P13E03-GDA2_S73_L001_R1_001.fastq,P13E03-GDA2_S73_L001_R2_001.fastq	CGGTAAYTCCAGCTCYV	CCGTCAATTHCTTYAART
